# Systematic analysis of network-driven adaptive resistance to CDK4/6 and oestrogen receptor inhibition using meta-dynamic network modelling

**DOI:** 10.7554/eLife.87710

**Published:** 2026-08-27

**Authors:** Anthony Hart, Sung-Young Shin, Lan K Nguyen

**Affiliations:** 1 Department of Biochemistry and Molecular Biology, Faculty of Medicine, Nursing and Health Sciences, Monash University Clayton Australia; 2 Biomedicine Discovery Institute, Monash University Clayton Australia; 3 Computational Systems Oncology Program, South Australian immunoGENomics Cancer Institute (SAiGENCI), The University of Adelaide Adelaide Australia; 4 Australian Research Council Centre of Excellence for the Mathematical Analysis of Cellular Systems (MACSYS) Parkville Australia; Universidad Nacional Autónoma de México Mexico; King's College London United Kingdom

**Keywords:** adaptive resistance, heterogeneity, protein signalling, computational modelling, CDK4/6 inhibition, Human

## Abstract

Drug resistance inevitably emerges during the treatment of cancer by targeted therapy. Adaptive resistance is a major form of drug resistance, wherein the rewiring of protein signalling networks in response to drug perturbation allows drug-targeted protein activity to recover. This can occur in the continuous presence of the drug and enables cells to survive/grow. Simultaneously, molecular heterogeneity enables the selection of drug-resistant cancer clones that can survive an initial drug insult, proliferate, and eventually cause disease relapse. Despite their importance, the link between heterogeneity and adaptive resistance, specifically how heterogeneity influences protein signalling dynamics to drive adaptive resistance, remains poorly understood. Here, we have explored the relationship between heterogeneity, protein signalling dynamics, and adaptive resistance through the development of a novel modelling technique coined Meta Dynamic Network (MDN) modelling. We use MDN modelling to characterise how heterogeneity influences the drug-response signalling dynamics of the proteins that regulate early cell cycle progression and demonstrate that heterogeneity can robustly facilitate adaptive resistance associated dynamics for key cell cycle regulators. We determined the influence of heterogeneity at the level of both reaction coefficients and protein abundance and show that reaction coefficients are a much stronger driver of adaptive resistance. Owing to the mechanistic nature of the underpinning ordinary differential equation framework, we then identified a full spectrum of subnetworks capable of driving adaptive resistance dynamics in the key early cell cycle regulators. Finally, we show that single-cell dynamic data supports the validity of our MDN modelling technique and a comparison between our predicted resistance mechanisms and known CDK4/6 and oestrogen receptor inhibitor resistance mechanisms suggests MDN modelling can be deployed to robustly predict network-level resistance mechanisms for novel drugs and additional protein signalling networks.

## Introduction

Drug resistance is a widespread phenomenon across all cancer types and is a major obstacle to the development of curative therapeutic strategies (Vasan et al., 2019; Wang et al., 2019; Pich et al., 2022). Cellular heterogeneity is a known driver of drug resistance, wherein the treatment of a heterogenous population of cancer cells with cytostatic or cytotoxic drugs creates a selective pressure that results in the survival and expansion of any cells that are capable of overcoming the effects of said drugs (Knudsen et al., 2022; Jubran et al., 2022; Dagogo-Jack and Shaw, 2018). While this general phenomenon is well established (Turke et al., 2010; Patel et al., 2022; Cassidy, 2019), it is usually less clear exactly how and why some cells are able to overcome a drug treatment and others are not. Heterogeneity is a catch-all term used to describe any and all differences between cells; however, it could be argued that the majority of the differences between cells ultimately converge on differences in how their constituent proteins behave, that is their protein dynamics. To deepen our understanding of how tumour heterogeneity drives drug resistance, we must explore the relationship between cellular heterogeneity, protein dynamics, and drug resistance.

The efficacy of a targeted therapy largely comes down to how well and how long the targeted protein is suppressed, and how frequently and reliably this suppression results in either cytostasis or apoptosis. A cell can therefore be considered resistant if the target protein is insufficiently suppressed or is initially suppressed but later recovers, or if the suppression of the protein is insufficient to stimulate cytostasis or apoptosis (Herrera-Abreu et al., 2016; Ahmed et al., 2019). Proteins are embedded in complex networks, and protein dynamics are dictated by the properties of the networks in which they reside. While resistance is often due to direct effects, such as reduced drug-target binding affinity (Blombery et al., 2019; Yun et al., 2008) or excessive drug efflux from tumour cells (Xue and Liang, 2012; Smyth et al., 1998), it can also occur due to changes in the state of the biochemical networks of a cell that counteract the effects of the drug; a phenomenon referred to as adaptive resistance (Jin et al., 2020; Li et al., 2022; Cremers and Nguyen, 2019). PI3K, EGFR, and CDK4/6 are just few prime examples of proteins that have been targeted in the treatment of cancer that display acute and robust adaptive resistance (Wright et al., 2021; Herrera-Abreu et al., 2016; Ma et al., 2016). These studies also support the notion that it is rarely the behaviour of a single ‘gatekeeper’ protein that drives adaptive resistance, but an entire protein network that acts and is acted upon by the target protein (Park et al., 2020; Nguyen and Kholodenko, 2016). Frequently, however, we possess a limited knowledge and appreciation of the network-level mechanistic relationships that underpin adaptive resistance.

Understanding the relationship between cellular heterogeneity and adaptive drug resistance requires an ability to explore and characterise the heterogeneity that exists both within a tumour and between patients. This can be achieved in silico by investigating the behaviour of a network, that is the dynamics of its constituent proteins, over a broad range of network conditions, allowing us to observe the full spectrum of possible dynamics that can be displayed by a given network topology. A particularly useful technique for investigating protein signalling networks is ordinary differential equation (ODE) modelling (Bachmann et al., 2012). An ODE model is a mathematical representation of how the protein species within a network interact and evolve over time. ODE models have been extensively used to simulate and predict network-level responses to perturbations, such as growth-factor stimulation or drug treatment (Shankar et al., 2019; Clarke and Fisher, 2020; Altrock et al., 2015; Ghomlaghi et al., 2021). In an ODE model, the interactions between the network’s constituent proteins are converted into mathematical formulations using well-established biochemical rate laws (Tyson et al., 2019; Tandon et al., 2022; Deuflhard and Röblitz, 2015; Kearney et al., 2021; Shin et al., 2018). The end result is a set of ODEs – the model – that can be numerically solved using specialised ODE solvers, allowing one to simulate, and thus predict, how the concentrations of the protein species will change over time or in response to perturbations (Tyson et al., 2019; Tandon et al., 2022; Deuflhard and Röblitz, 2015; Kearney et al., 2021; Shin et al., 2018).

An ODE model is comprised of state variables, initial conditions (ICs), model parameters, and defined inputs and outputs (Tyson et al., 2019; Tandon et al., 2022; Deuflhard and Röblitz, 2015). Typically, the parameters, or reaction coefficients, represent the strength of protein interactions and state variables correspond to the concentrations of the protein species. ICs are the values of the state variables at the starting point of a simulation and represent the total abundance levels of the protein species within the model. Model input(s) are usually a perturbation to the modelled network, such as a growth factor stimulation or a drug inhibition. We can model network heterogeneity by producing a suite of ODE models, each with their own set of parameter values and/or ICs. In this manner, each model ‘instance’ represents a unique cellular context and produces a unique set of dynamic behaviours for its constituent proteins. By varying the parameters and ICs over wide ranges, we can delineate an extreme upper limit to the heterogeneity in dynamics facilitated by a network topology.

The process of creating models almost always involves a degree of abstraction, either explicitly, due to conscious decisions by the creator, or implicitly, due to imperfect knowledge of the system being modelled. This abstraction forces a model’s parameters to capture information beyond that which is being explicitly modelled and can potentially render the concept of a ‘true’ or ‘biologically accurate’ parameter value less meaningful. There is an array of factors that can influence reaction coefficients: post-translational modifications, catalysts/scaffolds, pH, etc., and they can all individually influence reaction coefficients over several orders of magnitude. In the context of cancer, this variability in reaction coefficients can become extreme, with mutations altering the properties of proteins and dramatically changing how strongly they interact with other proteins. By varying the parameters and ICs over wide ranges, we also potentially capture protein dynamics driven by factors we have not explicitly modelled.

Drug resistance is a problem almost as old as drug treatments, and many computational modelling frameworks have been developed to investigate the emergence of drug resistance during cancer treatment (Sun and Hu, 2018; Chisholm et al., 2016; Belkhir et al., 2021). A comprehensive review of the modelling frameworks developed to explore the relationship between cellular heterogeneity and drug resistance, in particular, was written by Chisholm et al., 2016. Most of the models covered within focused on population dynamics and were used to identify key processes that govern how resistance emerges within a heterogenous cell population. Notably, none of the models in this review attempted to link heterogenous intracellular processes with adaptive resistance mechanisms, or resistance in general. Perhaps most similar to the ensemble approach developed within this project, He et al. developed an ODE-based modelling technique termed RACIPE (random circuit perturbation; Huang et al., 2018). In contrast to our work, RACIPE was a quasi-Monte Carlo method developed to alleviate the necessity of identifying precise kinetic parameter values on which model predictions are supposedly dependent. While their technique does involve the exploration of a range of parameter values, it does so to make predictions about broadly robust outcomes in gene regulatory circuits, not to link parameter variation to heterogenous protein network dynamics. Thus, there is a need for a novel computational framework capable of systematically characterising network-dynamic heterogeneity and its relationship with adaptive resistance.

To address this need, we have developed a new modelling framework, coined Meta-Dynamic Network (MDN) modelling, which enables us to study the effects of network heterogeneity on protein signalling dynamics. In this paper we have applied MDN modelling to an early cell cycle (ECC) network model to systematically explore and illuminate adaptive resistance mechanisms that arise in response to targeted CDK4/6 and oestrogen receptor (ER) inhibition. The ECC network model developed herein incorporates the two major mitogenic signalling pathways that are largely responsible for cell cycle initiation, and the G1-S phase cell cycle regulatory network.

Using MDN modelling and the ECC network model, we have demonstrated that variation in both the strength of reaction coefficients (parameters) and the total abundance of proteins (IC values) can affect the qualitative shape and features of a network’s protein dynamics. Furthermore, we provide evidence that parameter variation induces a much greater variety in the qualitative shape and features of the network’s protein dynamics and is therefore much more likely to give rise to adaptive resistance dynamics. We also show that there is a surprisingly large number of contexts in which the network topology of the ECC network can facilitate resistance dynamics and that we can dissect these contexts to systematically characterise their underpinning kinetic drivers. Finally, we analysed existing publicly available biological data to qualitatively validate the existence of a spectrum of signalling dynamics in response to CDK4/6 inhibitors and propose a novel relationship between population signalling dynamics and population level drug sensitivity.

## Results

### An overview of Meta-Dynamic Network (MDN) modelling

To explore the link between cellular heterogeneity, protein dynamics, and drug resistance, we first developed a novel modelling framework coined MDN modelling. The principal purpose of the MDN modelling framework is to explore how parametric and IC variation influence the qualitative shape of protein dynamics within a given network. By varying model parameters and *IC* values, we are able to capture many sources of cellular heterogeneity and characterise their influence on protein signalling dynamics. Varying parameter values captures heterogeneity in the interactions between proteins that arises due to phenomena such as cell-specific mutations, post-translational modifications, and epigenetic modifications. Varying IC values captures heterogeneity in protein concentration that arises due to phenomena such as stochastic protein expression, copy number variation, and tissue-specific expression programmes.

**Figure 1.**
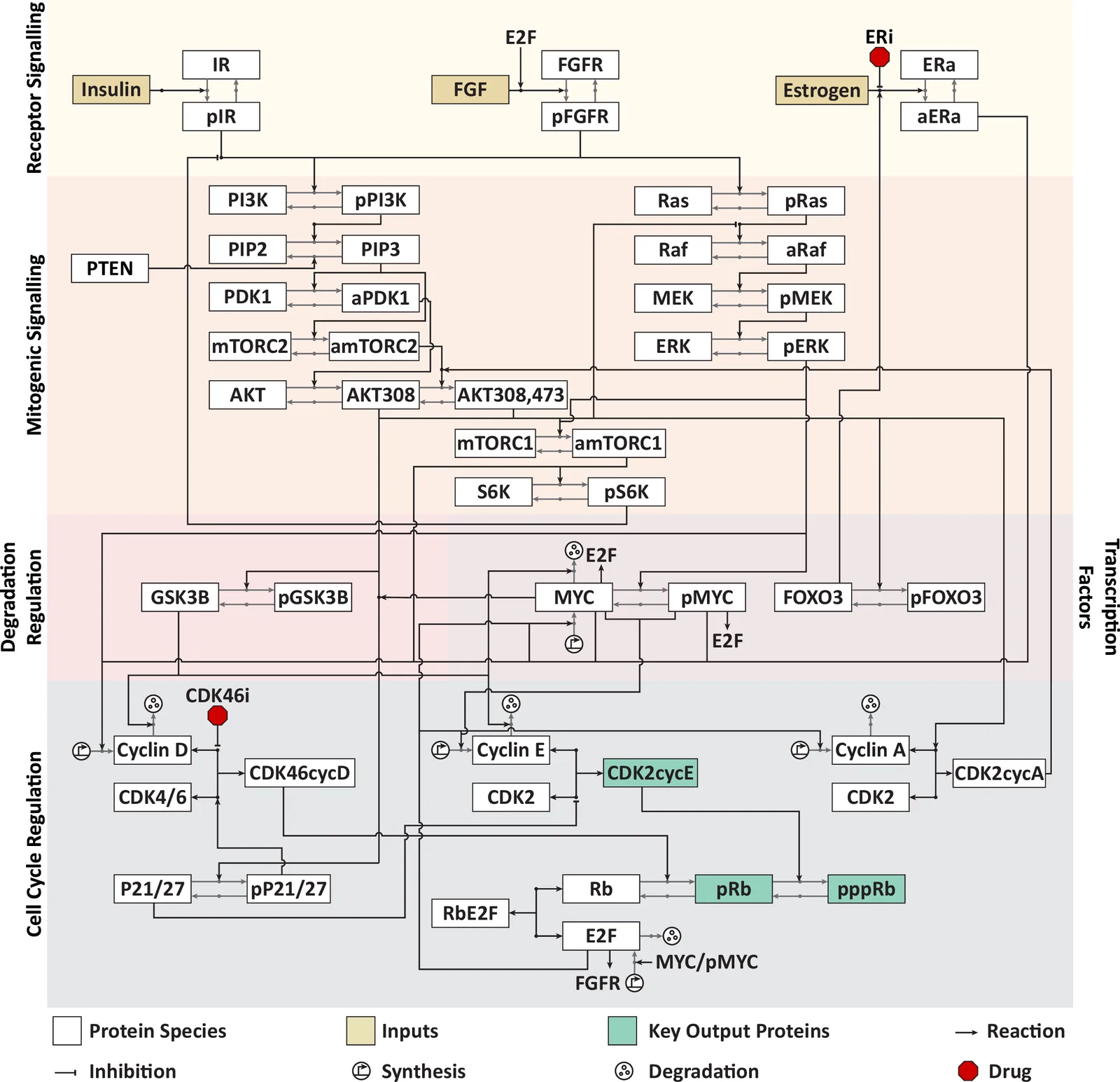
A workflow of the Meta-Dynamic Network (MDN) modelling pipeline. As with all modelling, the first step is asking a useful question. In our case, this is usually related to understanding complex protein signalling dynamics. The second step is defining the network; this involves selecting nodes and identifying relationships between them. The third step is translating the network schematic to a mathematical representation, which is typically a combination of ordinary differential equations and biochemical rate laws. The fourth step is the generation of thousands of model instances, each with a unique set of properties, for example parameter and initial condition values. The fifth step is the simulation of each model instance and the bulk analysis of the simulations. In this work, we analyse the distribution of the qualitative dynamics of the network’s protein species.

Once a large number of unique model instances have been generated, each with their own unique parameter and/or IC values, we can simulate each model’s response to drug treatment and analyse the resulting shape of each protein’s dynamic. From this analysis, we can determine if there are contexts where key output proteins, such as downstream targets of the drug perturbation, demonstrate protein dynamics associated with adaptive resistance. Finally, we can group model instances that display similar dynamics together and perform further analyses to systematically identify the mechanistic causes capable of driving adaptive resistance dynamics. An overview of the MDN modelling process can be seen in Figure 1.

Protein dynamics that are associated with adaptive resistance are those where the protein is insufficiently suppressed over a sustained time period, that is increasing, biphasic, and rebound (Cremers and Nguyen, 2019; Wright et al., 2021; Ma et al., 2016; Park et al., 2020; Ahmed et al., 2019). Increasing is the most dramatic adaptive-resistance response, where the drug perturbation not only fails to suppress key output proteins but also actually increases their activity/expression. Biphasic is similar to increasing in its initial phase, where the outputs actually increase; however, the degree of the second decreasing phase likely influences the degree of resistance conferred by this particular behaviour. Rebound is similar to biphasic wherein the degree of resistance conferred by this dynamic is likely influenced by how strongly the protein recovers. Although rebound is probably the most commonly observed adaptive-resistance driving dynamic (Cremers and Nguyen, 2019; Wright et al., 2021; Herrera-Abreu et al., 2016; Ma et al., 2016; Park et al., 2020; Nguyen and Kholodenko, 2016), for the purpose of this project we consider all three of these behaviours as adaptive-resistance associated behaviours and consider decreasing as the only sensitivity associated behaviour.

### Construction of a mechanistic model of the early cell cycle (ECC) network

**Figure 2.**
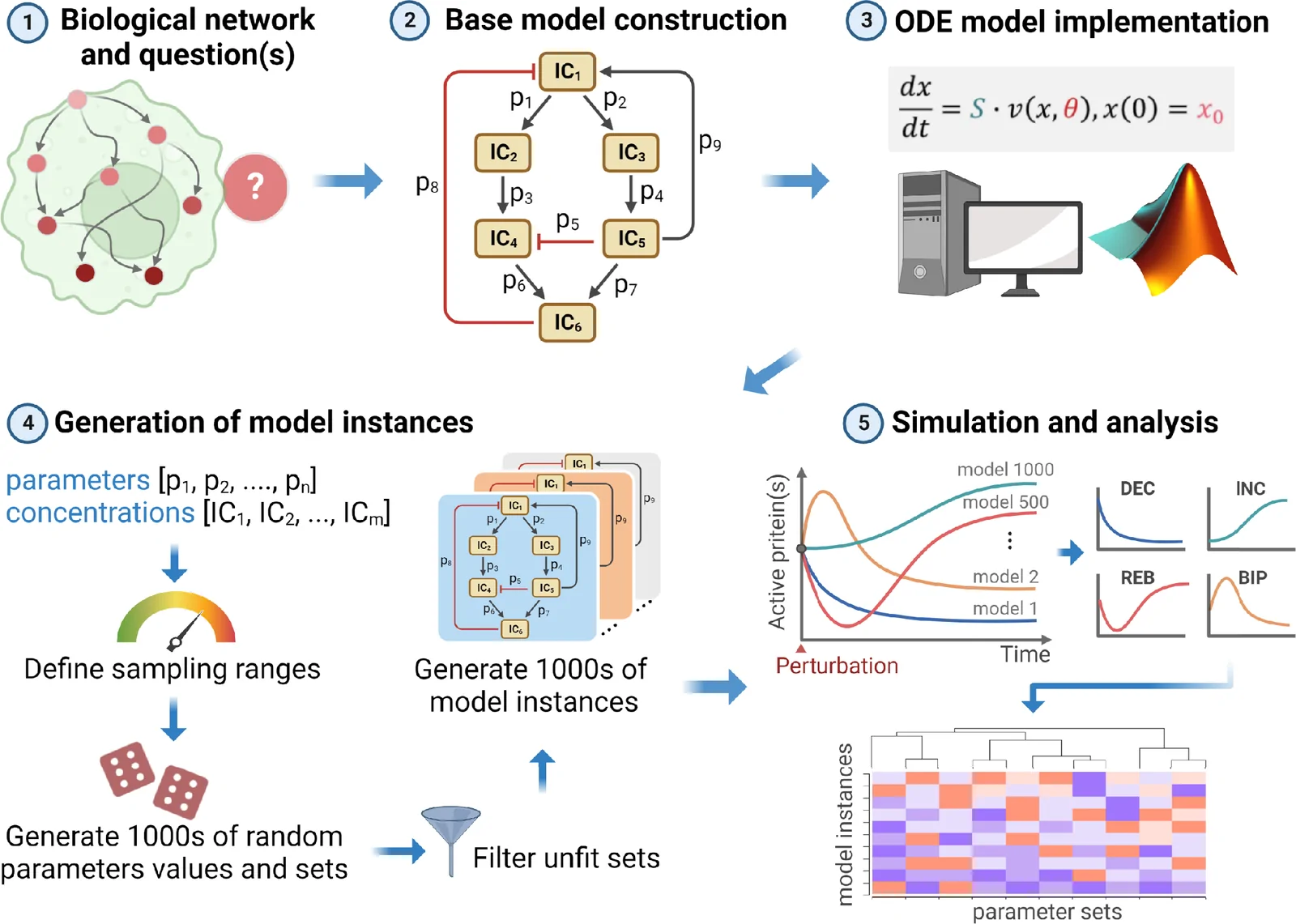
Network schematic of the early cell cycle (ECC) network. Network schematic showing the detailed biochemical reactions that regulate the initiation of the G1 phase and transition through to the S phase of the cell cycle. The network includes three mitogenic inputs, two mitogenic signalling pathways, PI3K and MAPK, key transcription factors that promote G1 phase transition, key CDK-cyclin complexes and their regulators, a CDK4/6 inhibitor, and an estrogen receptor (ER) inhibitor. Canonically, mitogenic stimulation leads to the cascading activation of the mitogenic signalling pathways, which converge on the synthesis of cyclin D. The synthesis of cyclin D then promotes the activation of CDK4/6, which consequently drives the synthesis of cyclin E. Cyclin E activates CDK2, leading to the synthesis of cyclin A and the formation of CDK2-cyclin A complexes signals the initiation of S phase. ER and CDK4/6 inhibitors are believed to disrupt this linear chain of events and thus prevent proliferation.

The ECC network model generated in this project was built to capture the signalling events driving entry into G1 and progression through the ECC events, up to the G1-S phase transition. A detailed network schematic of the ECC’s biochemical reactions can be seen in Figure 2. We nominally include two tyrosine kinase receptors, the insulin receptor (INSR) and the fibroblast growth factor receptor (FGFR), as they strongly stimulate the PI3K and MAPK pathways respectively but are both capable of stimulating each pathway.

The two major mitogenic signalling pathways, PI3K and MAPK, were included for three reasons. The first is that they are known to potently stimulate the production of cyclin D, the primary activator of CDK4/6 (Chen et al., 2016; Wang et al., 2014). The second is that activating mutations in these pathways are some of the most common mutations across all cancer types and the final reason is that these same mutations have been shown to facilitate resistance to CDK4/6 inhibitors (Del Re et al., 2021; Samuels and Velculescu, 2004). The inclusion of proteins within this network is not exhaustive, due to computational limitations, but our model captures the core signalling relationships and network structure. The final component included in this model was the network regulating G1 entry and progression into S phase. This includes the relevant CDKs, cyclins and their inhibitors, as well as the transcription machinery regulating cyclin expression. Clinical approval for the use of CDK4/6 inhibitors has traditionally been limited to patients with ER+ breast cancer who were concurrently receiving ER antagonists but were still experiencing disease progression (FDA, 2017a; FDA, 2017b; FDA, 2021). Due to the ER’s ability to promote CDK4/6 inhibition efficacy and the clinical practice of simultaneously prescribing ER antagonists with CDK4/6 inhibitors (FDA, 2017a; FDA, 2017b; FDA, 2021), the ER and its downstream signalling events were also included in the model.

The ECC network model was constructed using ODEs that represent biochemical interactions as a series of ODEs based on established kinetic laws. More detailed description of the model is given in the *Materials and methods*. The full ODE equations and reaction rates are given in Supplementary files 1–4.

### The ECC network robustly facilitates resistance-associated protein dynamics

To investigate the meta-dynamics of the ECC network, that is the range of dynamics that can be facilitated by the topology of the ECC network, we first generated 100,000 unique model instances. Each model instance possessed an identical set of ICs along with a unique set of parameter values. The parameter values for each model instance were generated by randomly selecting values for each parameter between the range 10^–5^ and 10^4^. See Figure 3—figure supplement 1A for a visual representation of the model generation process. Each model instance was simulated as laid out in the *ODE model construction, modelling, and simulations* (see *Materials and methods*), and the resulting time-course drug response of each of the network’s proteins was stored. This was repeated for all model instances. Alongside this process we also performed an experiment to test if novel, randomly drawn sets of model instances produced altered distributions of dynamics. Selecting new sets of 100,000 model instances produced near identical distributions of dynamics giving us confidence the distribution of protein dynamics converges at this set size. Details of this experiment can be found in the *Appendix*.

**Figure 3.**
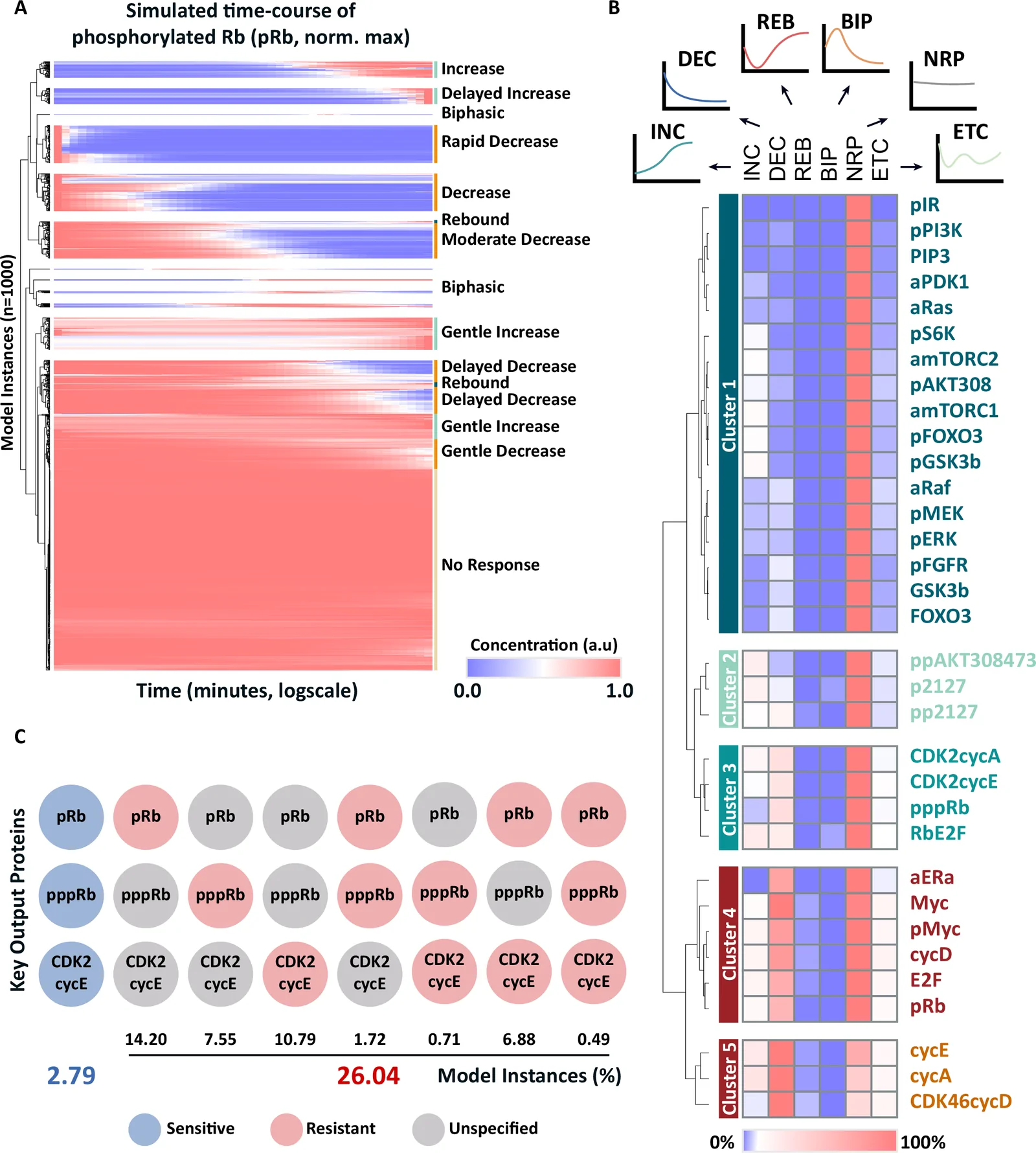
The ECC network robustly facilitates resistance-associated protein dynamics. (**A**) Clustered heatmap of the time-course profiles of a representative subgroup model instances, normalised to maximum value. Time-course profiles are of mono-phosphorylated Rb (pRb), the downstream target of active CDK4/6, and in response to CDK4/6 and ER inhibition. Blue represents low concentration and red represents high concentration. (**B**) The frequency of the six dynamic categories for the model’s active protein forms, across 100,000 model instances. Blue represents low frequency and red represents high frequency. Species have been clustered to highlight species that have highly correlated distributions of dynamics. (**C**) The frequency of model instances displaying simultaneous sensitivity (blue), or resistance in at least one of the key output proteins (red). Only 2.79% of model instances show simultaneous sensitivity across all three key output proteins. The percentage of resistant model instances was calculated by measuring the number of unique model instances that show resistance-associated dynamics for at least one of the key output proteins.

We found that parameter variation resulted in a wide range of drug perturbation responses for many of the ECC network proteins. For example, Figure 3A displays a clustered subset of monophosphorylated Rb (pRb) time-course drug responses, showing a fairly extreme variety of drug-response dynamics that are possible in response to parameter variation. pRb not only possesses a *quantitative* spectrum of intra-category responses, which is probably expected, but it can also display a range of *qualitative* dynamics, and in fact, can demonstrate all six categories of qualitative drug response dynamics (see *Materials and methods*).

**Figure 3—figure supplement 1.**
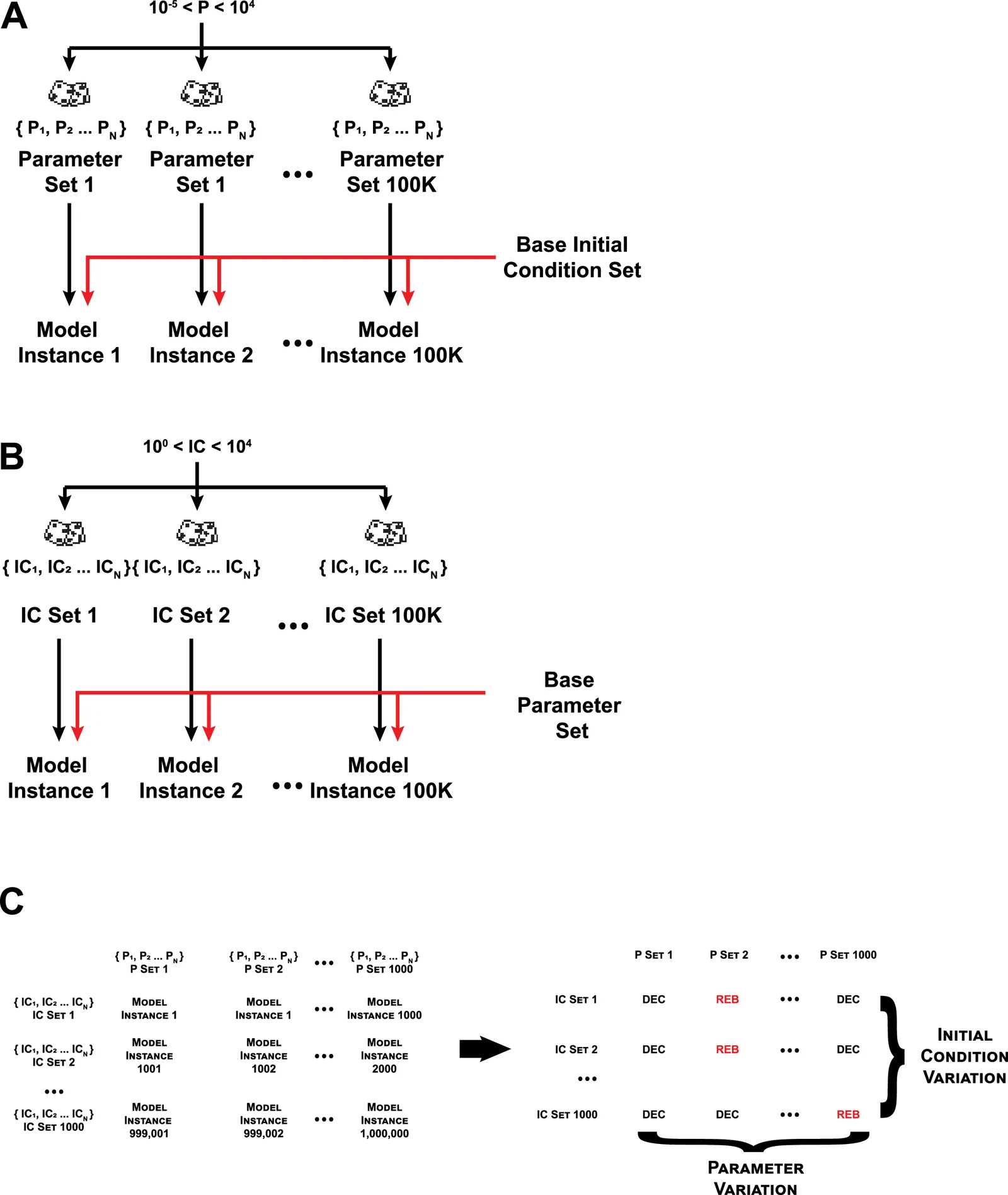
Methods for generating groups of 100,000 unique model instances. (**A**) Model instances are produced by combining a base set of initial conditions (ICs) with a unique set of parameters. Parameter values in each unique parameter set are generated by randomly selecting values for parameters from between the range 10^–5^ and 10^4^. (**B**) Model instances are produced by combining a base parameter set with a unique set of ICs. IC values in each unique IC set are produced by randomly selecting values for ICs between the range 10^0^ and 10^4^. (**C**) Measuring the ability of parameter variation versus IC variation to induce resistance. First, we selected 1000 model instances from the group of model instances produced in (**A**) that demonstrated decreasing dynamics for one of the key output proteins, and 1000 model instances produced in (**B**) that show the same. We then generated 1000 new, unique model instances *per* model instance in group A by taking the parameter set of each group A model instance as the base parameter set and combining it with all of the IC sets from group B, see left side of panel. This produced a total of 1,000,000 unique model instances. We then simulated each of these new model instances and determined the protein dynamic of each of the key output proteins. We then calculated the distribution of observed resistance-associated dynamics for the rows and columns. The distribution of the rows is representative of IC variation and the distribution of the columns is representative of parameter variation.

**Figure 3—figure supplement 2.**
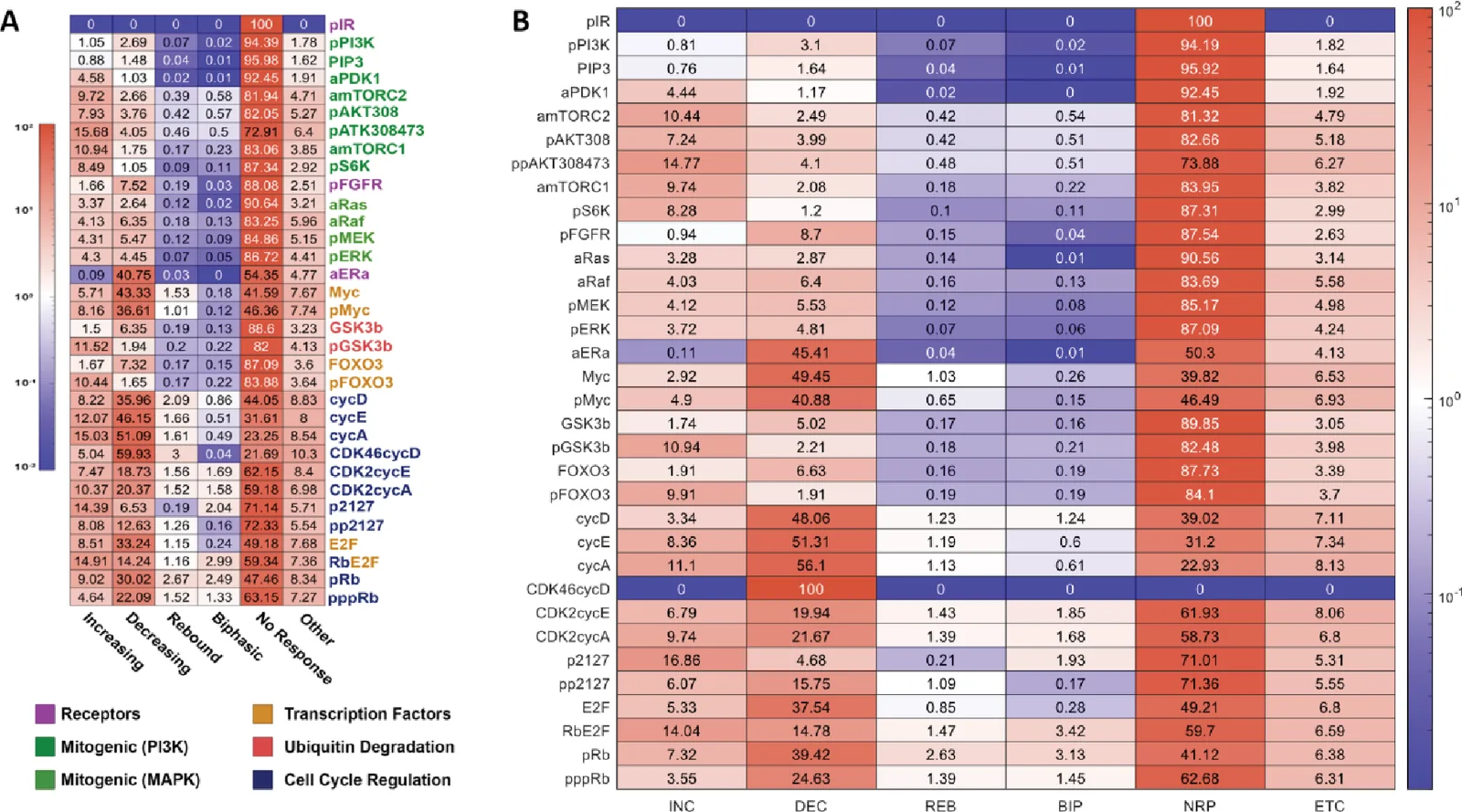
Full repertoire of signalling dynamics identified by MDN, including filtering for strong CDK4/6-Cyclin D suppression. (**A**) Heatmap showing the frequency of each dynamic for a selection of active protein species, across 100,000 model instances (parametric variation), that is the network’s meta-dynamic map. Note that CDK4/6cycD shows a sustained decreasing behaviour in only 59.93% of model instances. (**B**) Heatmap of dynamic frequencies, filtered for model instances that display strong suppression of CDK46cycD. Filtering in this way has very little effect on the distribution of protein dynamics.

**Figure 3—figure supplement 3.**
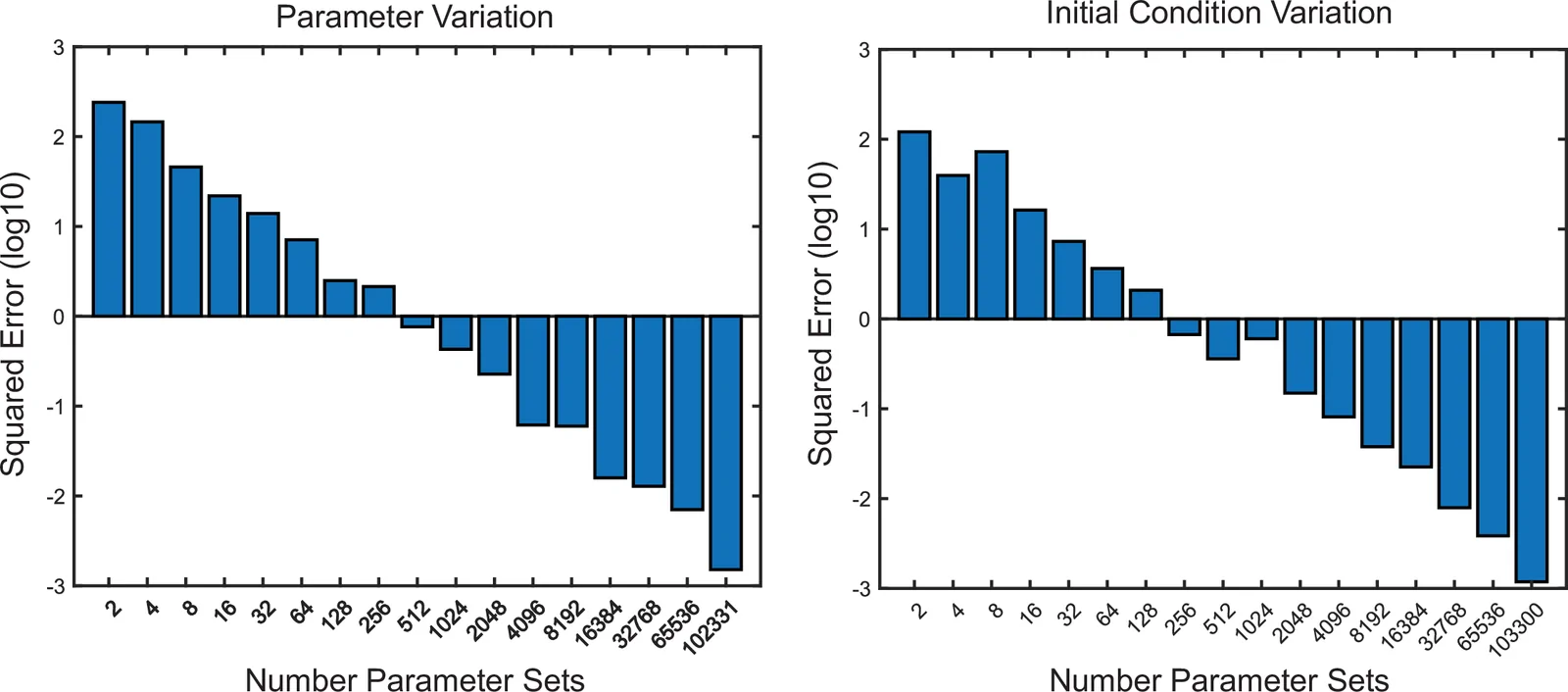
Bar graph showing the total change in the frequency of dynamics when the number of model instances is doubled. Specifically, we calculated the Mean Squared Error between the distributions of protein dynamics for model instance populations of size N and 2 N. Y-axis is the calculated MSE and is in log10 scale, and the X axis shows the size of population N.

Categorising the dynamics of each protein across all of the model instances allowed us to calculate the distribution/frequency of each dynamic for each protein. The distribution of behaviours for the ‘active’ forms of the model’s protein species can be seen in the heatmap in Figure 3B. This overall distribution of protein behaviours is akin to a map that shows how the network state can affect the response of individual proteins to a drug treatment, in this case, the response to simultaneous CDK4/6 and ER inhibition. For many proteins, particularly proteins far upstream of the drug targets, the network state has only a little influence on drug response. This is particularly well demonstrated by the proteins in cluster 1, which show close to 100% no-response (NRP) dynamics.

Other proteins are much more strongly influenced by network state, where under the right conditions, a protein’s response can flip from sensitivity to resistance. The proteins in clusters 4 and 5 show this phenomenon to a moderate degree but also show a predilection towards decreasing dynamics. The predilection to a decreasing dynamic may be due to their proximity to the drug targets CDK4/6 and ER and/or the linearity of the interactions between these proteins and the direct drug targets. It is particularly interesting to observe that most of the major promoters of cell cycle progression downstream of CDK4/6 cluster together (cluster 3, Figure 3B) and are all strongly influenced by the network state. This suggests that heterogeneity can strongly influence whether CDK4/6 inhibition will translate to an effective network-level response and prevent cell cycle progression.

To further assess the capability of the ECC network to facilitate adaptive resistance, we dug into the dynamic distributions of the key output proteins: pRb, pppRb (hyper-phosphorylated Rb), and CDK2cycE. Recall that we have defined resistance dynamics as increasing, biphasic and rebound, and sensitive behaviours as decreasing. While the frequency of sensitive dynamics was much higher than even the sum of resistance dynamics for all three proteins, the ratio of model instances displaying sensitivity to those displaying resistance was much lower than we expected. The ratios of sensitive to resistant behaviours, as percentages of 100,000 model instances, were 30:14, 22:8, and 19:11 (rounded) for pRb, pppRb, and CDK2cycE, respectively (see Figure 3—figure supplement 2A). These results suggest that for every 2 model instances/network states that facilitate sensitivity, there is 1 that facilitates resistance.

We then analysed the model instances further to identify how frequently sensitive and resistant behaviours occurred simultaneously across the three key output proteins. Our results suggest that broad sensitivity across all three output proteins is quite rare, and that resistance is remarkably common (Figure 3C). We observed that only 2.79% of model instances show simultaneous sensitivity across all three output proteins (Figure 3C). In contrast, when evaluating model instances where at least 1 of the output proteins displays a resistance associated dynamic, we observe that this occurs in just over a quarter, 26.04%, of all model instances (Figure 3C).

Given that CDK46cycD is only strongly suppressed in just under 60% of the model instances (Figure 3—figure supplement 2A), we hypothesised that the resistance displayed by the key output proteins may be largely due to insufficient suppression of CDK46cycD. To explore this, we filtered out the model instances that showed strong suppression of CDK46cycD and re-generated the distribution of protein dynamics. The resulting heatmap can be seen in Figure 3—figure supplement 2B. Surprisingly, the distribution of behaviours did not appear to change significantly, with the key output proteins showing very similar levels of adaptive resistance dynamics. This result suggests that the resistance dynamics of the key output proteins are bona fide adaptive resistance mechanisms and not just a result of insufficient suppression of the drug target.

Taken together, these results show that in the face of heterogeneity, the ECC network is robustly capable of facilitating adaptive resistance and that key output proteins display adaptive resistance even when the drug targets are being robustly suppressed.

### Reaction coefficients are a stronger driver of adaptive-resistance dynamics than protein abundance

Having investigated the influence of parametric variation on drug response, we then wanted to explore the effect of IC variation in a similar fashion. To this end, we generated a further 100,000 model instances. This time, however, each model instance possessed an identical ‘nominal’ parameter set (see Supplementary file 5), along with a unique set of ICs. Each unique set of ICs was generated by randomly selecting values between the range 10^0^ and 10^4^, for the inactive form of each protein. See Figure 3—figure supplement 1B for a visual representation of the model generation process. We then repeated the simulation and analysis of protein dynamics as performed previously. The resulting distribution of dynamics can be seen in Figure 4—figure supplement 1A, and the clustering of this distribution can be seen in Figure 4—figure supplement 1B.

Interestingly, we found that compared to parametric variation, IC variation induced much less variety in protein response dynamics. Most proteins demonstrated a singular dominant behaviour and very few proteins exhibited both sensitive and resistance dynamics. This seems to suggest that IC variation is less capable of influencing a protein's drug response. However, we thought that this may have been due to the selected nominal parameter set and may not be a universal phenomenon. To explore this hypothesis, we investigated the ability of parametric variation and IC variation to induce adaptive resistance in our key output proteins. Essentially, we selected sensitive model instances, then randomly varied either parameter values or ICs and measured how frequently the model instance switched from drug-sensitive to drug-resistant.

**Figure 4.**
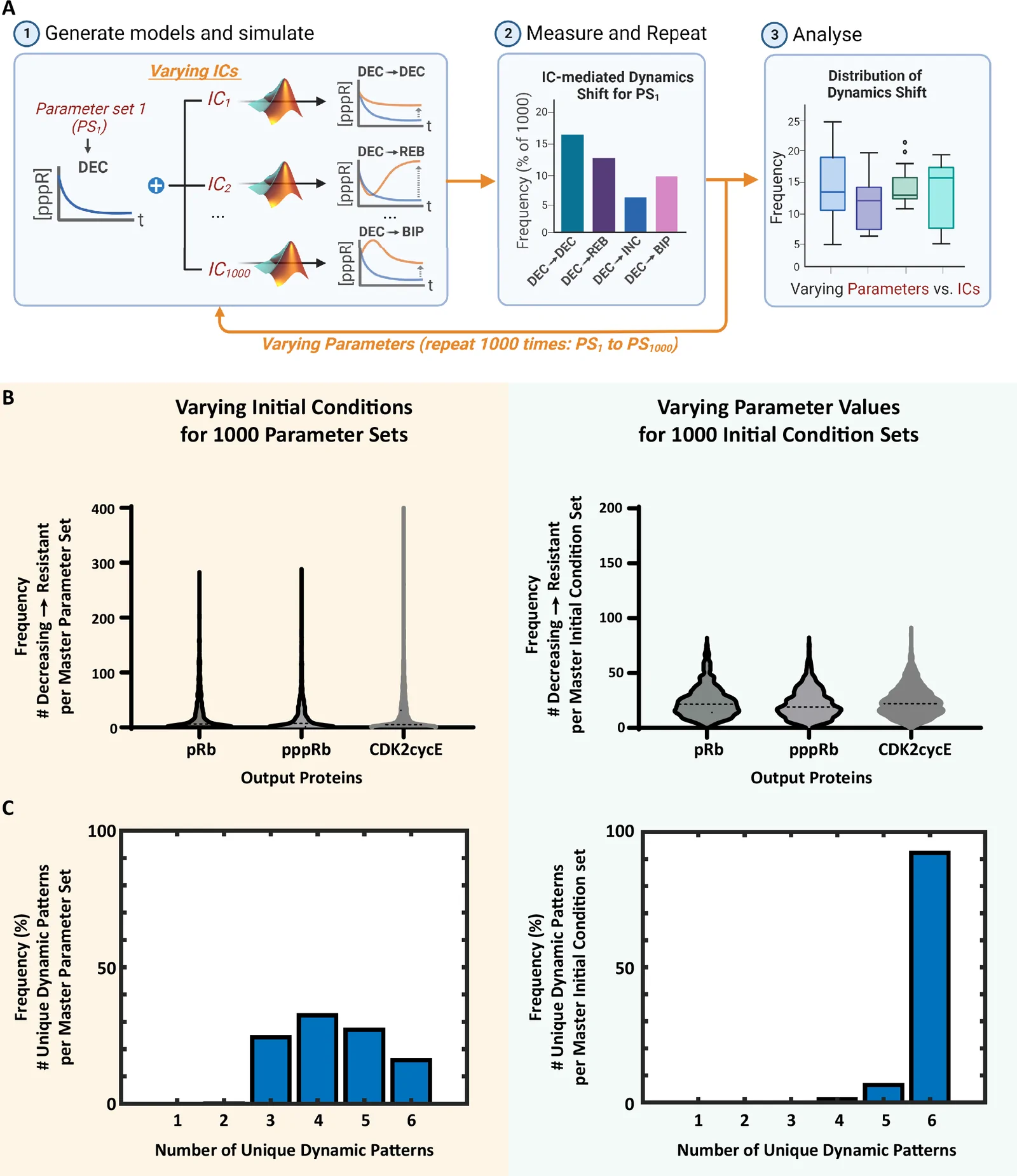
Reaction coefficients are a stronger driver of adaptive-resistance dynamics than protein abundance. (**A**) Overview of the computational pipeline undertaken to compare the effects of parametric variation with initial condition (IC) variation. 1000 unique IC sets are applied to a base parameter set that demonstrates a decreasing dynamic for the key output protein being analysed. Each of the new model instances are simulated and the category of the output protein’s dynamic is determined. Then the number of times the model instances produce a resistance-associated dynamic is measured. This entire process is repeated for 1000 base parameter sets. The final step is measuring the distribution of resistance behaviours when the parameter values are changed and comparing the distribution when the IC values are changed. (**B**) The frequency of which IC variation (left) induces resistance, in each key output protein. Varying IC values usually does not induce resistance, however, for select parameter sets, most ICs produce resistance associated dynamics. This analysis is repeated for parameter variation (right). Contrary to varying IC values, varying parameters usually induces resistance to a small degree, but there are no sets of ICs that are universally resistant. (**C**) The number of unique dynamic categories produced by IC variation (left), versus parametric variation (right). Varying ICs produces a moderate variety of qualitative dynamics, and can produce all 6 dynamic categories, but varying parameters almost always induces every single possible dynamic category for the key output proteins.

To achieve this, we first selected 1000 unique parameter sets and 1000 unique sets of ICs that demonstrated a decreasing dynamic for one of our key output proteins, for example pppRb. Each parameter set was combined with each IC set, resulting in the creation of 1,000,000 unique model instances. These model instances were arranged in a 1000 × 1000 matrix, where the rows represent the unique parameter sets and the columns represent the unique ICs. See Figure 3—figure supplement 1C for a visual representation. Each model instance was simulated, and the dynamic of the key output protein recorded in the matrix. By measuring the frequency of resistant dynamics across the rows and columns, we could compare the ability of parameter variation and IC variation to induce resistance. An overview of this process can be seen in Figure 4A.

**Figure 4—figure supplement 1.**
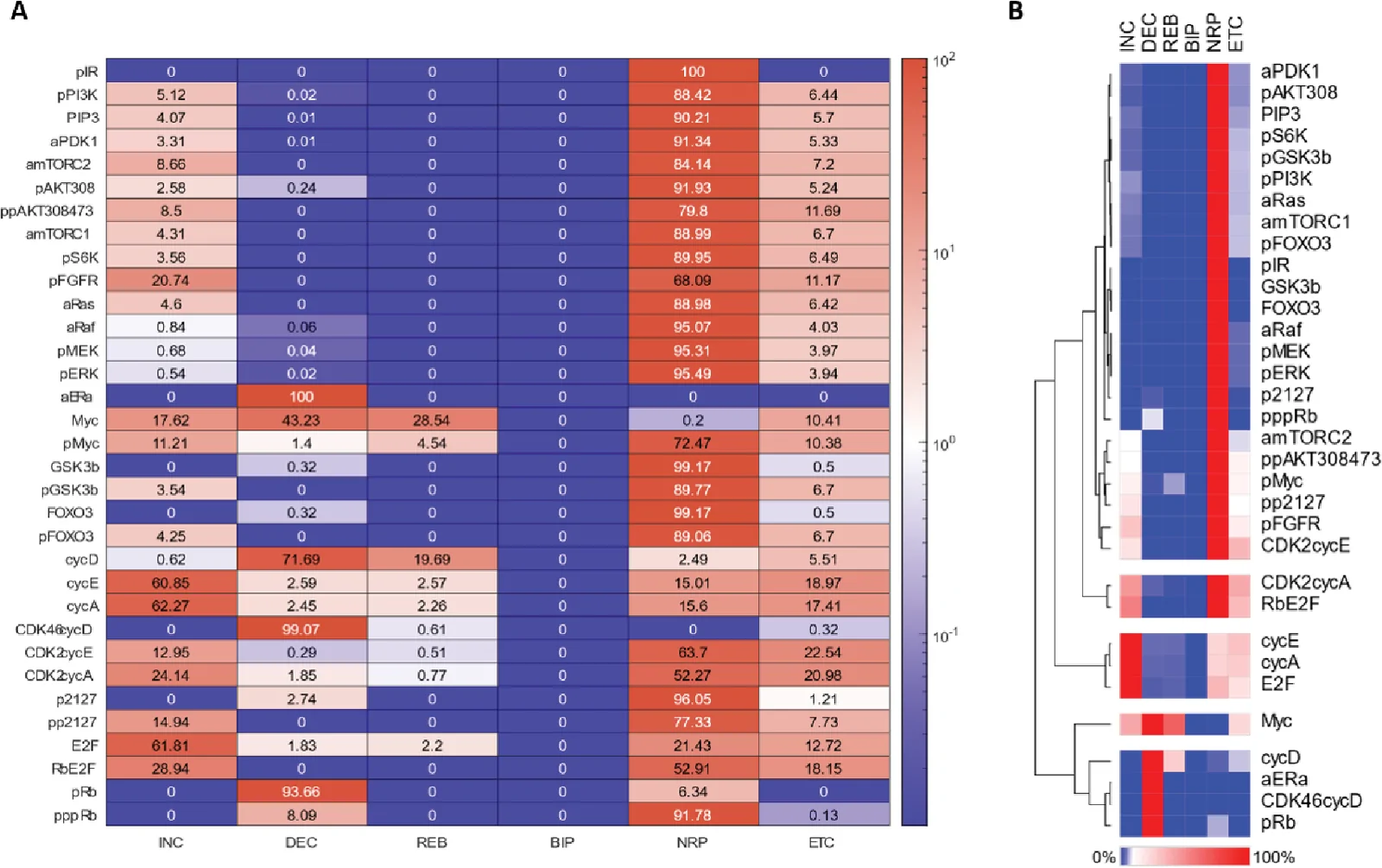
MDN modelling exploring initial condition variation. (**A**) Heatmap showing the frequency of each dynamic for a selection of active protein species, across 100,000 model instances when initial condition values are varied. The base parameter set used was initially randomly selected. Blue represents low frequency and red represents high frequency. (**B**) Clustering of the heatmap in A to highlight proteins with similar distributions of dynamic categories.

**Figure 4—figure supplement 2.**
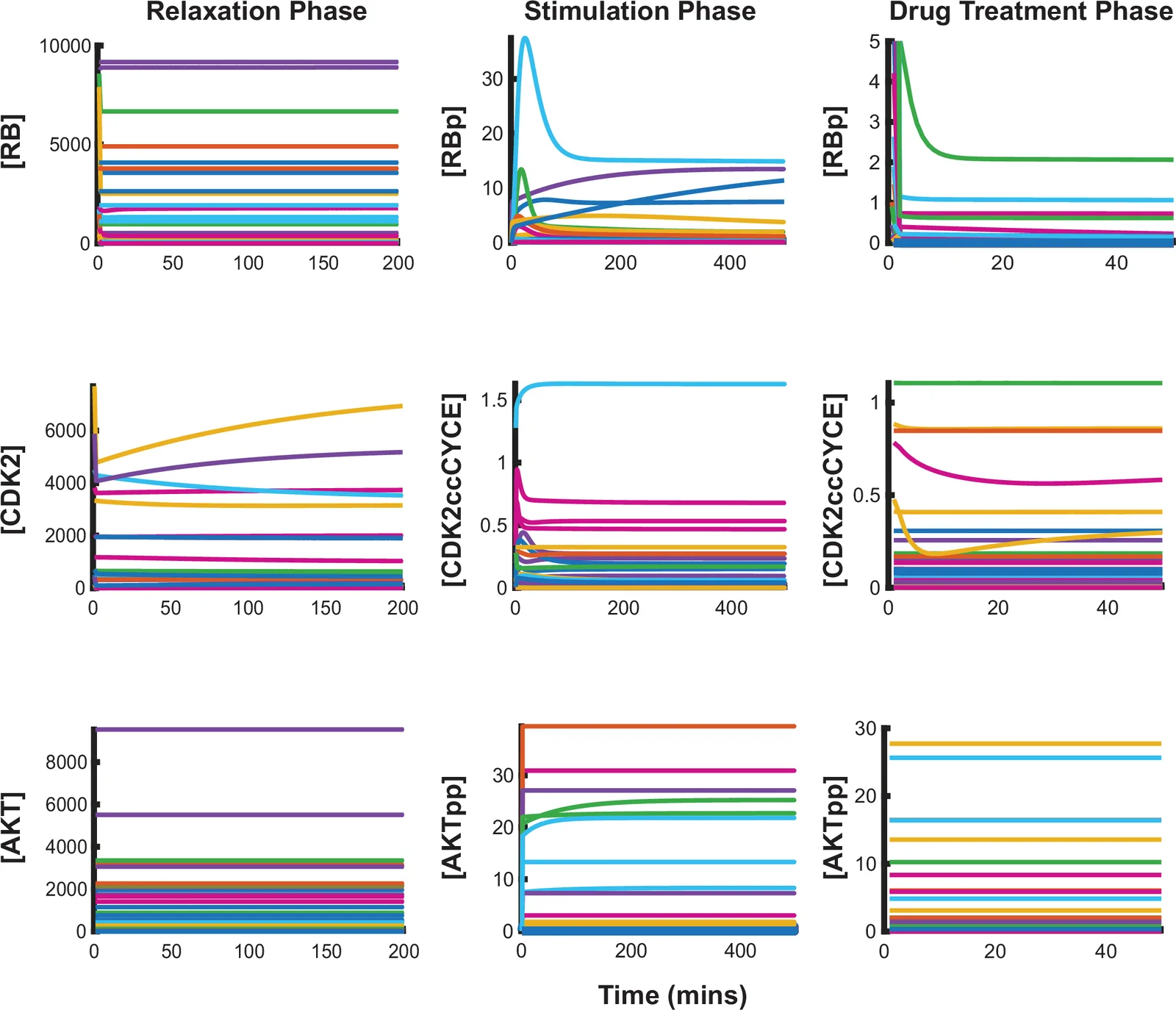
Varying conserved totals shifts the dynamic equilibria of the relevant protein species. 50 different model instances (colours); each with an *identical base parameter set* but different, randomly selected conserved totals for their constituent protein species. Each model instance was simulated as laid out in the methods section of the main text. The first column demonstrates the shifted equilibria with no stimulation, the second under (constant) mitogenic stimulation and the third after drug treatment. Each row is derived from the labelled protein species. Variety can be seen in both steady state equilibria and in the qualitative dynamics before steady state is reached.

We found that for most parameter sets, varying the ICs *never* induced resistance in any of the three output proteins (Figure 4B, left). There was, however, a very small number of parameter sets where altering the ICs frequently induced resistance. In contrast, altering the parameter values *always* induced resistance in a small number of the IC sets, but there were no IC sets wherein parameter variation strongly induced resistance, independent of the protein chosen (Figure 4B, right).

To further characterise the difference between parameter and IC variation, we then investigated the variety of dynamics induced by either parameter or IC variation. For each master parameter or IC set, we measured how many unique dynamic categories were produced across their respective 1000 model instances. We found that varying the ICs still induced a moderate amount of variety in protein dynamics (Figure 4C, left). However, parameter variation almost always produced all six dynamic categories (Figure 4C, right).

These results provide clear evidence that changes in both parameter values and IC values can facilitate the emergence of adaptive resistance. Because conserved totals shift the steady-state values even with fixed kinetics, expression heterogeneity (via totals) can alter pre-treatment equilibria and subsequent drug responses. Figure 4—figure supplement 2 demonstrates this shift. However, changes in parameter values appear to be much more capable of inducing large, qualitative shifts in protein dynamics compared to changes in IC values. The ability of expression variation to induce resistance also seems to be dependent on the master parameter set and there also appears to be no IC set that can induce resistance independent of the parameter values. These results imply that heterogeneity in reaction coefficients is much more likely, and independently able to facilitate the emergence of adaptive resistance compared to heterogeneity in protein abundance. Due to the *independent* ability of parameter variation to *strongly* induce adaptive resistance, we chose to focus on this particular form of heterogeneity in the following studies.

### Reaction coefficients co-ordinate to drive adaptive resistance, independent of their individual strengths

The data produced by our initial MDN modelling pipeline enabled us to identify thousands of model instances where the key output proteins displayed adaptive resistance dynamics. Using these model instances, we next wanted to explore whether there were shared network features driving resistance, or if each model instance was unique in its ability to generate resistance. To explore this in a systematic manner, we extracted subsets of model instances, one for each key output protein and resistance behaviour combination (i.e. protein-dynamic combination) and performed a series of analyses on their parameters.

First, we investigated the mean and SD of each parameter in each protein-dynamic combination to see if there were any individual parameter values underpinning resistance. We found that the mean of most parameters was between 0.1 and 1, but the SD usually spanned 6 orders of magnitude (Figure 5—figure supplement 1). We did observe that all three receptor-activation associated parameters (kc1f1-INSR, kc10f1-FGFR, and kc15f1-ER) were generally much lower in value than the rest, but further investigation ruled that this was generally due to higher values causing the model’s system of ODEs to become too stiff, and therefore unsolvable, and were thus filtered out. We then undertook hierarchical clustering of each protein-dynamic combination’s parameter values to try and identify if there were groups of parameter values driving resistance. However, we were unable to find any meaningful clusters (Figure 5—figure supplement 2). Together, these results led us to conclude that there were no particular individual parameters or groups of parameter values that were responsible for driving resistance.

Having found no patterns in the raw, or absolute, parameter values, we decided to investigate how frequently each parameter contributed to the increase or recovery of protein activity following drug perturbation, for each output protein. To this end, we investigated the effects of parameter knockdowns on the following dynamic features: maximum concentration, final concentration and/or rebound, hereafter collectively referred to as ‘resistance features’. These features were chosen as they represent either an increase or recovery of protein activity, post drug perturbation, that could potentially enable a cell to overcome the drug insult. The relevant resistance features for biphasic and increasing dynamics were maximum concentration and final concentration, and the relevant features for rebound dynamics were rebound and final concentration. Rebound is not applicable to biphasic and increasing dynamics as it simply hasn’t occurred, and the maximum concentration is only applicable to rebounding dynamics if the rebound overshoots the initial concentration, which is then covered by final concentration.

To measure the influence of parameters on the resistance features of the key output proteins, we performed a high-throughput perturbation analysis wherein we reduced the value of each parameter by 20% in the third simulation phase, that is the drug treatment phase. Parameters were perturbed one parameter at a time, and we measured the effect on the output protein’s resistance features. A breakdown of the measured dynamic features, including the resistance features just described, is given in Figure 5—figure supplement 3.

**Figure 5.**
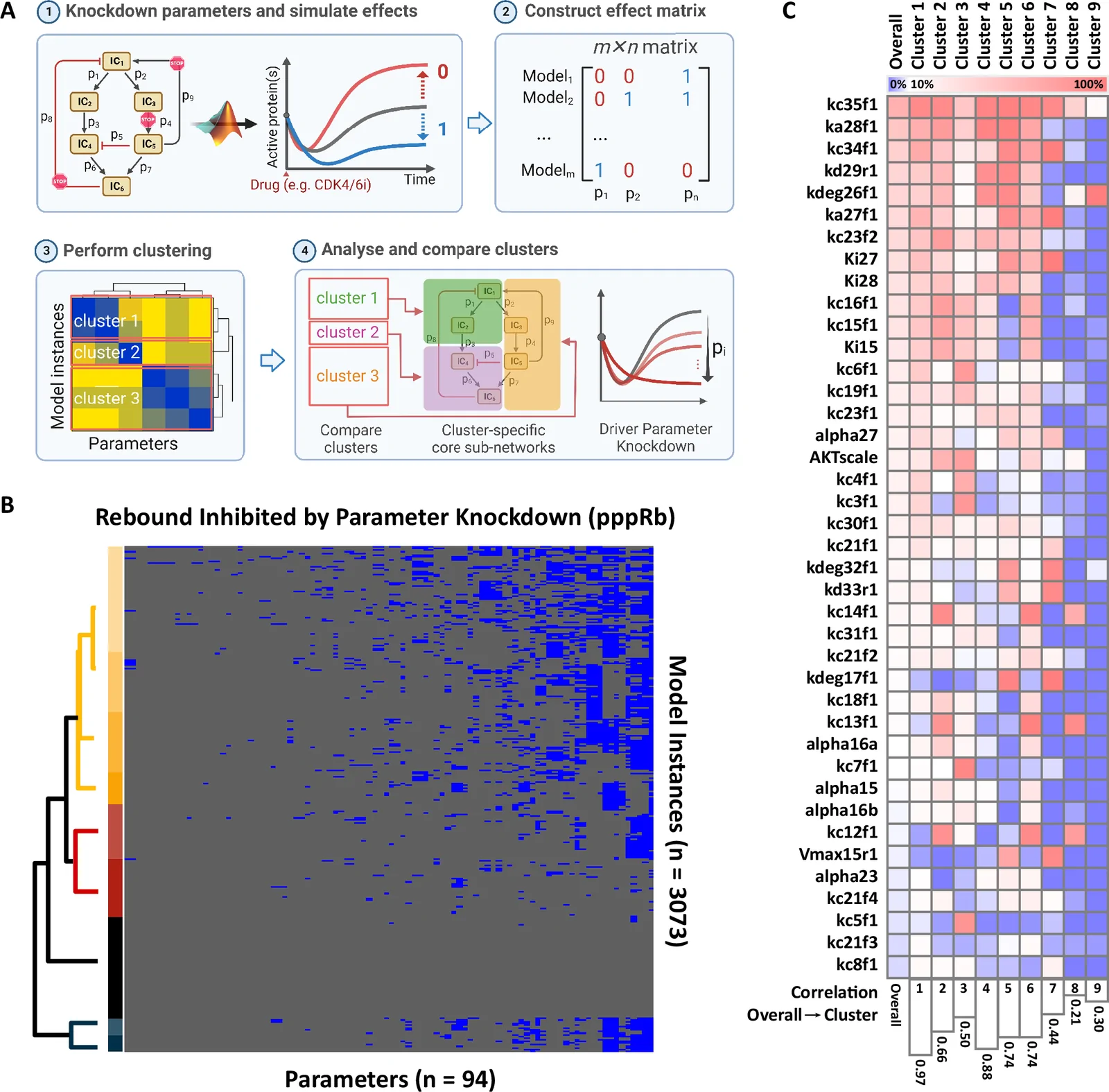
MDN modelling identifies core sub-networks that facilitate resistance. (**A**) Overview of the process undertaken to investigate the relationship between parameter knockdown and resistance-dynamic features to identify resistance driving parameter signatures. Each parameter is knocked down by 20%, one at a time, and the effect on the protein dynamics is measured. If the knockdown decreases resistance it scores a 1, otherwise it scores a 0. This process is repeated for each parameter, one at a time, for each model instance. The end result is a matrix where the columns represent parameters, and the rows represent the model instances. This matrix can then be analysed using clustering methods to identify parametric resistance signatures. (**B**) Heatmap produced by hierarchical clustering of the parameters that contribute to rebounding pppRb, for model instances that display rebounding pppRb. (**C**) Comparing parametric resistance signatures with the parameters that most frequently contribute to resistance overall. Parameters are ranked by how frequently they contribute to rebounding pppRb. Clusters are aligned with overall ranking. The lower bar graph represents the correlation between the overall ranking and each cluster specific ranking. This heatmap shows that some parametric resistance signatures align closely with the overall ranking, but some clusters are quite different, emphasising the context specificity of resistance.

If the knockdown of a parameter resulted in a reduction in the resistance features of the output proteins’ dynamic, it scored a 1; otherwise it scored a 0. Repeating this process for each parameter and model instance resulted in an M x N matrix, where M is the number of model instances (protein and dynamic dependent) and N is the number of perturbed parameters (Chandarlapaty, 2012), for each protein-dynamic combination. See Figure 5A for a visual representation of this and the remaining overall process.

**Figure 5—figure supplement 1.**
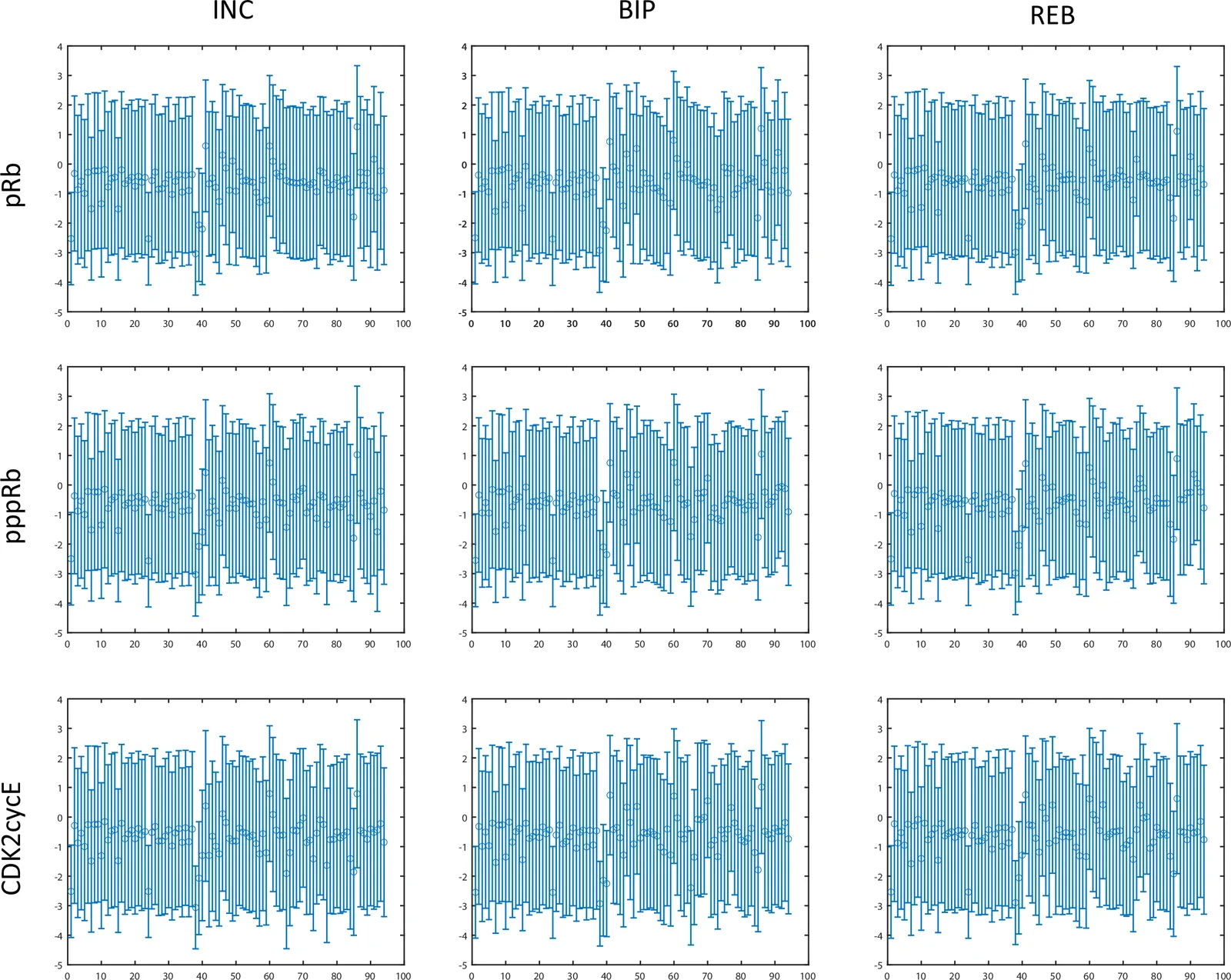
Mean and SD of the absolute log10 transformed parameter values for each protein-dynamic combination. Y-axis represents the range of possible log10 parameter values, the X-axis represents each individual parameter (in the order they appear in the IQM reaction file).

**Figure 5—figure supplement 2.**
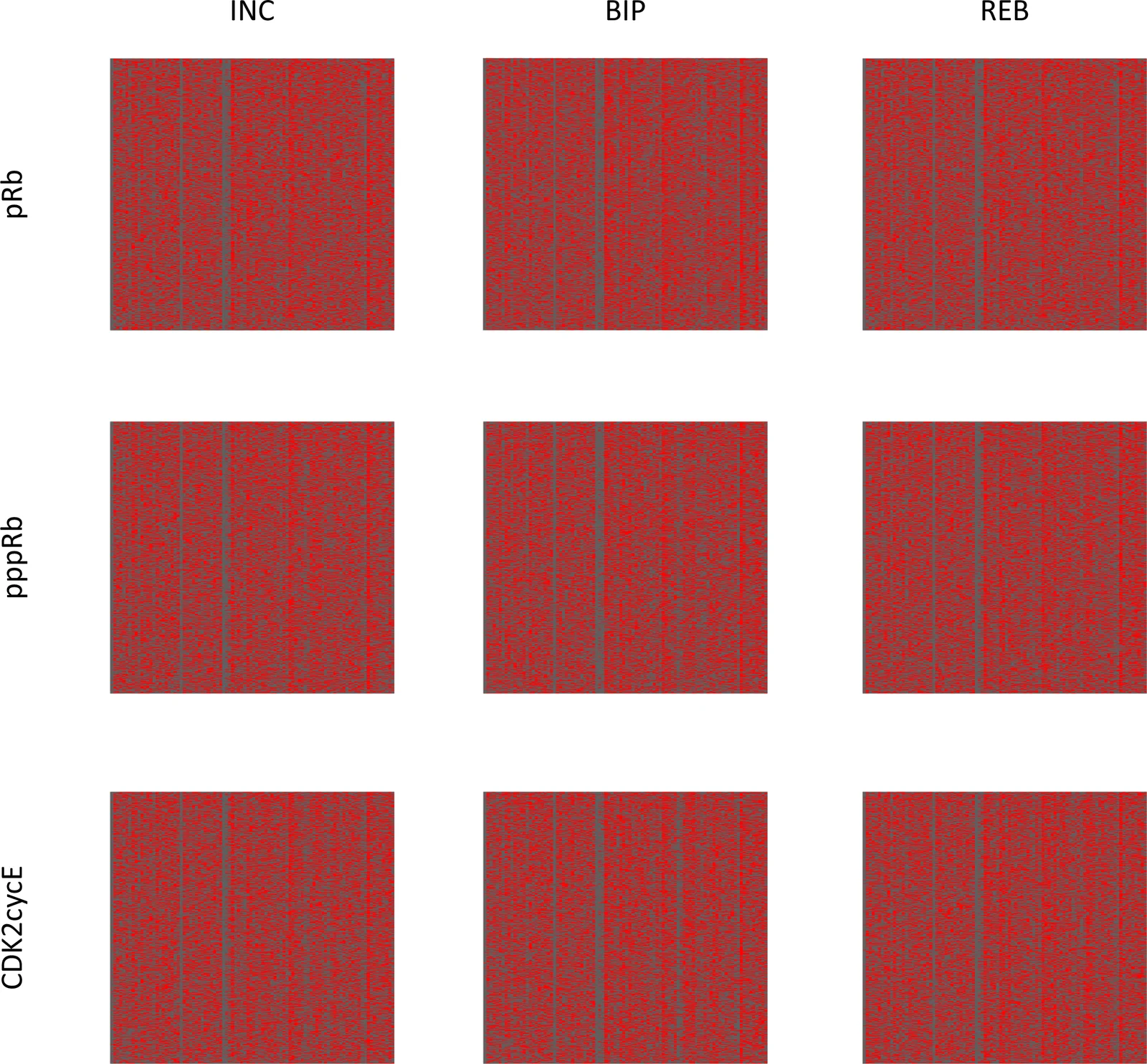
Patterns in the absolute values of parameters driving resistance associated dynamics for key output proteins. Heatmaps showing the results of the hierarchical clustering of the raw parameter values for each protein-dynamic combination. Very little clustering can be observed, suggesting that there are no patterns in the absolute values of parameters with respect to resistance associated dynamics.

**Figure 5—figure supplement 3.**
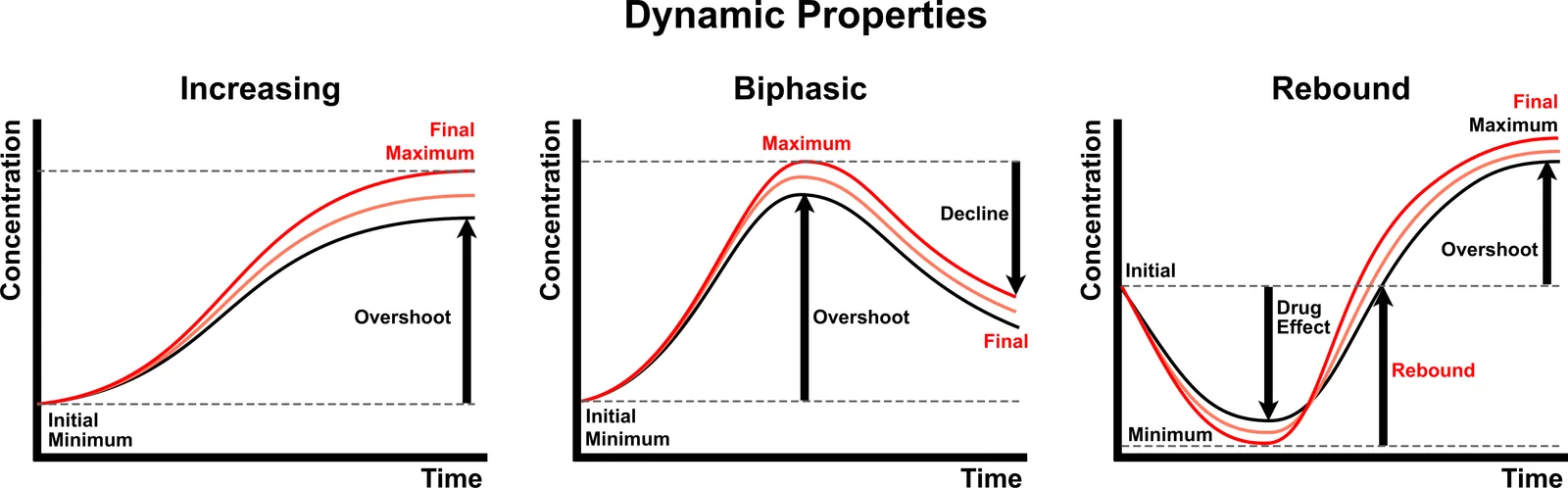
Breakdown of the various measurable features of a protein’s dynamic response following drug perturbation. Resistance features (red) are a subset of these dynamic features and are defined by the recovery or increase in the activity of a protein following drug perturbation, as represented by the increase in concentration of the active form of the protein. The transition from black to red lines represent an increase in the resistance being displayed.

**Figure 5—figure supplement 4.**
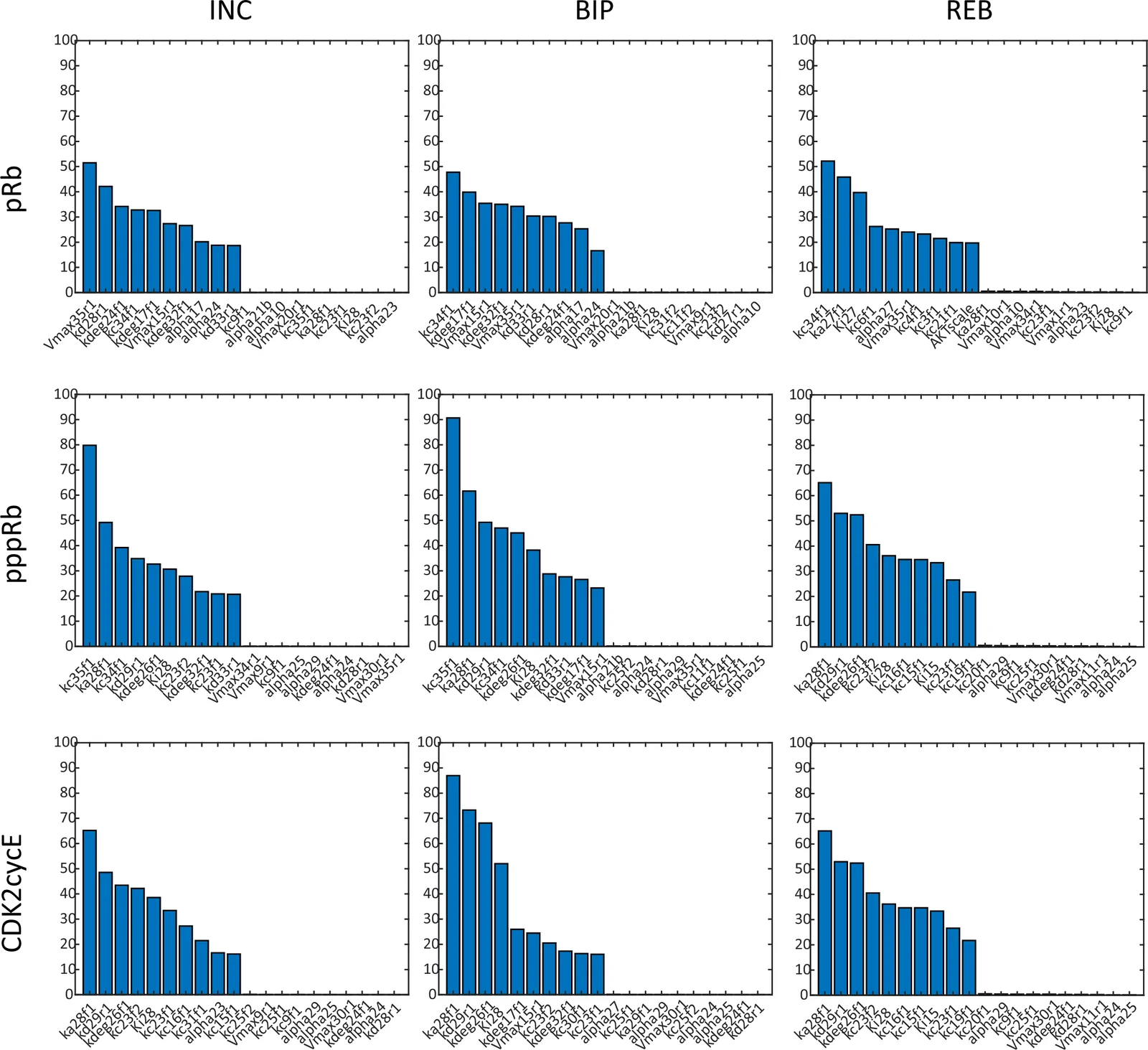
Top and bottom 10 ranked parameters for each protein-dynamic combination. Ranking is based on the overall contribution of each parameter to the resistance features for dynamic of each protein-dynamic combination. For biphasic and increasing dynamics, the ability of each parameter to contribute to the maximum concentration and final concentration was measured, and for the rebounding dynamics, the contribution to rebound and final concentration was measured. The y-axis refers to the percentage of the model instances in which the parameters along the x-axis contribute to the relevant resistance features, for that particular protein dynamic-output protein category.

**Figure 5—figure supplement 5.**
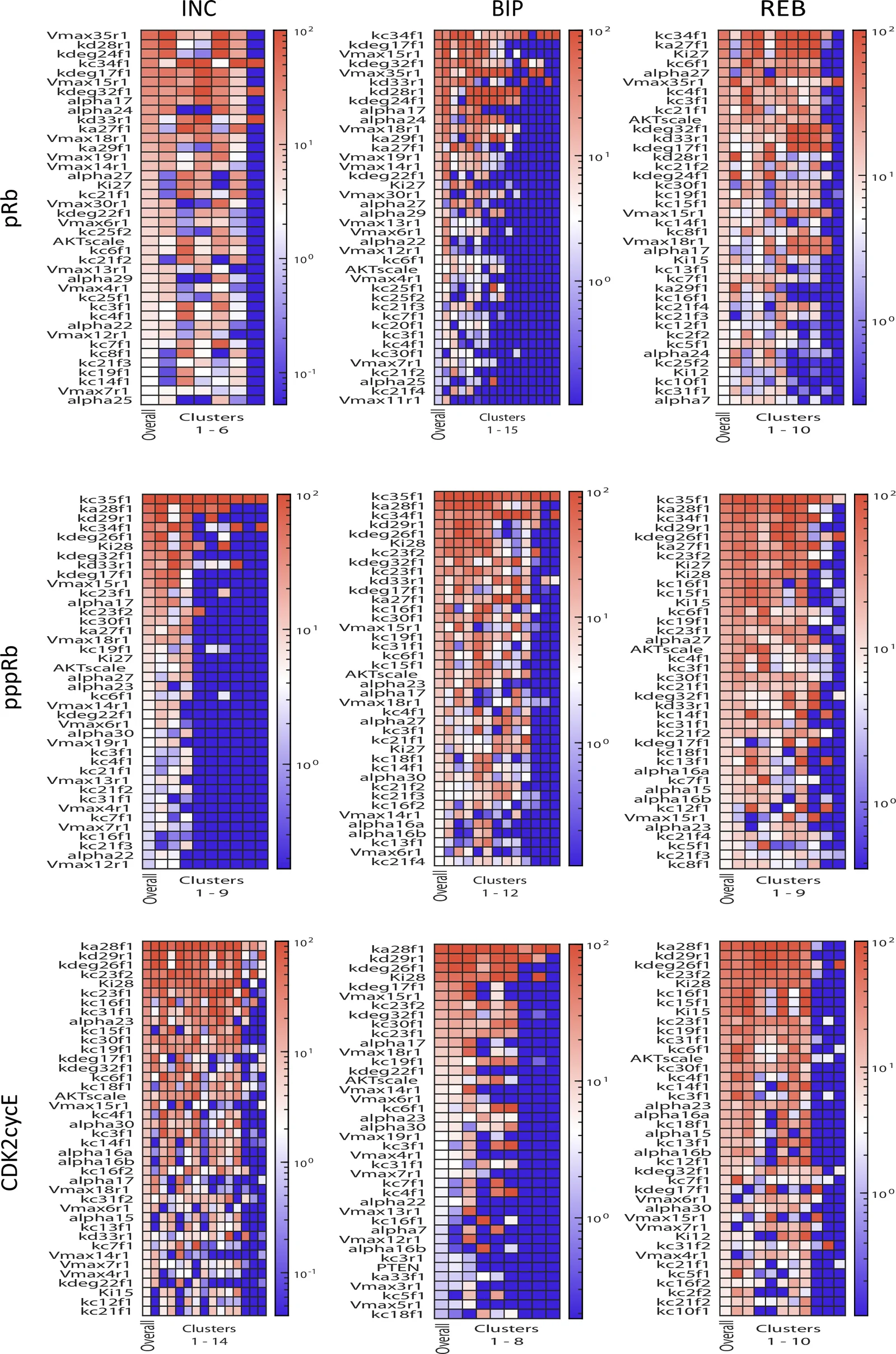
Heatmaps comparing the overall ranking of each protein-dynamic combination to the cluster-specific rankings. Rank is determined by how frequently each parameter contributes to the resistance features of the dynamic of each protein-dynamic combination.

Initially, we wanted to determine how frequently each parameter contributed to the resistance features of each output protein. To achieve this, we summed up the number of times a parameter scored a 1 in the preceding analysis across all model instances, for each protein-dynamic combination. The results were plotted as bar graphs (top and bottom 10 parameters), which can be seen in Figure 5—figure supplement 4. The graphs showed clear rankings in how frequently each parameter contributed to the adaptive resistance features of each protein dynamic. Each protein-dynamic combination displayed a unique ranking but there also appeared to be a significant overlap in the top-scoring parameters. There also appeared to be more intra-protein overlap than inter-protein overlap, and pppRb and CDK2cycE shared much more similarity with each other than either with pRb. It was also interesting to observe that for biphasic pppRb and CDK2cycE, their top-scoring parameters accounted for approximately 90% of all model instances, indicating that these parameters must be critical in driving this particular dynamic for these proteins. The remaining top scoring parameters for each protein-dynamic subset only accounted for between 50 and 80% of model instances, suggesting each protein-dynamic combination possesses a degree of heterogeneity in the parameters that drive their adaptive resistance dynamics.

Together, the above results show that it is not the value of individual parameters that drive adaptive resistance, but rather it is the manner in which parameters co-ordinate to shape the quantitative and qualitative features of a protein’s dynamic that drives adaptive resistance.

### MDN modelling can identify a full spectrum of core sub-networks that facilitate resistance

The observation that the top-scoring parameters generally do not broadly drive adaptive resistance dynamics suggests that there may be a number of different network states that are capable of driving a given resistance dynamic. To investigate this possibility, we subjected the matrices produced in the previous analysis to hierarchical clustering to try and find groups of model instances with shared resistance-driving parameter signatures. As a prime example, we first focused on rebounding hyperphosphorylation of Rb; both as the hyperphosphorylation of Rb strongly promotes cell cycle progression, and because previous studies have demonstrated the role of this protein-dynamic combination in CDK4/6 inhibitor resistance (Herrera-Abreu et al., 2016; Kim et al., 2022; Narasimha et al., 2014). Figure 5B displays a heatmap showing how rebounding pppRb model instances cluster, with respect to the contribution of their parameters to the rebounding dynamic.

The clustering of the pppRb-rebound model instances produced nine robust clusters, where robust clusters are defined as those that contain at least 1% of the total protein-dynamic combination model instances, and the top-scoring parameter in the cluster accounts for at least 80% of its constituent model instances. Simply put, the clusters accounted for a high enough proportion of the model instances and the top scoring parameter was robustly representative of the model instances within the cluster. The left-most column of the heatmap in Figure 5C shows the overall ranking for how frequently each parameter contributes to the rebound dynamic of pppRb, and the remaining nine columns represent the nine clusters, aligned with the overall ranking. We observed a fairly strong consensus amongst the top-scoring parameters, with the majority of the cluster differences coming from the middle and lower parameters (Figure 5C). Of note, this pattern appeared to be broadly true when we analysed all the nine protein-dynamic groups (Figure 5—figure supplement 5).

**Figure 6.**
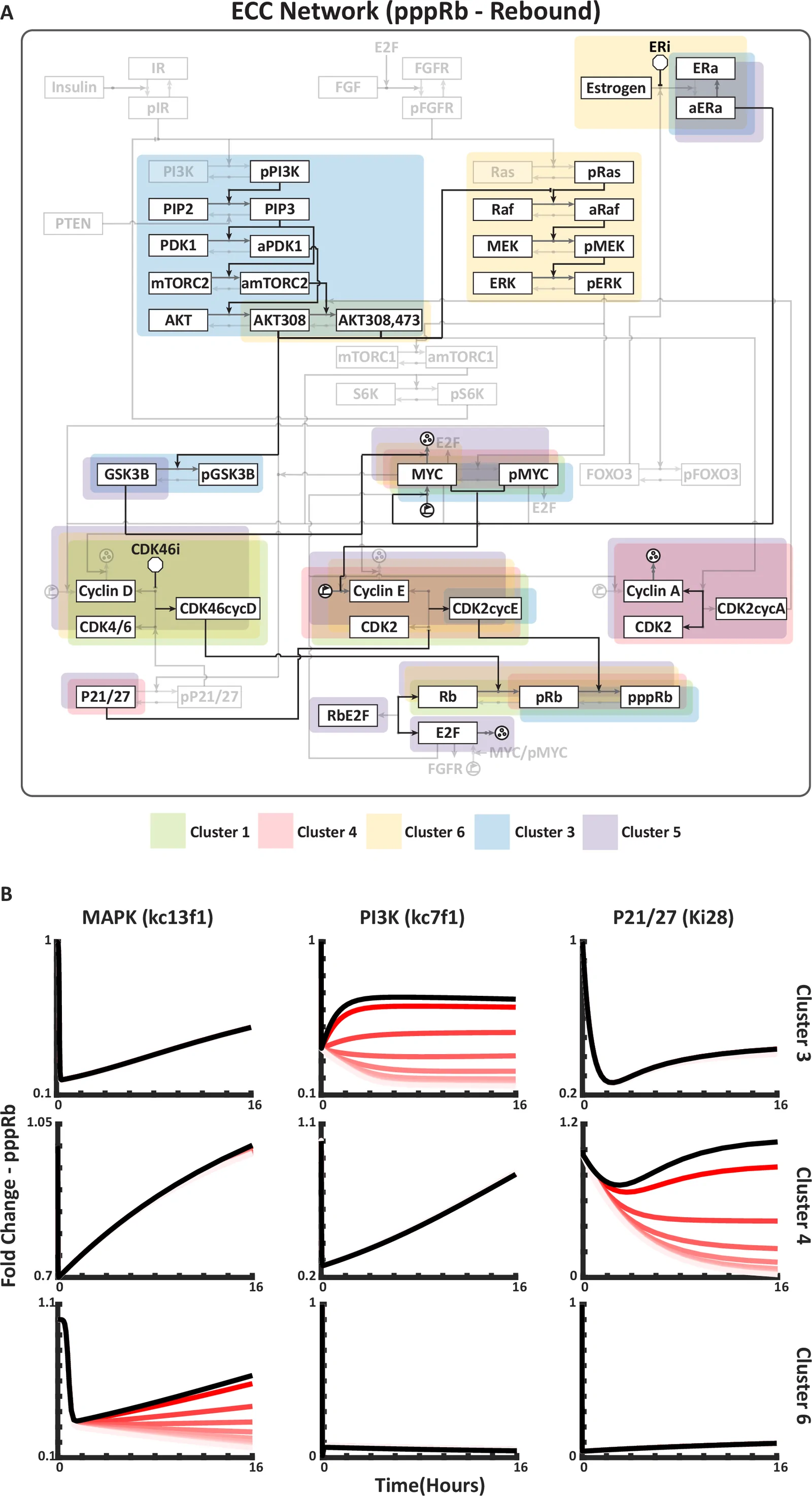
Identification of subnetworks driving pppRb rebound-mediated resistance within the ECC network. (**A**) Cluster-specific parameter signatures overlaid with the ECC network to highlight subnetworks that drive resistance through rebound of pppRb. Only the five largest clusters (out of nine) are displayed. (**B**) Parameter knockdowns for the top-scoring parameters in three select clusters that display divergent resistance driving parameter signatures. Black represents treatment with CDK4/6 and ER inhibitors alone, decreasingly-red lines represent the addition of an increasingly potent parameter knockdown. These results highlight the context specificity of parameter knockdown when attempting to prevent adaptive resistance.

Despite broad similarities between the clusters, many of the individual clusters did not correlate particularly well with the overall ranking and possessed clear differences in their resistance-driving parameter signatures (Figure 5C). These results suggest that the same drug response dynamic for the same protein in two different cells can be driven by different network states. To highlight the similarities and differences between the subnetworks underpinning resistance driven by pppRb rebound, we overlaid the parameter signatures of the five largest clusters on the ECC network schematic (Figure 6A). This revealed significant overlap between the clusters around the formation of the CDK2-cyclin E complex and the hyperphosphorylation of Rb itself. These results were to be expected as the CDK2-cyclin E complex is directly responsible for the hyperphosphorylation of Rb and confirms the logical hypothesis that CDK2 activation can drive the reactivation of Rb phosphorylation following CDK4/6 and ER inhibition. However, that almost all the clusters shared this core network suggests that this subnetwork alone is likely sufficient to enable the reactivation of Rb phosphorylation on its own and would be strongly selected for by cancer during treatment. Similarly, but less expectedly, we also found strong overlap at the level of Myc (Figure 6A), suggesting a strong likelihood for its involvement in the development of resistance.

**Figure 6—figure supplement 1.**
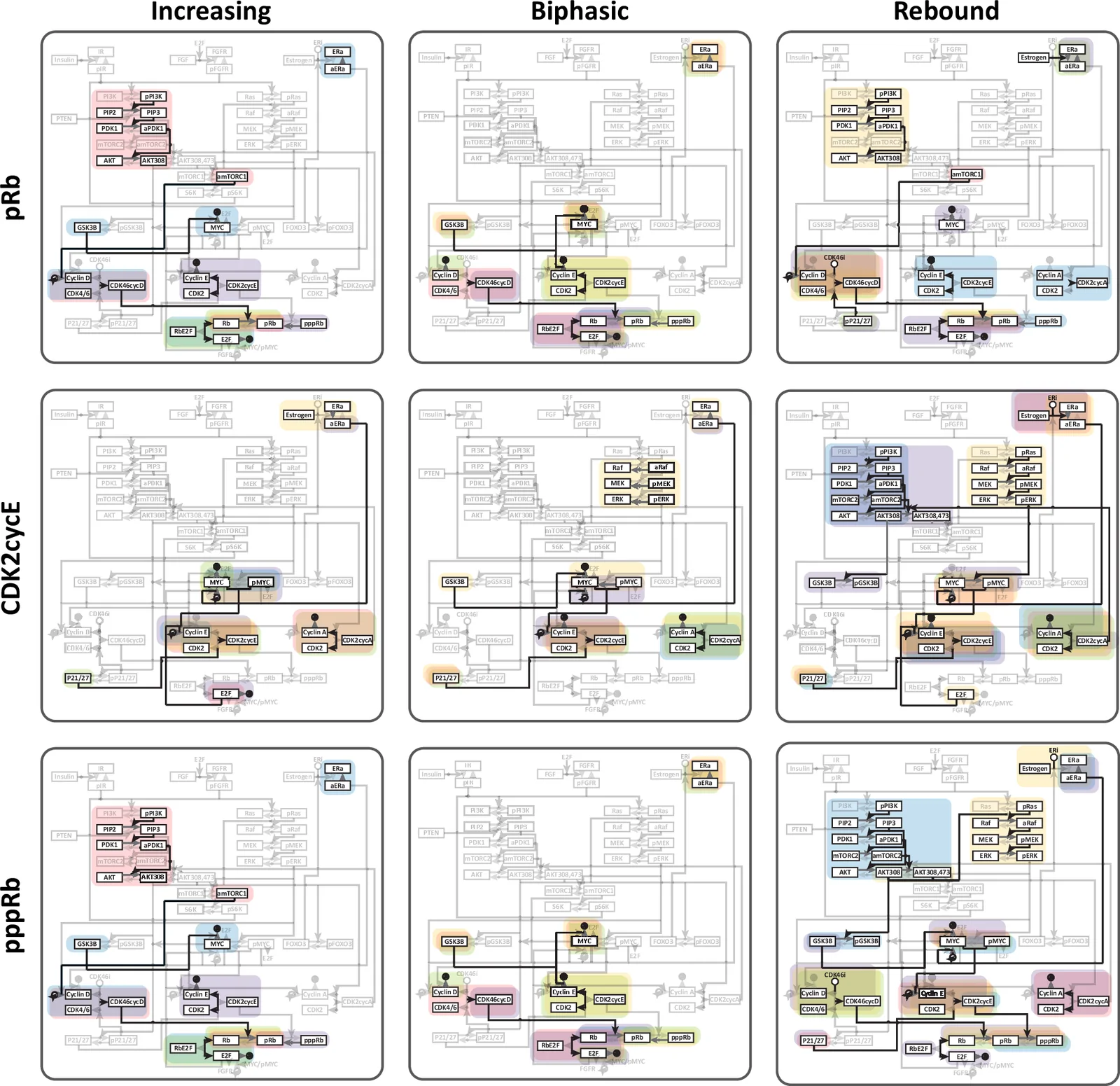
Parameter-signature ECC network overlays for each of the nine protein-dynamic combinations, presented side-by-side for easier comparison of protein-dynamic combinations. Note that Figure 5—figure supplement 5A-I displays the results for the individual protein-dynamic combination in more detail.

**Figure 6—figure supplement 2.**
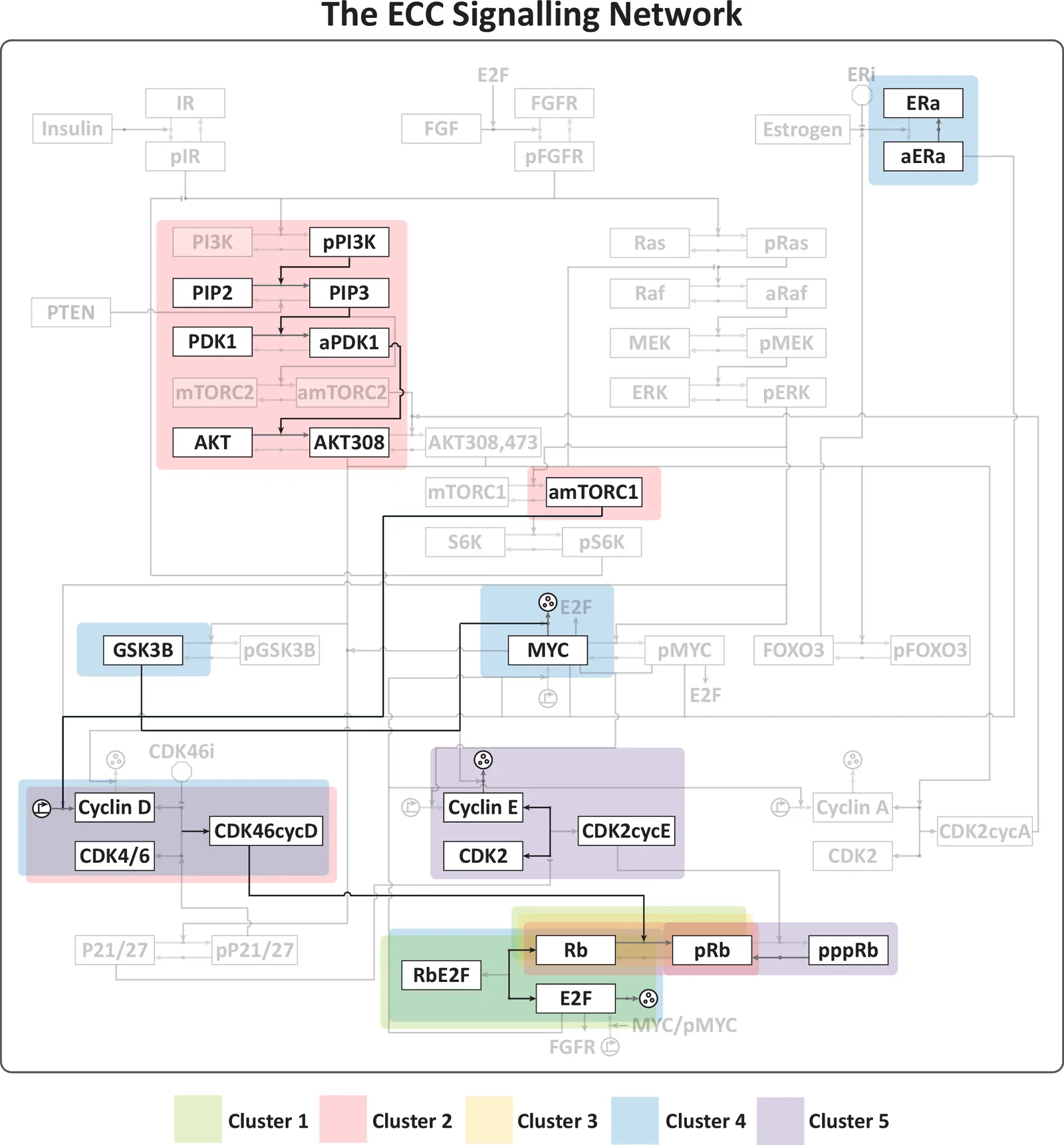
Cluster-specific parameter signatures with the ECC network, shown here for the protein-dynamic pRb-INC combination. Each figure (Figure 5—figure supplement 5A-I) is the overlay of the respective cluster-specific parameter signatures with the ECC network, for each protein-dynamic combination. Each figure displays the five largest clusters for each protein-dynamic combination.

**Figure 6—figure supplement 3.**
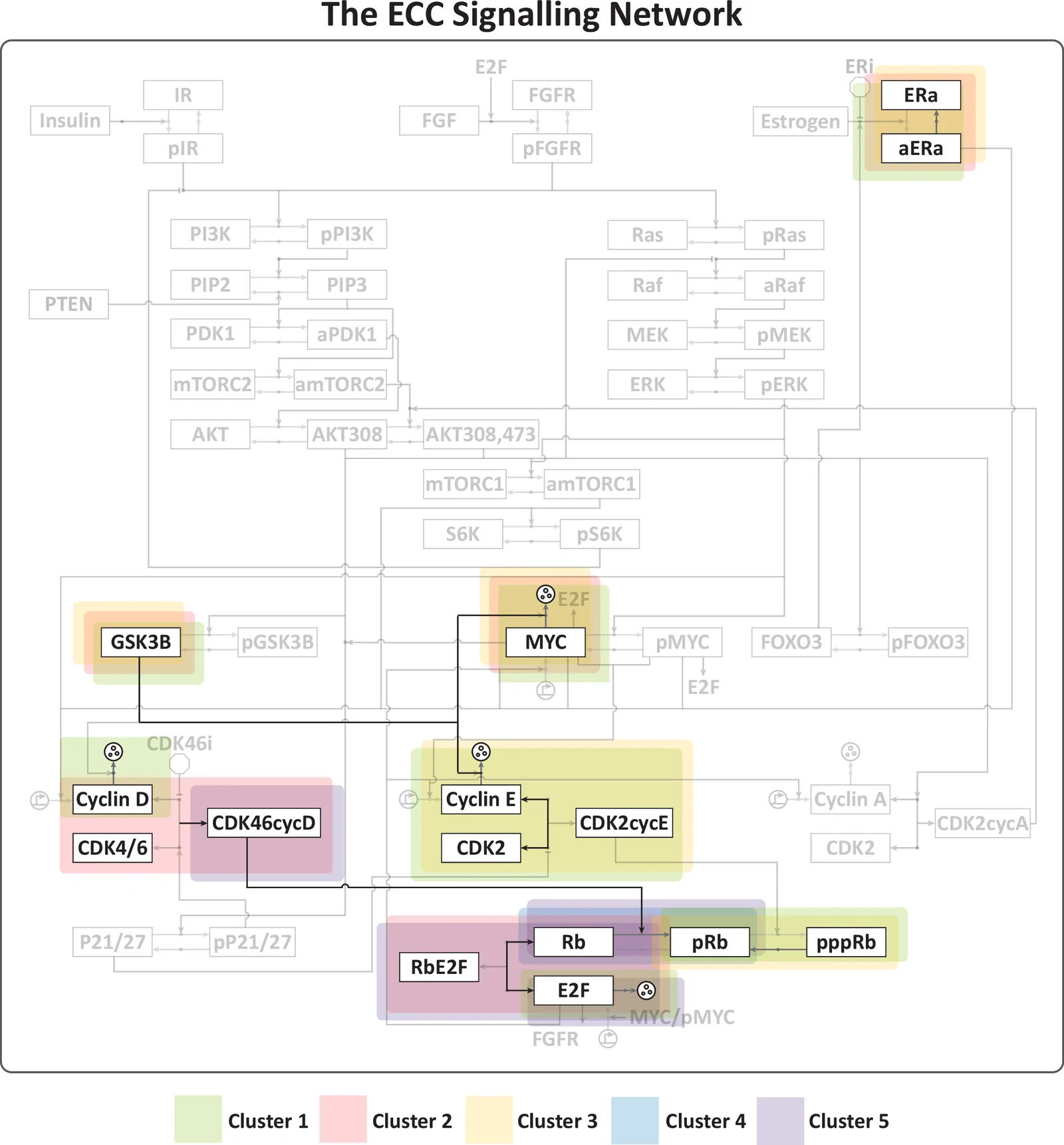
Cluster-specific parameter signatures with the ECC network, shown here for the protein-dynamic pRb-BIP combination. Each figure (Figure 5—figure supplement 5A-I) is the overlay of the respective cluster-specific parameter signatures with the ECC network, for each protein-dynamic combination. Each figure displays the five largest clusters for each protein-dynamic combination.

**Figure 6—figure supplement 4.**
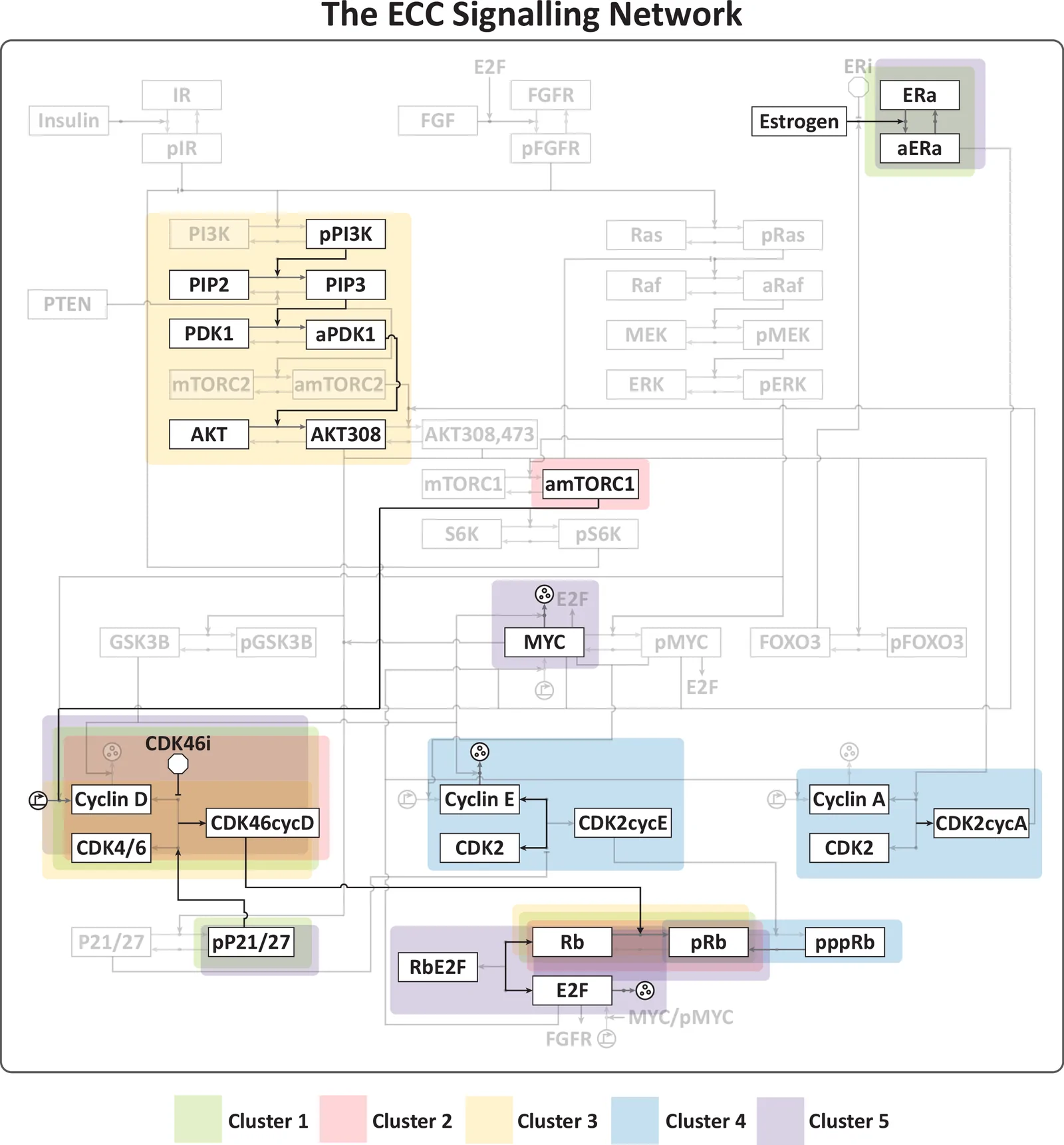
Cluster-specific parameter signatures of the ECC network, shown here for the protein-dynamic pRb-REB combination. Each figure (Figure 5—figure supplement 5A-I) is the overlay of the respective cluster-specific parameter signatures with the ECC network, for each protein-dynamic combination. Each figure displays the five largest clusters for each protein-dynamic combination.

**Figure 6—figure supplement 5.**
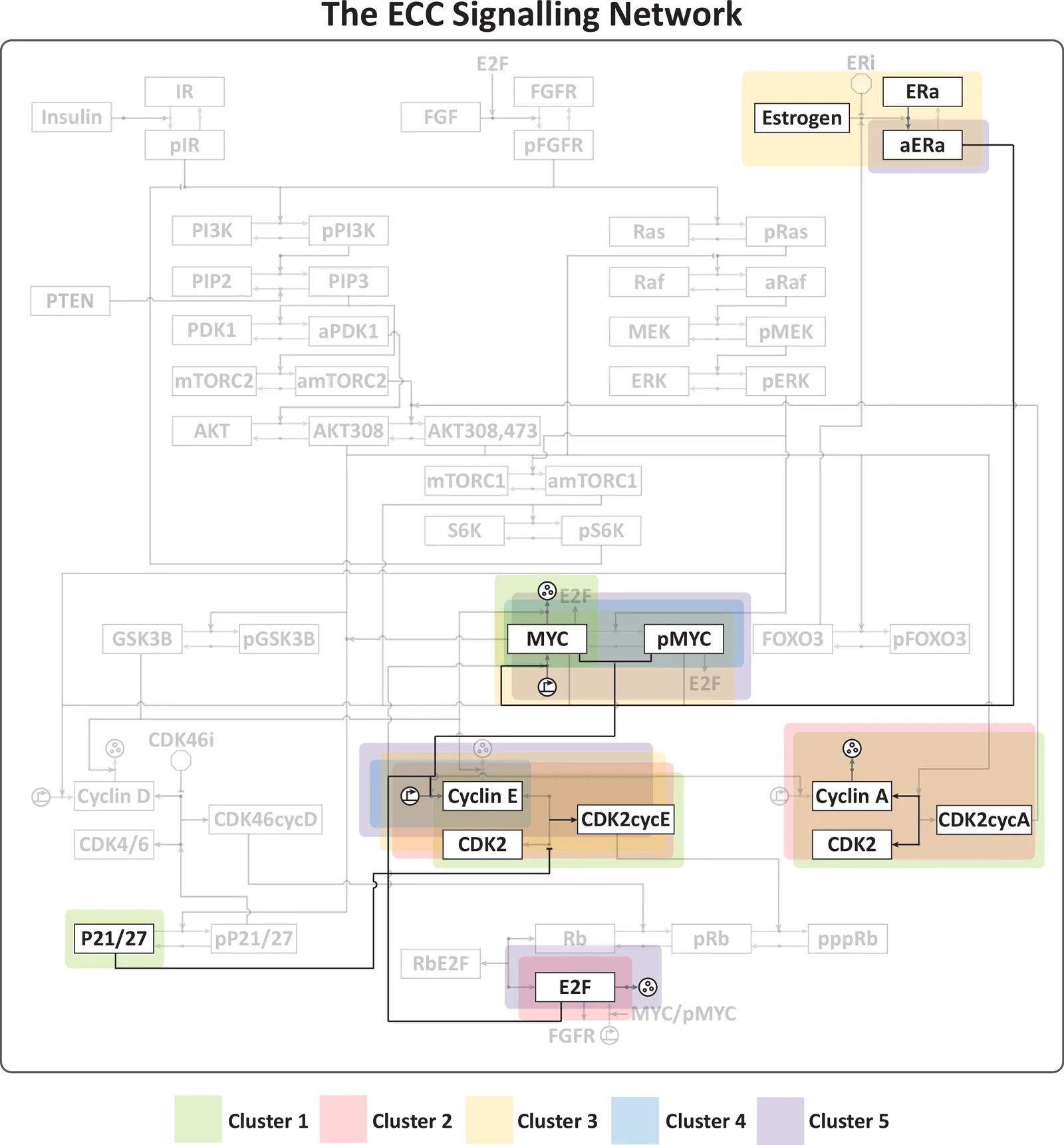
Cluster-specific parameter signatures with the ECC network, shown here for the protein-dynamic CDK2cycE-INC combination. Each figure (Figure 5—figure supplement 5A-I) is the overlay of the respective cluster-specific parameter signatures with the ECC network, for each protein-dynamic combination. Each figure displays the five largest clusters for each protein-dynamic combination.

**Figure 6—figure supplement 6.**
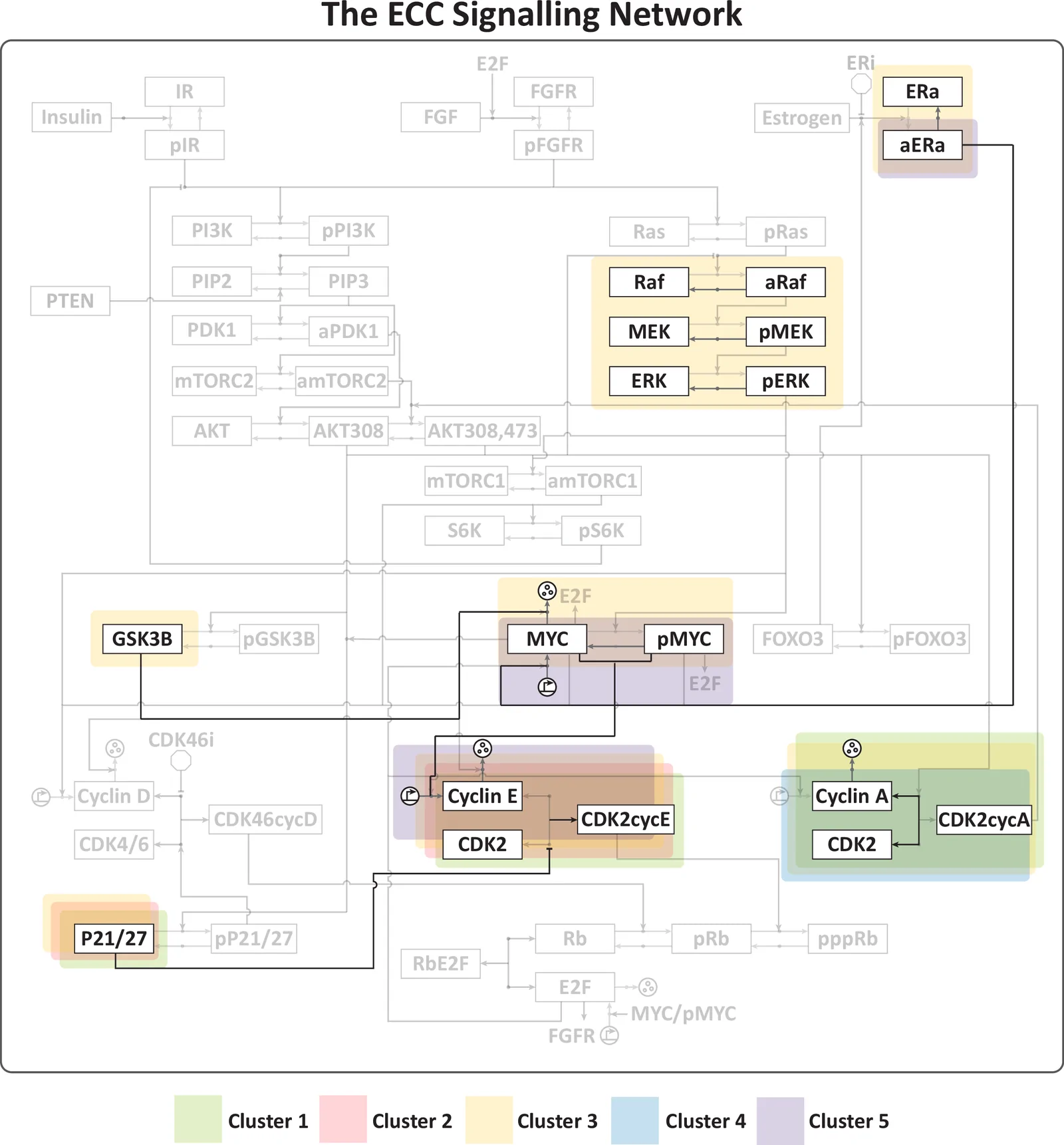
Cluster-specific parameter signatures with the ECC network, shown here for the protein-dynamic CDK2cycE-BIP combination. Each figure (Figure 5—figure supplement 5A-I) is the overlay of the respective cluster-specific parameter signatures with the ECC network, for each protein-dynamic combination. Each figure displays the five largest clusters for each protein-dynamic combination.

**Figure 6—figure supplement 7.**
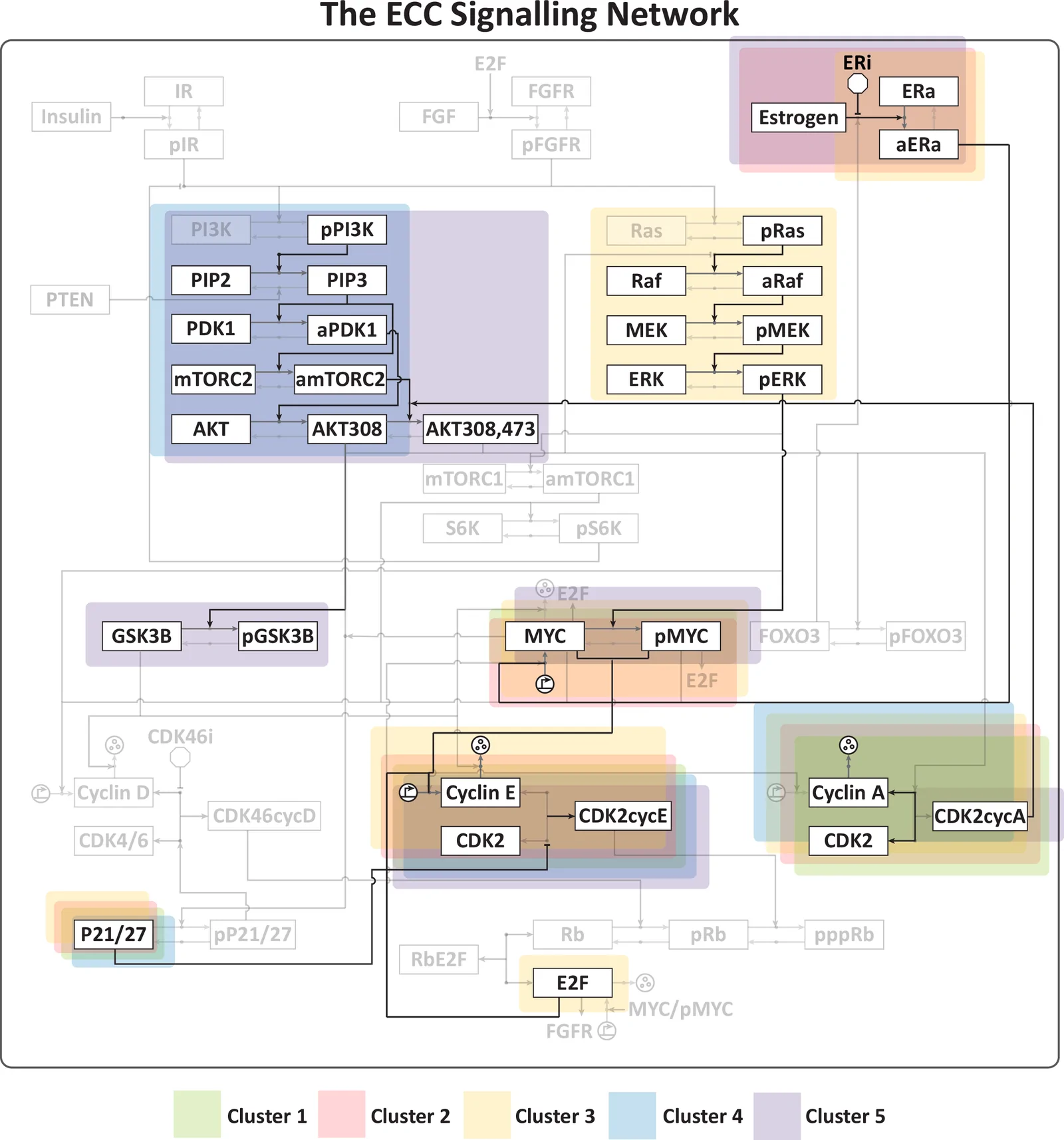
Cluster-specific parameter signatures with the ECC network, shown here for the protein-dynamic CDK2cycE-REB combination. Each figure (Figure 5—figure supplement 5A-I) is the overlay of the respective cluster-specific parameter signatures with the ECC network, for each protein-dynamic combination. Each figure displays the five largest clusters for each protein-dynamic combination.

**Figure 6—figure supplement 8.**
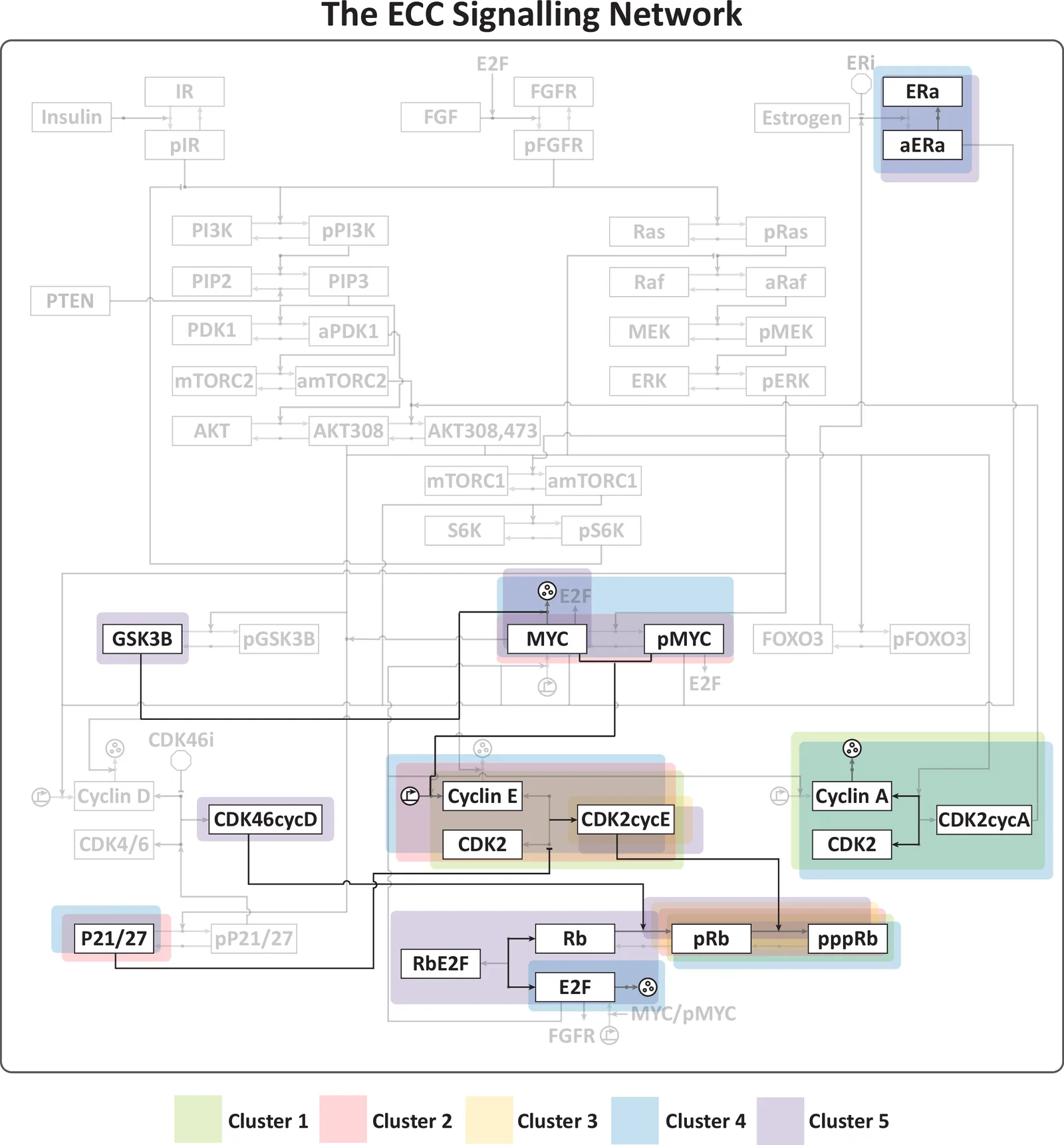
Cluster-specific parameter signatures with the ECC network, shown here for the protein-dynamic pppRb-INC combination. Each figure (Figure 5—figure supplement 5A-I) is the overlay of the respective cluster-specific parameter signatures with the ECC network, for each protein-dynamic combination. Each figure displays the five largest clusters for each protein-dynamic combination.

**Figure 6—figure supplement 9.**
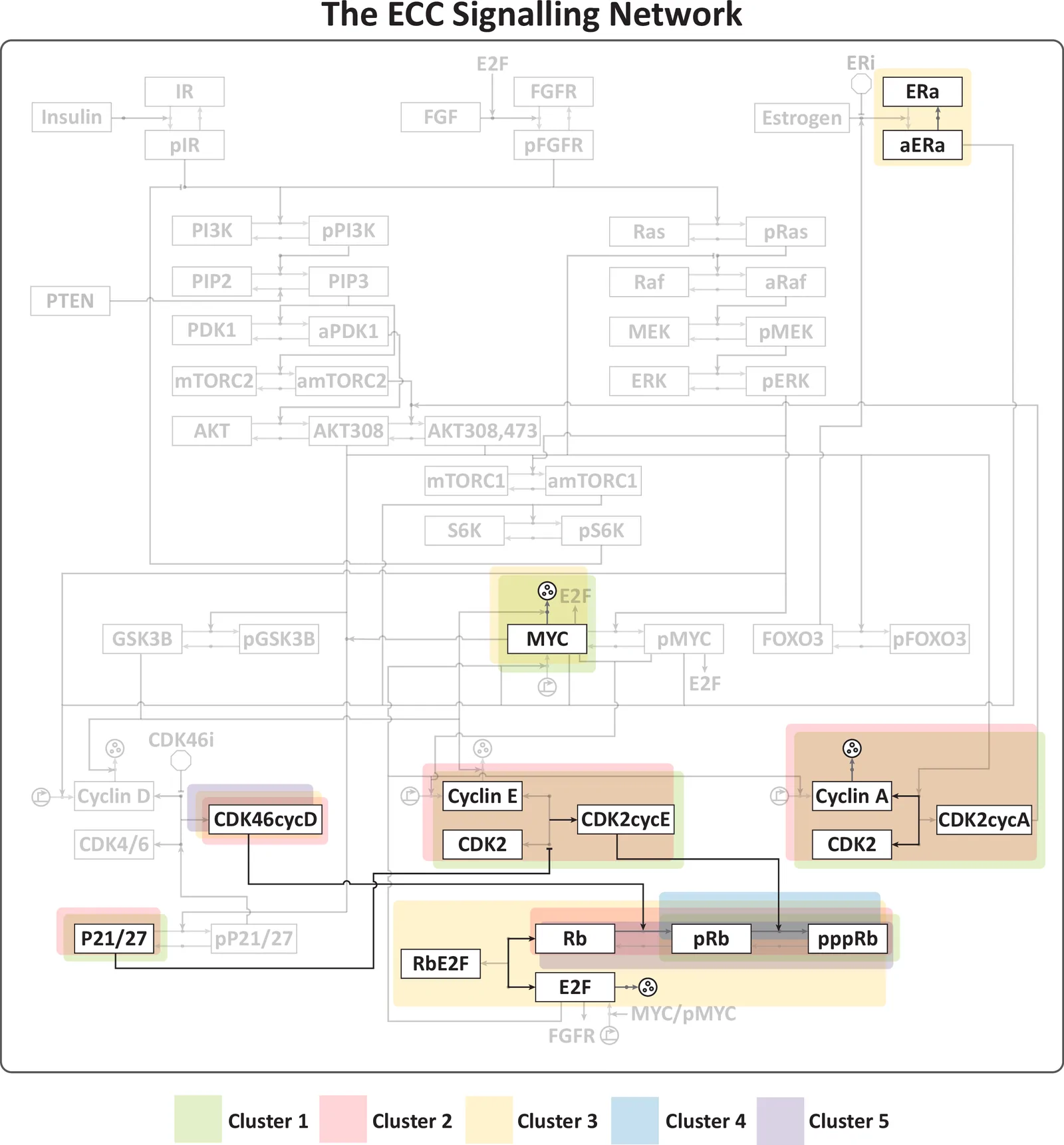
Cluster-specific parameter signatures with the ECC network, shown here for the protein-dynamic pppRb-BIP combination. Each figure (Figure 5—figure supplement 5A-I) is the overlay of the respective cluster-specific parameter signatures with the ECC network, for each protein-dynamic combination. Each figure displays the five largest clusters for each protein-dynamic combination.

**Figure 6—figure supplement 10.**
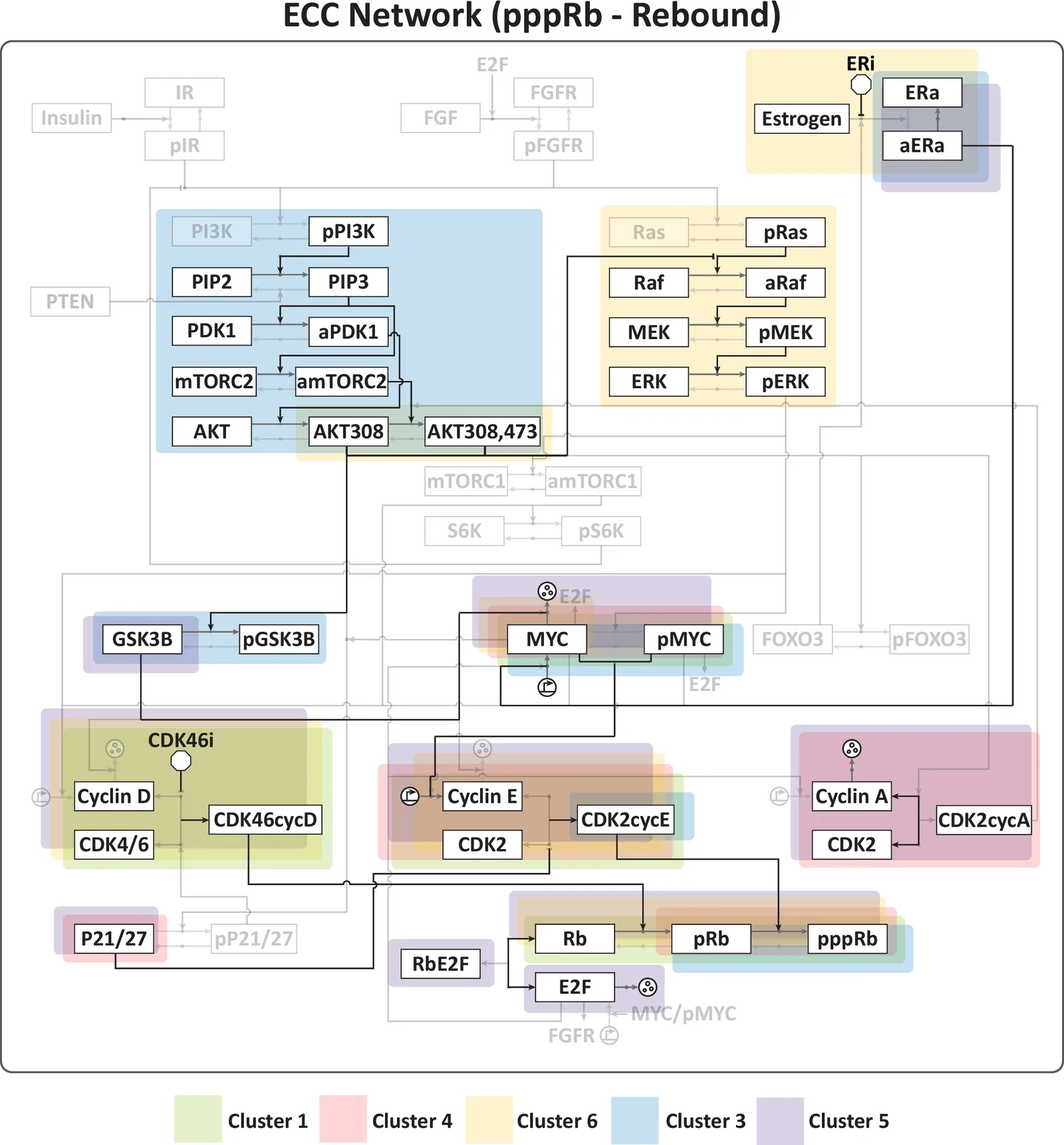
Cluster-specific parameter signatures with the ECC network, shown here for the protein-dynamic pppRb-REB combination. Each figure (Figure 5—figure supplement 5A-I) is the overlay of the respective cluster-specific parameter signatures with the ECC network, for each protein-dynamic combination. Each figure displays the five largest clusters for each protein-dynamic combination.

**Figure 6—figure supplement 11.**
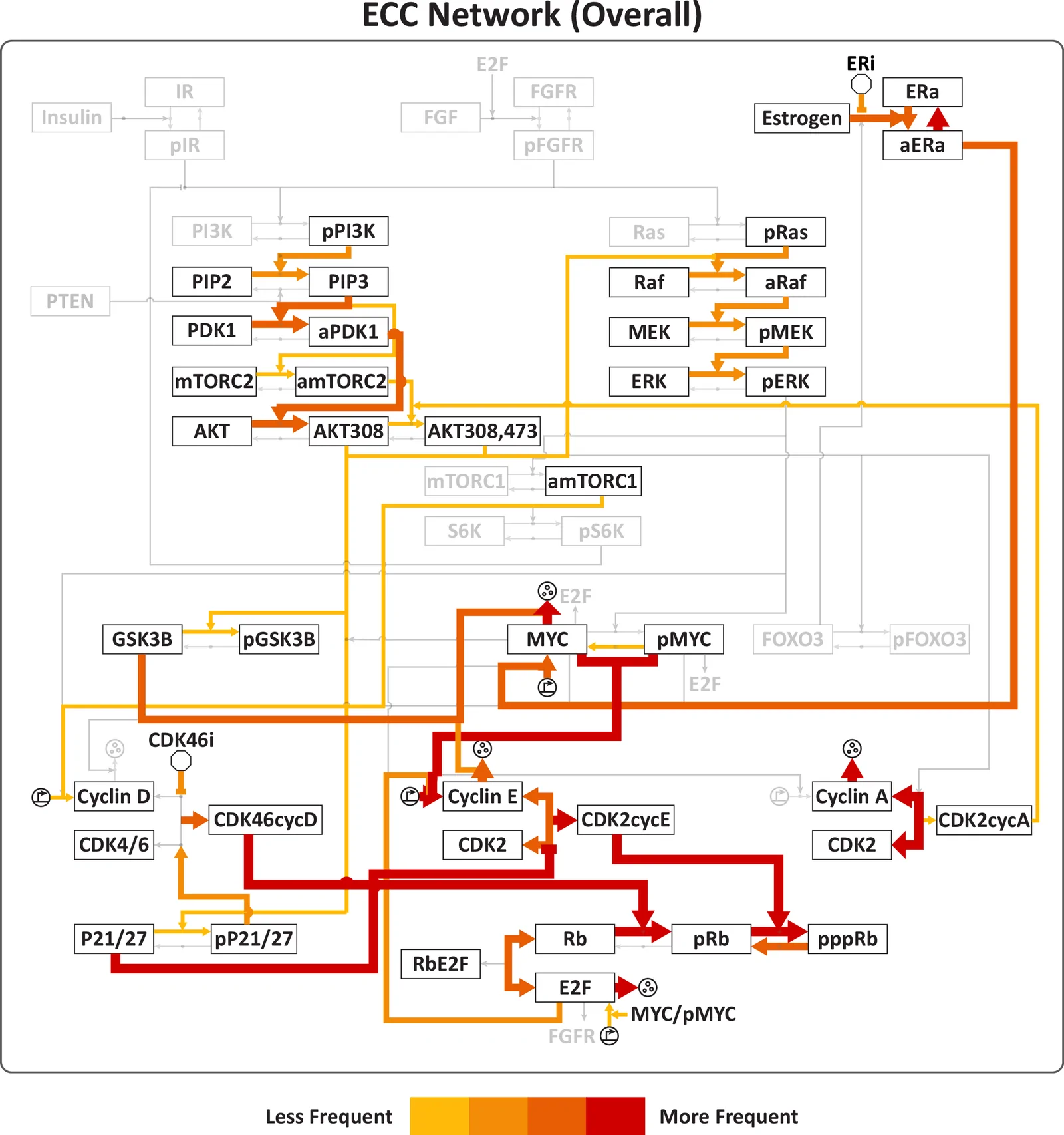
Summary of all the parameter-signature clusters (subnetworks) that drive resistance identified for nine protein-dynamic combinations. This was derived from the individual analysis results in Figure 5—figure supplements 4 and 5A-I.

**Figure 6—figure supplement 12.**
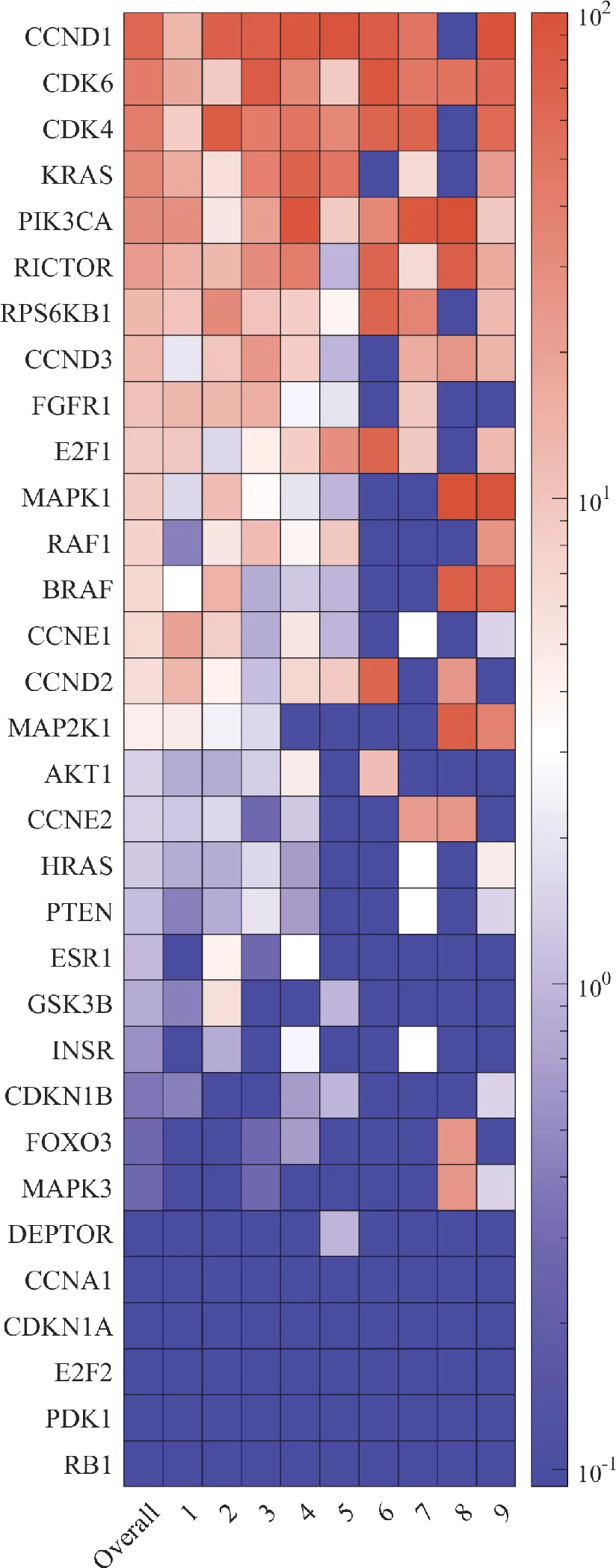
Heatmap showing the clustered differential gene-dependencies of hundreds of cancer cell lines. Genes were filtered for moderate dependency and then subject to hierarchical clustering to identify common signatures of gene dependency across approximately 1000 cancer cell lines. The gene signatures identified in this analysis are quasi-similar to the idea of drug resistance signatures identified using our MDN analysis.

On the other hand, clusters that have less overlap highlight potential differences in underlying resistance mechanisms between patients experiencing seemingly similar drug responses. For the pppRb-rebound group, these clusters included those driven by the PI3K and MAPK pathways, protein degradation regulation (GSK3B), and p21/27 inhibition of the CDK2-cyclin E complex (Figure 6A). Consistently, when we targeted (inhibited) parameters related to these signalling nodes across different clusters, we did not observe universal suppression of pppRb; instead, we saw cluster-specific suppression of pppRb (Figure 6B).

Expanding this analysis to include all nine protein-dynamic combinations, we observed that resistance-promoting subnetworks were widespread throughout the ECC network (Figure 6—figure supplements 1–10). Further, we identified just under 100 robust clusters/subnetworks capable of facilitating resistance dynamics (Supplementary file 6). Finally, to highlight the overlap between protein-dynamic clusters, we combined all of the individual clusters of all of the protein-dynamic combinations (Figure 6—figure supplement 11). This revealed resistance most frequently converges on the activation of CDK2. Mechanisms driving this activation include: the promotion of cyclin E synthesis, reduction in cyclin E degradation, promotion of the formation of the CDK2-cyclin E complex, and suppression of p21- and p27-mediated CDK2 inhibition. There were also moderate contributions to resistance spread throughout both upstream mitogenic pathways. The contribution of the ER to resistance seemed to be context dependent. A strong deactivation rate of the ER contributed to resistance quite frequently, yet paradoxically strong activation of the ER also somewhat frequently contributed.

The above results suggest three things. First, while there is a moderately large number of network states that can drive resistance to dual CDK4/6-ER inhibition, there is very likely only a *finite* number of network states that cancer can exploit to overcome drug perturbation. Second, the moderately large number of network states that facilitate resistance and the observation that any given resistance dynamic can be driven by many different mechanisms highlight the low likelihood of finding broadly applicable resistance mechanisms. Third, some protein nodes and interactions contribute far more frequently to resistance than others. It is quite possible that these frequencies are related to the likelihood and tumourigenicity of mutations affecting these nodes/interactions.

### Qualitative support for MDN modelling-based simulations and predictions

#### Monoculture cell populations demonstrate a significant degree of heterogeneous signalling dynamics in response to CDK4/6 inhibitors

This study provides a theoretical framework that connects heterogeneity, protein signalling dynamics and adaptive resistance mechanisms. To validate this framework, we analysed the literature to pinpoint biological evidence for a number of our key predictions. First, we wanted to observe if and how much drug-response signalling heterogeneity exists in cellular monocultures. Second, we wanted to find evidence that cells possess differential resistance-mediating subnetworks. And third, we wanted to determine if the resistance-mediating subnetworks that we identified agree with known resistance mechanisms.

**Figure 7.**
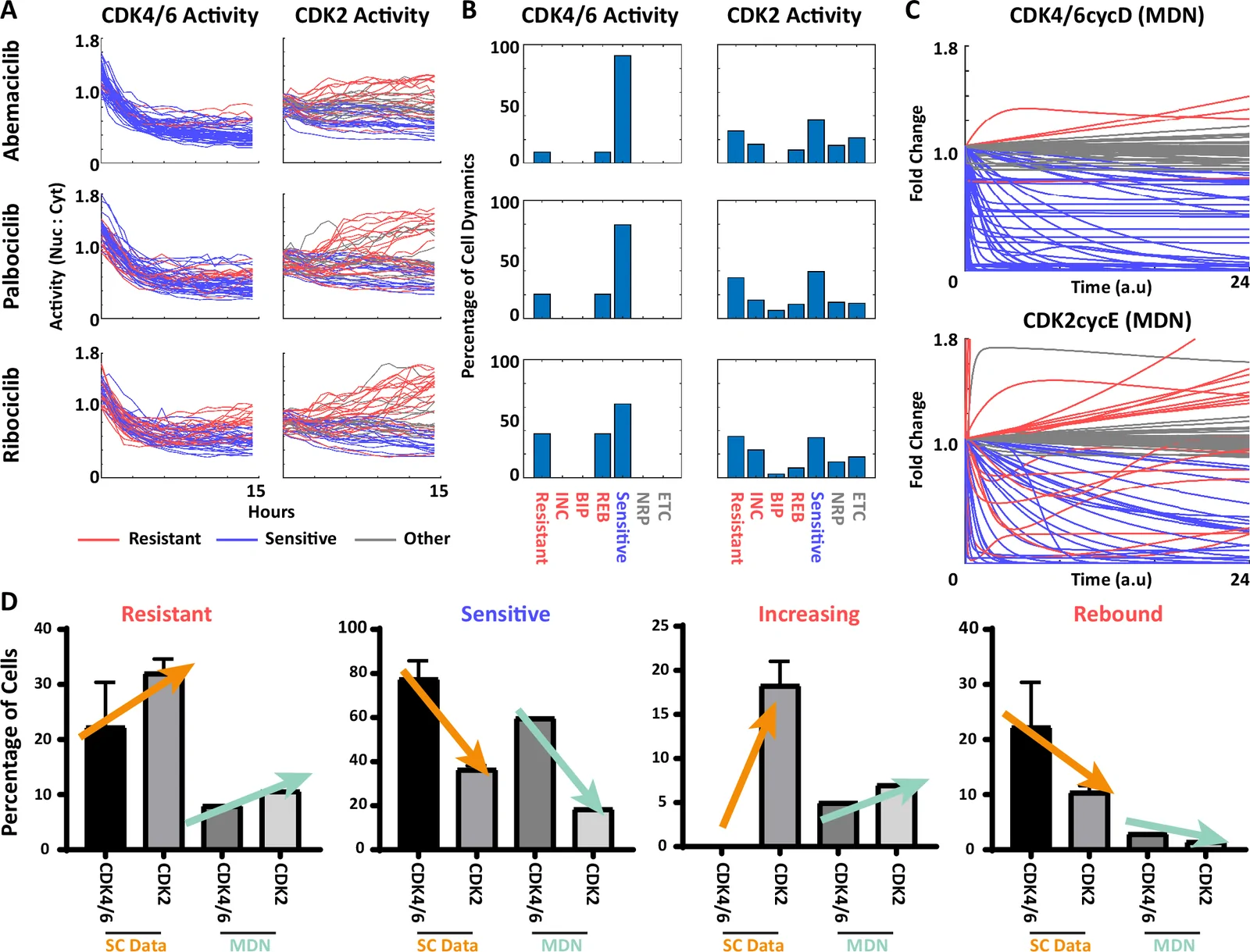
Validation of MDN-based predictions. Qualitative comparisons between single-cell data and our MDN modelling analyses. (**A**) Single-cell time-course responses to CDK4/6 inhibition from Yang et al., 2020. The reporter readout reflects phosphorylation-dependent nucleo-cytoplasmic shuttling; the y-axis is the nuclear/cytoplasmic fluorescence ratio (not fold-change to t=0). Baseline values at t=0 therefore vary across cells (typically ~1–1.8) due to inherent differences in reporter localisation prior to treatment. Red traces: resistance-associated dynamics; blue: sensitivity-associated; grey: no/limited response. (**B**) Frequency of each dynamic category in panel A. (**C**) Representative MDN modelling simulations for CDK4/6·CycD and CDK2·CycE drawn from the model ensemble (parametric variation). Colours denote the same dynamic classes as in A. The distribution of resistant and sensitive dynamics from our simulations are highly correlated with the single-cell data. (**D**) Agreement between single-cell data and MDN modelling simulations quantified by the distribution of dynamic classes across both datasets.

Recently, Yang et al. constructed a novel reporter system that enabled them to investigate the activity of CDK4/6 and CDK2 in individual MCF10A cells in response to three clinically approved CDK4/6 inhibitors: abemaciclib, palbociclib and ribociclib (Yang et al., 2020). Plotting the single-cell time-course data for each drug and protein revealed that, at the single-cell level, there is a wide variety of protein signalling dynamics (Figure 7A). We then subjected this time-course data to our category analysis to calculate the distribution of each protein’s drug response dynamics (Figure 7B). We found that at the level of CDK4/6, the direct target of the three drugs, there was a moderate degree of signalling heterogeneity. Additionally, even the most potent drug (abemaciclib) failed to induce sustained suppression in approximately 10% of the cell population. The activity levels of CDK2, however, showed a much greater degree of signalling heterogeneity, and all three of the drugs only managed to strongly suppress CDK2 activity in a sustained manner in 40% of the population (Figure 7B). We further observed that there were many individual cells that demonstrated *increasing* CDK2 activity in response to CDK4/6 inhibition, a particularly unintuitive result, predicted by our MDN modelling. We then extracted a random sample of model instances from our MDN modelling(parametric variation) and plotted the drug response dynamics of CDK46cycD and CDK2cycE, our model’s equivalent active forms of CDK4/6 and CDK2 (Figure 7C). The resemblance between the distribution of dynamics produced by our model simulations and the single-cell data is striking, both showing very similar proportions of resistant and sensitive dynamics.

Finally, we examined how the distribution of specific dynamics changed when comparing CDK4/6 activity with CDK2 activity. Figure 7D shows that the single-cell data and our MDN modelling analysis exhibit the same general trends: there are more resistant dynamics at the level of CDK2 than at the level of CDK4/6; there are fewer sensitive dynamics at the level of CDK2 than CDK4/6; and there are more increasing dynamics at the level of CDK2. Perhaps most unintuitively, there are fewer rebound dynamics at the level of CDK2 in the single-cell data, which was also predicted by our MDN modelling analysis. Although this analysis is *qualitative*, we demonstrate the capacity for variation in protein activity within a conserved network architecture to drive a wide variety of signalling dynamics, including those associated with resistance, that mirrors the variation in signalling dynamics seen in isogenic single cells.

#### The identified resistance-mediating core subnetworks align with known resistance mechanisms

Previous studies have identified a wealth of mechanisms responsible for driving resistance to CDK4/6 and ER inhibitors. Focusing on mechanisms that have overlap with our model, the vast majority of identified mechanisms align quite well with the predictions made by our MDN modelling analysis, validating the ability of this technique to identify a full spectrum of resistance-driving mechanisms. Moreover, our analysis also makes predictions about additional protein nodes/interactions that have not yet been identified by the literature.

Known mechanisms of resistance to CDK4/6 inhibitors include: Rb loss/mutations (Malorni et al., 2016; Palafox et al., 2022), CDK4/6 overexpression (Yang et al., 2017; Li et al., 2018; Olanich et al., 2015), loss of ER expression (Yang et al., 2017; Takeshita et al., 2018; Iida et al., 2020), loss of PTEN (Costa et al., 2020), reduced expression/activity of p21 and p27 (Kumarasamy et al., 2021; Patel et al., 2018; Iida et al., 2019), activation of mTOR complexes (Michaloglou et al., 2018; Goel et al., 2016), activation of PI3K and PI3K signalling pathways (Takeshita et al., 2018; O’Brien et al., 2020), activation of PDK1 (Jansen et al., 2017), upregulation of FGFR (Turner et al., 2010; Formisano et al., 2019), AKT amplification and/or over-activation (Alves et al., 2021), E2F amplification and/or over-activation (Dean et al., 2010), MAPK pathway activation (Hayes et al., 2019; De Leeuw et al., 2018; Haines et al., 2018), c-Myc activity (Mo et al., 2022; Robinson et al., 2019), cyclin E overexpression and increased CDK2-cyclin E complex formation (Min et al., 2018; Taylor-Harding et al., 2015; Pandey et al., 2020; Hall et al., 2019). Known mechanisms of resistance to ER inhibitors include mutations that induce the constitutive activation of ER (Jeselsohn et al., 2014; Masri et al., 2008), loss of ER activity (Vesuna et al., 2012; Johnston et al., 1995), activation of PI3K and MAPK signalling (Miller et al., 2010; Campbell et al., 2001), overexpression of c-Myc (McNeil et al., 2006; Planas-Silva et al., 2007), and loss of p21 and p27 (Abukhdeir et al., 2008; Massarweh et al., 2006).

The preceding list represents a wealth of known resistance mechanisms that are widely distributed throughout the ECC network. Every single one of the above mechanisms is represented in at least one of our identified resistance-driving subnetworks. To clearly demonstrate how well our MDN modelling analysis captured the known resistance mechanisms, we created a table containing the known resistance mechanisms and the respective resistance-driving parameters identified by our MDN modelling analysis (Supplementary file 7). The ability of MDN modelling to largely capture known resistance mechanisms highlights its potential usefulness for predicting resistance mechanisms to novel drugs and drug targets. Apart from capturing the known resistance mechanisms, our analysis really extends the observation that resistance can emerge from many different nodes within the ECC network and further adds support to the idea that it is critical to treat each patient individually.

## Discussion

Heterogeneity is a defining feature of cancer and a major barrier to durable responses to targeted therapies. Clinical and experimental studies of CDK4/6 inhibitors in ER+ breast cancer consistently show wide inter- and intra-tumour variability in response and multiple, often co-existing resistance mechanisms (Zhou et al., 2023). Yet, despite this wealth of descriptive data, we still lack mechanistic frameworks that can systematically relate underlying molecular heterogeneity to the spectrum of signalling behaviours that enable adaptive drug resistance. In this work, we introduce MDN modelling as such a framework. By sampling broad ensembles of kinetic parameters and protein abundances on a fixed network topology, MDN modelling maps the full distribution of qualitative signalling dynamics (‘meta-dynamics’) accessible to a network architecture and identifies which of these trajectories are compatible with sustained target inhibition versus adaptive escape.

Our first key finding is that even within a single, fixed ECC network topology, variation in reaction kinetics and total protein abundances can generate a surprisingly rich repertoire of qualitative signalling behaviours. We use the term ‘meta-dynamics’ to denote the convergent distribution of these behaviours across a large ensemble of model instances, which represents a theoretical upper limit on what the network can manifest dynamically. Importantly, this repertoire includes trajectories in which continuous CDK4/6 inhibition fails to maintain target suppression and downstream cell-cycle arrest - canonical signatures of adaptive resistance. This provides a concrete mechanistic link between molecular heterogeneity and the well-described phenomenon of rapid signalling rewiring under targeted therapy, in which feedback and crosstalk restore pathway activity despite continued drug exposure (Cremers and Nguyen, 2019; Chandarlapaty, 2012).

We find that adaptive resistance can emerge from heterogeneity in both kinetic parameters and protein abundances, but not symmetrically. However, across the ECC network, we observe that perturbations to kinetic parameters (interaction strengths) are more potent and consistent drivers of adaptive resistance dynamics than changes in total protein abundance. This supports a hierarchical view of control: network topology and interaction strengths largely determine which qualitative behaviours are possible, while protein levels modulate how frequently particular regimes are accessed. That hierarchy is consistent with both systems-level perspectives on cellular heterogeneity (Movasat et al., 2025) and parameter-sensitivity analyses in whole-cell and signalling models, which have shown that many distinct parameter sets can collapse onto a limited set of functional outputs (Babtie and Stumpf, 2017).

Perhaps the most striking observation from our analysis is how rapidly the meta-dynamic distribution converges. Despite sweeping over 90 kinetic parameters and 50 total abundances across nine orders of magnitude, the distribution of qualitative response classes stabilises well before 100,000 accepted model instances. Doubling the ensemble size yields only negligible changes in the frequencies of each dynamic regime, see Figure 3—figure supplement 3. This reflects a strong filtering effect imposed by network topology rather than simply a statistical inevitability of sampling from fixed priors. When parameters or abundances are pushed to extremes, nodes tend to be driven into saturating states, either effectively ‘always off’ or ‘always on’, and become unresponsive to upstream perturbations. These saturated cases fall into the ‘no response’ category and do not generate new, more complex temporal patterns. Consequently, the ECC topology funnels the near-infinite combinatorial parameter space into a finite, recurring set of qualitative behaviours (e.g. monotonic decrease, rebound, biphasic). Such topology-constrained behavioural repertoires have also been noted in recent work that treats heterogeneity by assigning probability distributions to model parameters and analysing the resulting ensemble of single-cell behaviours (Yamada et al., 2018; Spencer et al., 2009). MDN modelling complements these approaches by emphasising qualitative dynamic classes and their frequencies rather than precise parameter posteriors.

At the level of individual nodes and subnetworks, our perturbation analyses reveal that adaptive resistance is a genuinely network-level property. Nearly every node in the ECC model can be implicated in at least one resistance subnetwork when viewed across the full heterogeneity ensemble, echoing the clinical and experimental literature where numerous, sometimes mutually exclusive mechanisms of CDK4/6 inhibitor resistance have been described - ranging from loss of RB1 to cyclin E/CDK2 activation, PI3K–AKT upregulation, FGFR signalling, and alterations in cell-cycle checkpoint control (Zhou et al., 2023). However, MDN modelling does not predict an unstructured ‘anything goes’ landscape. When we rank interactions by how frequently their perturbation participates in resistance-associated trajectories, a small set of hubs and co-regulated modules emerges. In other words, many routes to resistance exist, but they tend to be coordinated through a limited number of recurrent network motifs and subnetworks. This modular picture is consistent with reviews of adaptive resistance in breast cancer, which emphasise convergent rewiring of signalling hubs and feedback nodes despite diverse upstream lesions (Cremers and Nguyen, 2019).

As an additional, orthogonal validation of the resistance subnetworks identified by MDN modellilng, we compared our perturbation-derived resistance modules with gene-dependency patterns from the Cancer Dependency Map (DepMap; Ghandi et al., 2019). Although DepMap is not specific to CDK4/6 inhibition, hierarchical clustering of knockout dependencies for ECC-network genes revealed distinct vulnerability modules across >1000 cell lines (Figure 6—figure supplement 12). The existence of such modules supports the MDN modelling prediction that different cellular contexts rely on distinct but recurrent subnetworks to sustain proliferation, reinforcing the view that resistance is governed by coordinated network-level processes rather than isolated nodes.

Our findings also shed light on why CDK4/6 inhibition resistance is so pervasive yet difficult to capture with single biomarkers. Recent work integrating bulk, single-cell, and trial data has shown that tumours resistant to CDK4/6 inhibition are characterised by increased intra-tumoural heterogeneity and multiple, co-existing resistance signatures, including MYC-driven programmes and altered estrogen-response pathways (Migliaccio et al., 2025). Single-cell imaging and modelling studies have further demonstrated that cell-cycle dynamics and signalling trajectories at the single-cell level are critical determinants of CDK4/6 inhibitor sensitivity (Asghar et al., 2017). Our MDN modelling analysis provides a mechanistic underpinning for these observations: across a broad space of plausible network states, a surprisingly large fraction of configurations yield adaptive resistance-like signalling for key G1-S regulators, and simultaneous durable suppression of all relevant downstream effectors is rare. In such a landscape, static measurements of one or a few nodes are unlikely to robustly stratify patients, because resistance is inherently encoded in distributed, dynamic network behaviour rather than in single static markers.

Despite the theoretical nature of our study, qualitative comparison with single-cell signalling data supports the core results of MDN modelling. Experiments in isogenic breast epithelial cells have shown that CDK4/6 activity reporters display highly heterogeneous dynamics under inhibitor treatment, with some cells remaining durably suppressed, others partially rebounding, and others showing minimal inhibition (Yang et al., 2020; Asghar et al., 2017). Our meta-dynamic distributions reproduce this spectrum of behaviours despite being generated from a much broader parameter ensemble than any one cell line is likely to occupy. We emphasise that this comparison is intentionally qualitative: our goal is not to fit a specific cell line with a calibrated model, but to demonstrate that the ECC topology, when subjected to realistic heterogeneity, naturally gives rise to the types of signalling trajectories observed empirically. The close qualitative agreement between simulated and experimental distributions suggests that even nominally isogenic populations may explore a substantial portion of the network’s meta-dynamic landscape.

As with any model-based framework, MDN modelling has limitations that shape how its predictions should be interpreted. First, we use mass-action and first-order kinetics rather than more detailed Michaelis–Menten or cooperative rate laws. This was essential to keep the parameter space tractable for large-scale exploration, but it means that behaviours relying critically on enzyme saturation or higher-order cooperativity may be under-represented. Second, our parameter and abundance priors are deliberately broad and largely uniform in log-space, whereas biochemical parameters and expression levels in real cells are often better approximated by log-normal or other structured distributions (Bar-Even et al., 2015; Taniguchi et al., 2010; Bengtsson et al., 2005). In this sense, the present work should be viewed as defining what is possible for a given topology rather than estimating what is probable in a specific tumour context. Third, our model is deterministic and does not explicitly capture intrinsic biochemical noise. We argue that sampling across a broad ensemble of parameter and abundance sets can approximate the time-averaged consequences of genetic, epigenetic, and stochastic variation, but intrinsic noise at low copy numbers and rare event dynamics will require explicit stochastic formulations (Kwon et al., 2019). Finally, the ECC model abstracts some multi-protein modules into single nodes to maintain computational tractability. As a result, highly ranked ‘hub’ nodes in our analysis should be interpreted as implicating critical processes or modules, rather than single gene products, as the key levers of resistance.

These limitations point naturally to several avenues for future work. A priority is to constrain MDN modelling ensembles using experimentally inferred parameter and abundance distributions, for example by integrating Bayesian parameter-inference methods that represent cell-to-cell variability as probability distributions over model parameters. Another key direction is to couple MDN-derived resistance subnetworks with data-driven approaches that operate directly on patient-derived single-cell and spatial multi-omics data, which are increasingly used to dissect sample-level heterogeneity and its association with treatment response (Boyeau et al., 2025). Such integrated frameworks could bridge the gap between mechanistic prediction of resistance-enabling subnetworks and empirical identification of patient-specific vulnerabilities. On the dynamical side, embedding stochastic simulations or Langevin approximations within the MDNA framework would allow us to evaluate how intrinsic noise reshapes the meta-dynamic landscape and whether it introduces qualitatively new resistance regimes or primarily modulates the frequencies of existing ones. Finally, our perturbation analyses suggest that a finite set of recurrent resistance modules underpins a vast number of resistant states. This aligns with translational efforts that design rational combination therapies to pre-empt or overcome adaptive resistance by co-targeting signalling hubs and compensatory feedbacks (Yip et al., 2024) and MDN modelling could provide a principled way to enumerate and prioritise such combinations for experimental testing.

In summary, MDN modelling offers a systems-level framework that connects molecular heterogeneity to dynamic signalling behaviour and, ultimately, to adaptive drug resistance. By revealing how a fixed network topology can generate a finite but diverse set of resistance-associated trajectories and by identifying the hubs and modules that recurrently mediate these trajectories, our work complements empirical single-cell and clinical studies and suggests concrete strategies for network-level intervention. Although we have focused on the G1-S transition and CDK4/6 inhibition as a clinically important case study, the MDN modelling approach is broadly applicable to other signalling networks and therapeutic targets. As mechanistic models, single-cell datasets, and computational methods continue to mature, we envisage MDN-like frameworks playing an increasingly important role in predicting how tumours will escape targeted treatments and in guiding combination strategies to keep them one step ahead.

## Materials and methods

**Key resources table:** 

Reagent type (species) or resource	Designation	Source or reference	Identifiers	Additional information
Software, algorithm	MATLAB	https://au.mathworks.com/	N/A	
Software, algorithm	IQM	https://iqmtools.intiquan.com/	N/A	

### ODE model construction, modelling, and simulations

We converted the ECC network (Figure 1) into a system of ODEs using standard biochemical rate laws: catalysed reactions (e.g. phosphorylations) as pseudo-first-order, complex formations as mass action, and transcription, translation and degradation as first-order kinetics. We used these simpler forms to reduce the number of poorly constrained parameters and enable a broader sampling of parameter values in the meta-dynamic analysis. The model comprises 50 state variables, 94 parameters, two mitogenic inputs and two drug inputs. A detailed description of model scope and justification is given in the *Appendix*; and the biochemical reaction lists, ODEs and SBML model files are provided as Supplementary files 1–4.

Simulations were implemented in MATLAB (The MathWorks, Inc 2019a) with IQM toolbox (https://iqmtools.intiquan.com/) to auto-generate ODEs and compile a MEX file for accelerated simulation speed. ODEs were solved using the SUNDIALS CVODES solver, which employs a variable-order, variable-step Backward Differentiation Formula (BDF) method, designed to solve stiff ODE systems.

Each simulation of our model consisted of three phases: (i) *starvation* phase to ensure a steady state equilibrium was reached before mitogenic stimulation (5000 time units); (ii) *fed* with constant mitogenic stimulation (100 nM of insulin and FGF) until quasi-steady state (5000 time units); and (iii) treatment with CDK4/6i and ER inhibitor (3000 time units). Mitogens and drugs were modelled as step inputs at the start of their respective phases and then held constant for the remaining simulation time, isolating network-intrinsic adaptation rather than responses to input removal. Drug inhibitions were modelled by reducing the relevant parameter values by a fixed fraction.

### Sampling and filtering of model instances

MDN modelling involves random sampling of parameter sets to create unique model instances. We drew random parameter sets and species conserved-totals from a log-transformed uniform distribution and continued sampling until we obtained N=100,000 accepted instances for each analysis. The range of sampled parameter values spanned 10^–5^ to 10^4^ and the range of conserved totals spanned 10^0^–10^4^. These ranges reflect the spans measured in biological contexts (Bar-Even et al., 2015). To ensure sampled parameter sets reflected plausible biological contexts while retaining rare network states, we applied several filtering steps. An instance was accepted if it met the following: (i) *Quasi–steady state* reached in the starvation and fed phases, defined by very small changes across species within a time cap (1% change in concentration over the final 100 time steps); (ii) *Non-trivial on-target drug effect* during treatment (e.g. ≥5% reduction in the CDK4/6 activity proxy or [CDK4/6·CycD] level for CDK4/6i and in [active ER] level for ERi). Moreover, we note that multi-timescale signalling models are often stiff (Städter et al., 2021; Resat et al., 2009), and random parameter draws can create extreme early transients that are numerically hard to integrate even with stiff solvers. We therefore excluded these parameter instances, which accounts for only <1% of total sampled sets.

Applying these filters, ~40–45% of instances did not reach steady state within the allotted time, and ~50–55% did not meet the minimum drug-response criterion. Approximately 10% satisfied all criteria and were retained for analysis. Importantly, we employed ‘rejection sampling’ and continued drawing until we had N=100,000 accepted instances that satisfied all the criteria.

### Quantitative definitions of dynamic patterns

A major component of MDN modelling is the analysis of the qualitative shape of individual protein dynamics. This enables us to group together model instances that produce broadly similar behaviours for any proteins being investigated. We can then analyse these groups to identify and elucidate shared network structures that drive the behaviour. To this end, we defined six qualitative protein dynamic categories: increasing, decreasing, biphasic, rebound, no response and other.

To assess the qualitative shape of each protein dynamic, we developed a series of mathematical restraints that were capable of sorting protein simulations into their appropriate categories. First, each protein dynamic was normalised to its initial value. A protein’s dynamic was categorised as *increasing* (*INC*) if (*i*) the final concentration exceeded 20% of its initial concentration, (*ii*) over the entire simulated time period it never dropped below 10% of its initial concentration, and (*iii*) the final concentration was within 10% of the maximum concentration. It was categorised as *decreasing* (*DEC*) if (*i*) the final concentration was at least 20% lower than the initial concentration, (*ii*) never went above 10% of its initial concentration, and (*iii*) the final concentration was within 10% of the minimum concentration. It was categorised as *biphasic* (*BIP*) if (*i*) the maximum concentration exceeded the initial concentration by 20% and (*ii*) the final concentration was at least 10% lower than the maximum concentration. It was categorised as *rebound* (*REB*) if (*i*) the minimum concentration was at least 20% lower than the initial concentration and (*ii*) the final concentration was at least 10% bigger than the minimum concentration. It was categorised as *no-response* (*NRP*) if the concentration never exceeded more or less than 10% of the initial concentration. Finally, it was categorised as *other* (*ETC*) if the protein’s dynamic did not fit one of the previously described categories.

### Key model output proteins

There is strong evidence to suggest that the linear cascade of cell cycle regulator progression, wherein the activation of CDK4/6 leads to the activation of CDK2-cyclin E complex, which in turn leads to the activation of CDK2-cyclin A, is overly simplistic (Stallaert et al., 2022; Tyson and Novák, 2015). If this traditional understanding was correct, the inhibition of CDK4/6 activity would robustly inhibit cell cycle progression, yet this is not the case (Iorio et al., 2016). A number of studies have suggested that the activation or reactivation of proteins downstream of CDK4/6 can enable a cell to overcome the cell cycle inhibitory effects of CDK4/6 targeting drugs and enable cell cycle progression. Proteins that have been shown to demonstrate this phenomenon include phosphorylated Rb and the active CDK2-cyclin E complex (Herrera-Abreu et al., 2016; Min et al., 2018; Taylor-Harding et al., 2015). As such, we have focused on the dynamics of monophosphorylated Rb (*pRb*), hyperphosphorylated Rb (*pppRb*), and the CDK2-cyclin E complex (*CDK2cycE*) throughout this project, as key mediators of adaptive resistance to CDK4/6 and ER inhibitors.

### Protein knockdown perturbation analyses

To assess the effects of parameters on protein dynamics we performed high-throughput perturbation analyses wherein each parameter was knocked down one at a time (OAT) and the effect on the nominated output assessed. We chose the OAT design intentionally to obtain causal, first-order attribution of control points across a broad parameter ensemble without confounding from simultaneous co-inhibition. This provides an interpretable ranking of primary drivers that is consistent with the paper’s mechanistic focus.

## Data Availability

Code is available: https://github.com/IntegratedNetworkModellingLab/Meta_Dynamic_Analysis_eLife, copy archived at DrHartResearch, 2026.

## Additional files

Supplementary file 1. Early cell cycle (ECC) model in biochemical reaction format. Model file describing the ECC network in biochemical reaction format, including model species, initial conditions, parameters, variables, and reaction-rate expressions.

Supplementary file 2. Early cell cycle (ECC) model ordinary differential equations (ODEs). Plain-text representation of the ECC model containing the system of ODEs, initial conditions, parameter values, model variables, and reaction-rate equations.

Supplementary file 3. Early cell cycle (ECC) model in Systems Biology Markup Language (SBML) format. SBML representation of the ECC model, including model species, parameters, assignment rules, and biochemical reactions.

Supplementary file 4. Typeset ordinary differential equations (ODEs) for the early cell cycle (ECC) model. Typeset representation of the complete system of ODEs and reaction-rate equations defining the ECC model, provided to facilitate interpretation and reproducibility.

Supplementary file 5. Nominal parameter units and values. Nominal parameter values and units used for the ECC model. These values provide the base parameter set used in analyses examining variation in protein abundance/initial conditions.

Supplementary file 6. Robust parameter signatures capable of facilitating resistance-associated dynamics. Tables summarising the robust parameter-signature clusters identified by MDN modelling analysis across the resistance-associated protein–dynamic combinations. For each cluster, the tables report its frequency and the parameters contributing to the corresponding resistance signature.

Supplementary file 7. Known resistance mechanisms and corresponding resistance-driving model parameters. Comparison of experimentally reported mechanisms of resistance to CDK4/6 and oestrogen receptor inhibition with the corresponding resistance-driving parameters identified by MDN modelling analysis.

MDAR checklist

## Appendix 1

### Description of model scope and construction

To keep the model to a tractable size we selected only two major mitogenic signalling pathways, PI3K-AKT and MAPK, frequently mutated in cancer. For this model we chose the insulin receptor (IR) and the fibroblast growth factor receptor (FGFR) to be representative of the many receptor tyrosine kinases (RTKs) capable of stimulating these pathways. The PI3K-AKT signalling pathway feeds into the cell cycle machinery through two parallel mechanisms. First is through the inactivation of GSK3B and the second is through the promotion of cyclin D transcription by active mTORC1 (Vasan et al., 2019; Wang et al., 2019). Unphosphorylated GSK3B promotes the degradation of cyclin E, A, and the transcription factor Myc, and its phosphorylation by active AKT deactivates this ability and promotes cell cycle progression (Pich et al., 2022; Knudsen et al., 2022). The transcription of cyclins enables them to bind to and activate their cognate CDKs and similarly promote cell cycle progression. Contained within the PI3K-AKT motif is a negative feedback loop wherein activated ribosomal protein S6 kinase beta (S6K) inhibits insulin receptor mediated signalling (Jubran et al., 2022).

The promotion of cell cycle progression by the MAPK cascade has been included in this model in three ways. The first is the direct promotion of cyclin D transcription by the activation of the AP1 transcription factor by ERK (Dagogo-Jack and Shaw, 2018). The second is through the phosphorylation of Myc, which stabilises it and prevents its degradation (Turke et al., 2010). Both phosphorylated and unphosphorylated forms of Myc promote cell cycle progression in many ways. Myc promotes cyclin transcription, promotes the transcription of E2F and enhances E2F’s transcriptional activity and enhances the ability of AKT to phosphorylate p21 and p27 (Patel et al., 2022; Cassidy, 2019; Herrera-Abreu et al., 2016; Blombery et al., 2019; Yun et al., 2008). The third way ERK promotes cell cycle progression is promotion of estrogen receptor (ER) activity (Xue and Liang, 2012). In particular, ERK enhances the ability of the ER to initiate cyclin D transcription (Smyth et al., 1998). There is a negative feedback loop within the MAPK cascade where the activation of ERK causes it to inhibit upstream Ras signalling (Jin et al., 2020). There is also an important crosstalk mechanism that exists between the PI3K-AKT and MAPK signalling pathways; the inhibition of Raf activation by active AKT (Li et al., 2022).

E2F is thought to be one of the most important transcription factors in pushing the cell cycle towards S-phase as it drives transcription of cyclins E and A (Cremers and Nguyen, 2019; Wright et al., 2021). E2F is inhibited by unphosphorylated Rb, but this inhibition is partially relieved by the monophosphorylation of Rb by the active CDK4/6-cyclin D complex (Ma et al., 2016). Canonically, the partial release of inhibition enables the transcription of cyclin E, which in turn activates CDK2. Active CDK2-cyclin E complexes then hyperphosphorylate Rb, fully relieving the inhibition of E2F and enabling the transcription of cyclin A (Ma et al., 2016). The transcription of cyclin A and its binding to CDK2 represent a point at which the cell has well and truly entered the S-phase of the cell cycle. CDK2-cyclin A complexes have been shown to stabilise the double phosphorylated form of AKT whereas hyperphosphorylated Rb has been shown to be capable of translocating into the cytoplasm and inhibiting the activation of mTORC2 (Herrera-Abreu et al., 2016; Park et al., 2020). In our model, p21 and p27 are included as a single species where the phosphorylated form promotes CDK4/6-cyclin D complex formation and the unphosphorylated form inhibits the complex formation of CDK2-cyclin E (Nguyen and Kholodenko, 2016; Bachmann et al., 2012).

Due to its importance in ER +BC, ER was also included in our model. Its main function is the promotion of cyclin D transcription, but it is also capable of promoting Myc transcription (Shankar et al., 2019; Clarke and Fisher, 2020). FOXO3 was included due to its ability to promote ER activity by upregulating its transcription (Altrock et al., 2015). FOXO3 is regulated by AKT whereby AKT phosphorylates FOXO3, causing it to become localised in the cytosol and unable to regulate transcription (Ghomlaghi et al., 2021). The network schematic generated from these observations can be seen in Figure 2. It should be noted that some protein species from with the mitogenic and cell cycle pathways were excluded/abstracted to keep the overall model to a size that could be effectively explored using the available computational resources.

The following is a brief explanation/justification of each reaction within the model. Any departure from the biological cause-and-effect relationships between proteins was done to reduce model complexity and thus enable greater and more in-depth computational analysis. In general, equations utilise first-order kinetics. This choice was made to reduce the number of parameters for the same reasons we attempted to reduce model complexity, to reduce the model’s computational burden. While utilising more sophisticated rate laws, such as Michaelis-Menten kinetics, may improve the biological accuracy of the model description, we believe that the loss is minimal and worth the gain in our ability to perform more sophisticated analyses. In the future, we would like to explore smaller models but utilise more biologically accurate rate laws to precisely determine the effect of rate law choice on model dynamics.

#### R1: IR ⇔ pIR

This reaction describes the dimerization and autophosphorylation of the insulin receptor, stimulated by insulin.

#### R2: PI3K ⇔ pPI3K

This reaction captures the binding of IRS to IR, recruitment of the PI3K regulatory unit and the recruitment of the PI3K catalytic subunit. As IRS is inhibited by active S6K, this component of the reaction is inhibited by S6K. PI3K is also able to be activated by the FGF receptor, though GRB2.

#### R3: PIP2 ⇔ PIP3

PIP2 is phosphorylated by active PI3K to become PIP3.

#### R4: PDK1<=>aPDK1

PDK1 is recruited to PIP3, bringing it into proximity to its targets. This is modelled as an active form of PDK1. As PIP3 is usually far in excess of PDK1, this reaction is modelled as PIP3 catalysing PDK1.

#### R5: mTORC2<=>amTORC2

mTORC2 is also recruited to PIP3, bringing it in proximity to its targets. This is modelled as an active form of mTORC2. As PIP3 is usually far in excess of mTORC2, this reaction is modelled as PIP3 catalysing mTORC2.

#### R6: AKT <=>pAKT308

Akt is phosphorylated at T308 by PDK1.

#### R7: pAKT308<=>ppAKT308473

Akt is phosphorylated at S473 by mTORC2. Akt phosphorylation has been modelled as a linear progression to reduce complexity. Akt is fully active when it is dual phosphorylated, but it is still active with only a single phosphorylation. This has been incorporated into the model using an AKTscale parameter, which scales the activity of the dual phosphorylated Akt. CDK2-cyclinA has also been demonstrated to phosphorylate the tail of Akt, promoting the dual phosphorylation of Akt, thus CDK2cycA has been modelled as promoting this reaction.

#### R8: mTORC1<=>amTORC1

Akt inhibits TSC2, which in turn inhibits mTORC1. TSC2 has been excluded from this model, and as such Akt activates mTORC1.

#### R9: S6K<=>pS6 K

S6K is phosphorylated and activated by mTORC1.

#### R10: FGFR <=>pFGFR

Similar to IR, this reaction represents the activation of the FGF receptor by the binding of its ligand FGF. E2F is capable of stimulating the expression of FGFR, but as FGFR is not able to be synthesised in this model, this process has been modelled as E2F promoting FGFR activity.

#### R11: Ras <=>aRas

Ras is activated through its recruitment to the membrane, which is achieved by the recruitment of GRB2 to both IR and FGFR. GRB2 has not been included in this model and so the activation of Ras has been modelled as being catalysed by the active form of both receptors. SOS is also involved in the recruitment of Ras and is inhibited by ERK. Thus, as SOS is also not in this model, this reaction has been modelled as being inhibited by ERK.

#### R12: Raf <=>aRaf

Raf is recruited to the membrane by Ras. Raf is also phosphorylated and inhibited by Akt.

#### R13: MEK <=>pMEK

Raf phosphorylates and activates MEK.

#### R14: ERK <=>pERK

MEK phosphorylates and activates ERK.

#### R15: ERa <=>aERa

Like IR and FGFR, the ER is activated by the binding of its cognate ligand, oestrogen. ER synthesis can also be promoted by FOXO3, and this relationship has been modelled in a similar fashion to the promotion of FGFR activation by E2F. This reaction is also inhibited by the ER drug, which has been modelled as inhibiting the activation of ER by oestrogen; the inhibition was modelled by reducing the value of the activation parameter by a fixed fraction.

#### R16:=>Myc

Myc synthesis can be promoted by the ER. This promotion can be promoted by ERK, cyclin D, and E2F.

#### R17: Myc =>

GSK3B promotes Myc degradation.

#### R18: Myc <=>pMyc

ERK phosphorylates and stabilises Myc.

#### R19: GSK3b<=>pGSK3b

Akt phosphorylates and inhibits GSK3B.

#### R20: FOXO3<=>pFOXO3

Foxo3 is phosphorylated by Akt, which excludes it from the nucleus and inhibits its transcriptional activity. Thus, pFOXO3 is an inactive form of FOXO3.

#### R21:=>cycD

Cyclin D synthesis is independently regulated by mTORC1, ERK, Myc and ER. The mechanisms all vary, where mTORC1 promotes general protein synthesis, ERK and Myc have been shown to promote transcription factors that in turn regulate cyclin D synthesis, and ER can directly regulate cyclin D synthesis. ERK and cyclin D can also promote the regulation of cyclin D synthesis by ER.

#### R22: cycD =>

Cyclins are generally unstable proteins with short half-lives, but this process can be dramatically sped up by GSK3B phosphorylation targeting them for ubiquitin mediated degradation.

#### R23:=>cycE

Cyclin E synthesis is directly regulated by E2F and Myc. Myc is also known to promote the activity of E2F.

#### R24: cycE =>

Similar to cyclin D, cyclin E degradation is regulated by GSK3B.

#### R25:=>cycA

Similar to cyclin E, cyclin A has been shown to be regulated by E2F and Myc.

#### R26: cycA =>

Cyclin A degradation is regulated by proteins not included in this model.

#### R27: CDK46+cycD <=> CDK46cycD

The formation of the CDK4/6-cyclin D complex has been shown to be promoted by p21 and p27. The CDK4/6 drug has been modelled to inhibit the formation of this complex; the inhibition was modelled by reducing the value of the association parameter by a fixed fraction.

#### R28: CDK2+cycE <=> CDK2cycE

Canonically, p21 and p27 are CDK2 inhibitors, and so this relationship has been modelled as p21p27 inhibiting the formation of this complex.

#### R29: CDK2+cycA <=> CDK2cycA

There is evidence to suggest that Akt phosphorylation can promote the formation of the CDK2-cyclin A complex.

#### R30: p2127<=>pp2127

Akt can phosphorylate and stabilise p21.

#### R31:=>E2 F

Myc can promote the synthesis of E2F.

#### R32: E2F =>

The regulation of E2F degradation is performed by proteins not included in this model.

#### R33: E2*F*+Rb <=> RbE2F

Unphosphorylated Rb binds to E2F inhibiting its ability to regulate transcription.

#### R34: Rb <=>pRb

The CDK4/6-cyclin D complex monophosphorylated E2F, partially relieving the inhibition of Rb on E2F.

#### R35: pRb <=>pppRb

CDK2-cyclin E hyperphosphorylates Rb, leading to its full dissociation from E2F.

#### Error convergence

Early experimental observations seemed to suggest that the distribution of protein dynamics was not changing dramatically when we increased the number of model instances being tested. This seemed to suggest that despite the massive number of possible model instances that could be generated given the parameter and initial condition hyperspaces we were searching, the distribution of protein dynamics was fixed well before this number was even close to being reached. To confirm this hypothesis, we calculated the difference between increasingly large populations of model instances to identify if/when the distribution of protein dynamics was converging to a fixed distribution. To measure the convergence, we first recorded the frequency of each dynamic for each protein was measured for N and 2N-sized populations of model instances/parameter sets. We then calculated the squared difference, or error, between the frequencies for each protein species, summed all of the error values and then divided by the number of observations (6x50: dynamic categories x number of state variables). In this way we were able to calculate the mean squared error (MSE) as the population size increased and measure the rate of convergence for this particular network topology. See Figure 3—figure supplement 3.

In addition to the MSE analysis described above, we also repeated the model instance generation and analysis in triplicate. By this we mean we generated 3 groups of 100,000 unique model instances for a total of 300,000 unique model instances. Each group was analysed separately, producing 3 meta-dynamic maps. These distributions of dynamics were then compared. We calculated that the average standard deviation between each distribution of each protein and found it to be 0.000329 or 0.0329% i.e. there is only a 0.0329% difference in the quantity of each protein dynamic on average between replicates. From our MSE analysis and the incredibly high similarity of distributions between completely independent replicates we conclude that the distribution of dynamics has well and truly converged by 100,000 parameter sets.
