# Supplementary material for: Systematic analysis of network-driven adaptive resistance to CDK4/6 and oestrogen receptor inhibition using meta-dynamic network modelling
Source: eLife. 2026 Aug 27;12:RP87710. doi: 10.7554/eLife.87710 (PMC13521565; doi:10.7554/eLife.87710)
Supplement: Supplementary file 1 [file elife-87710-supp3.pdf]

```
<?xml version="1.0" encoding="UTF-8"?>
<sbml xmlns="http://www.sbml.org/sbml/level2" level="2" version="1">
 <model name="CDK4/6Inhibition-CellCycleandMitogenicPathways">
  <listOfCompartments>
   <compartment id="rootCompartment" name="rootCompartment"
spatialDimensions="3" size="1" constant="true"/>
  </listOfCompartments>
  <listOfSpecies>
   <species id="IR" name="IR" compartment="rootCompartment" initialAmount="100"
hasOnlySubstanceUnits="true" boundaryCondition="false" constant="false"/>
   <species id="pIR" name="pIR" compartment="rootCompartment" initialAmount="0"
hasOnlySubstanceUnits="true" boundaryCondition="false" constant="false"/>
   <species id="PI3K" name="PI3K" compartment="rootCompartment"
initialAmount="100" hasOnlySubstanceUnits="true" boundaryCondition="false"
constant="false"/>
   <species id="pPI3K" name="pPI3K" compartment="rootCompartment"
initialAmount="0" hasOnlySubstanceUnits="true" boundaryCondition="false"
constant="false"/>
   <species id="PIP2" name="PIP2" compartment="rootCompartment"
initialAmount="100" hasOnlySubstanceUnits="true" boundaryCondition="false"
constant="false"/>
   <species id="PIP3" name="PIP3" compartment="rootCompartment"
initialAmount="0" hasOnlySubstanceUnits="true" boundaryCondition="false"
constant="false"/>
   <species id="PDK1" name="PDK1" compartment="rootCompartment"
initialAmount="100" hasOnlySubstanceUnits="true" boundaryCondition="false"
constant="false"/>
   <species id="aPDK1" name="aPDK1" compartment="rootCompartment"
initialAmount="0" hasOnlySubstanceUnits="true" boundaryCondition="false"
constant="false"/>
   <species id="mTORC2" name="mTORC2" compartment="rootCompartment"
initialAmount="100" hasOnlySubstanceUnits="true" boundaryCondition="false"
constant="false"/>
```

<species id="amTORC2" name="amTORC2" compartment="rootCompartment" initialAmount="0" hasOnlySubstanceUnits="true" boundaryCondition="false" constant="false"/>

<species id="AKT" name="AKT" compartment="rootCompartment" initialAmount="100" hasOnlySubstanceUnits="true" boundaryCondition="false" constant="false"/>

<species id="pAKT308" name="pAKT308" compartment="rootCompartment" initialAmount="0" hasOnlySubstanceUnits="true" boundaryCondition="false" constant="false"/>

<species id="ppAKT308473" name="ppAKT308473" compartment="rootCompartment" initialAmount="0" hasOnlySubstanceUnits="true" boundaryCondition="false" constant="false"/>

<species id="mTORC1" name="mTORC1" compartment="rootCompartment" initialAmount="100" hasOnlySubstanceUnits="true" boundaryCondition="false" constant="false"/>

<species id="amTORC1" name="amTORC1" compartment="rootCompartment" initialAmount="0" hasOnlySubstanceUnits="true" boundaryCondition="false" constant="false"/>

<species id="S6K" name="S6K" compartment="rootCompartment" initialAmount="100" hasOnlySubstanceUnits="true" boundaryCondition="false" constant="false"/>

<species id="pS6K" name="pS6K" compartment="rootCompartment" initialAmount="0" hasOnlySubstanceUnits="true" boundaryCondition="false" constant="false"/>

<species id="FGFR" name="FGFR" compartment="rootCompartment" initialAmount="100" hasOnlySubstanceUnits="true" boundaryCondition="false" constant="false"/>

<species id="pFGFR" name="pFGFR" compartment="rootCompartment" initialAmount="0" hasOnlySubstanceUnits="true" boundaryCondition="false" constant="false"/>

<species id="Ras" name="Ras" compartment="rootCompartment" initialAmount="100" hasOnlySubstanceUnits="true" boundaryCondition="false" constant="false"/>

<species id="aRas" name="aRas" compartment="rootCompartment" initialAmount="0" hasOnlySubstanceUnits="true" boundaryCondition="false" constant="false"/>

<species id="Raf" name="Raf" compartment="rootCompartment" initialAmount="100" hasOnlySubstanceUnits="true" boundaryCondition="false" constant="false"/>

<species id="aRaf" name="aRaf" compartment="rootCompartment" initialAmount="0" hasOnlySubstanceUnits="true" boundaryCondition="false" constant="false"/>

<species id="MEK" name="MEK" compartment="rootCompartment" initialAmount="100" hasOnlySubstanceUnits="true" boundaryCondition="false" constant="false"/>

<species id="pMEK" name="pMEK" compartment="rootCompartment" initialAmount="0" hasOnlySubstanceUnits="true" boundaryCondition="false" constant="false"/>

<species id="ERK" name="ERK" compartment="rootCompartment" initialAmount="100" hasOnlySubstanceUnits="true" boundaryCondition="false" constant="false"/>

<species id="pERK" name="pERK" compartment="rootCompartment" initialAmount="0" hasOnlySubstanceUnits="true" boundaryCondition="false" constant="false"/>

<species id="ERa" name="ERa" compartment="rootCompartment" initialAmount="100" hasOnlySubstanceUnits="true" boundaryCondition="false" constant="false"/>

<species id="aERa" name="aERa" compartment="rootCompartment" initialAmount="0" hasOnlySubstanceUnits="true" boundaryCondition="false" constant="false"/>

<species id="Myc" name="Myc" compartment="rootCompartment" initialAmount="0" hasOnlySubstanceUnits="true" boundaryCondition="false" constant="false"/>

<species id="pMyc" name="pMyc" compartment="rootCompartment" initialAmount="0" hasOnlySubstanceUnits="true" boundaryCondition="false" constant="false"/>

<species id="GSK3b" name="GSK3b" compartment="rootCompartment" initialAmount="100" hasOnlySubstanceUnits="true" boundaryCondition="false" constant="false"/>

<species id="pGSK3b" name="pGSK3b" compartment="rootCompartment" initialAmount="0" hasOnlySubstanceUnits="true" boundaryCondition="false" constant="false"/>

<species id="FOXO3" name="FOXO3" compartment="rootCompartment" initialAmount="100" hasOnlySubstanceUnits="true" boundaryCondition="false" constant="false"/>

<species id="pFOXO3" name="pFOXO3" compartment="rootCompartment" initialAmount="0" hasOnlySubstanceUnits="true" boundaryCondition="false" constant="false"/>

<species id="cycD" name="cycD" compartment="rootCompartment" initialAmount="0" hasOnlySubstanceUnits="true" boundaryCondition="false" constant="false"/>

<species id="cycE" name="cycE" compartment="rootCompartment" initialAmount="0" hasOnlySubstanceUnits="true" boundaryCondition="false" constant="false"/>

<species id="cycA" name="cycA" compartment="rootCompartment" initialAmount="0" hasOnlySubstanceUnits="true" boundaryCondition="false" constant="false"/>

<species id="CDK46" name="CDK46" compartment="rootCompartment" initialAmount="100" hasOnlySubstanceUnits="true" boundaryCondition="false" constant="false"/>

<species id="CDK2" name="CDK2" compartment="rootCompartment" initialAmount="100" hasOnlySubstanceUnits="true" boundaryCondition="false" constant="false"/>

<species id="CDK46cycD" name="CDK46cycD" compartment="rootCompartment" initialAmount="0" hasOnlySubstanceUnits="true" boundaryCondition="false" constant="false"/>

<species id="CDK2cycE" name="CDK2cycE" compartment="rootCompartment" initialAmount="0" hasOnlySubstanceUnits="true" boundaryCondition="false" constant="false"/>

<species id="CDK2cycA" name="CDK2cycA" compartment="rootCompartment" initialAmount="0" hasOnlySubstanceUnits="true" boundaryCondition="false" constant="false"/>

<species id="p2127" name="p2127" compartment="rootCompartment" initialAmount="100" hasOnlySubstanceUnits="true" boundaryCondition="false" constant="false"/>

<species id="pp2127" name="pp2127" compartment="rootCompartment" initialAmount="0" hasOnlySubstanceUnits="true" boundaryCondition="false" constant="false"/>

```
    <species id="E2F" name="E2F" compartment="rootCompartment"
initialAmount="0" hasOnlySubstanceUnits="true" boundaryCondition="false"
constant="false"/>
    <species id="RbE2F" name="RbE2F" compartment="rootCompartment"
initialAmount="0" hasOnlySubstanceUnits="true" boundaryCondition="false"
constant="false"/>
    <species id="Rb" name="Rb" compartment="rootCompartment"
initialAmount="100" hasOnlySubstanceUnits="true" boundaryCondition="false"
constant="false"/>
    <species id="pRb" name="pRb" compartment="rootCompartment"
initialAmount="0" hasOnlySubstanceUnits="true" boundaryCondition="false"
constant="false"/>
    <species id="pppRb" name="pppRb" compartment="rootCompartment"
initialAmount="0" hasOnlySubstanceUnits="true" boundaryCondition="false"
constant="false"/>
  </listOfSpecies>
  <listOfParameters>
    <parameter id="kc1f1" name="kc1f1" value="0.1" constant="true"/>
    <parameter id="Vmax1r1" name="Vmax1r1" value="10" constant="true"/>
    <parameter id="kc2f1" name="kc2f1" value="0.1" constant="true"/>
    <parameter id="Ki2" name="Ki2" value="0.1" constant="true"/>
    <parameter id="kc2f2" name="kc2f2" value="0.1" constant="true"/>
    <parameter id="Vmax2r1" name="Vmax2r1" value="10" constant="true"/>
    <parameter id="kc3f1" name="kc3f1" value="0.1" constant="true"/>
    <parameter id="Vmax3r1" name="Vmax3r1" value="10" constant="true"/>
    <parameter id="kc3r1" name="kc3r1" value="0.1" constant="true"/>
    <parameter id="kc4f1" name="kc4f1" value="0.1" constant="true"/>
    <parameter id="Vmax4r1" name="Vmax4r1" value="10" constant="true"/>
    <parameter id="kc5f1" name="kc5f1" value="0.1" constant="true"/>
    <parameter id="Ki5" name="Ki5" value="0.01" constant="true"/>
    <parameter id="Vmax5r1" name="Vmax5r1" value="10" constant="true"/>
```

```xml
<parameter id="kc6f1" name="kc6f1" value="0.1" constant="true"/>
<parameter id="Vmax6r1" name="Vmax6r1" value="10" constant="true"/>
<parameter id="kc7f1" name="kc7f1" value="0.1" constant="true"/>
<parameter id="alpha7" name="alpha7" value="0.01" constant="true"/>
<parameter id="Vmax7r1" name="Vmax7r1" value="10" constant="true"/>
<parameter id="kc8f1" name="kc8f1" value="0.1" constant="true"/>
<parameter id="Vmax8r1" name="Vmax8r1" value="10" constant="true"/>
<parameter id="kc9f1" name="kc9f1" value="0.1" constant="true"/>
<parameter id="Vmax9r1" name="Vmax9r1" value="10" constant="true"/>
<parameter id="kc10f1" name="kc10f1" value="0.1" constant="true"/>
<parameter id="alpha10" name="alpha10" value="0.01" constant="true"/>
<parameter id="Vmax10r1" name="Vmax10r1" value="10" constant="true"/>
<parameter id="kc11f1" name="kc11f1" value="0.1" constant="true"/>
<parameter id="kc11f2" name="kc11f2" value="0.1" constant="true"/>
<parameter id="Ki11" name="Ki11" value="0.01" constant="true"/>
<parameter id="Vmax11r1" name="Vmax11r1" value="10" constant="true"/>
<parameter id="kc12f1" name="kc12f1" value="0.1" constant="true"/>
<parameter id="Ki12" name="Ki12" value="0.01" constant="true"/>
<parameter id="Vmax12r1" name="Vmax12r1" value="10" constant="true"/>
<parameter id="kc13f1" name="kc13f1" value="0.1" constant="true"/>
<parameter id="Vmax13r1" name="Vmax13r1" value="10" constant="true"/>
<parameter id="kc14f1" name="kc14f1" value="0.1" constant="true"/>
<parameter id="Vmax14r1" name="Vmax14r1" value="10" constant="true"/>
<parameter id="kc15f1" name="kc15f1" value="0.1" constant="true"/>
<parameter id="alpha15" name="alpha15" value="0.01" constant="true"/>
<parameter id="Ki15" name="Ki15" value="0.01" constant="true"/>
<parameter id="Vmax15r1" name="Vmax15r1" value="10" constant="true"/>
<parameter id="kc16f1" name="kc16f1" value="0.001" constant="true"/>
```

```xml
<parameter id="alpha16a" name="alpha16a" value="0.01" constant="true"/>
<parameter id="alpha16b" name="alpha16b" value="0.01" constant="true"/>
<parameter id="kc16f2" name="kc16f2" value="0.001" constant="true"/>
<parameter id="kdeg17f1" name="kdeg17f1" value="0.1" constant="true"/>
<parameter id="alpha17" name="alpha17" value="0.1" constant="true"/>
<parameter id="kc18f1" name="kc18f1" value="0.1" constant="true"/>
<parameter id="Vmax18r1" name="Vmax18r1" value="10" constant="true"/>
<parameter id="kc19f1" name="kc19f1" value="0.1" constant="true"/>
<parameter id="Vmax19r1" name="Vmax19r1" value="10" constant="true"/>
<parameter id="kc20f1" name="kc20f1" value="0.1" constant="true"/>
<parameter id="Vmax20r1" name="Vmax20r1" value="10" constant="true"/>
<parameter id="kc21f1" name="kc21f1" value="0.1" constant="true"/>
<parameter id="kc21f2" name="kc21f2" value="0.1" constant="true"/>
<parameter id="kc21f3" name="kc21f3" value="0.1" constant="true"/>
<parameter id="kc21f4" name="kc21f4" value="0.1" constant="true"/>
<parameter id="alpha21a" name="alpha21a" value="0.01" constant="true"/>
<parameter id="alpha21b" name="alpha21b" value="0.01" constant="true"/>
<parameter id="kdeg22f1" name="kdeg22f1" value="1" constant="true"/>
<parameter id="alpha22" name="alpha22" value="0.1" constant="true"/>
<parameter id="kc23f1" name="kc23f1" value="0.1" constant="true"/>
<parameter id="alpha23" name="alpha23" value="0.01" constant="true"/>
<parameter id="kc23f2" name="kc23f2" value="0.1" constant="true"/>
<parameter id="kdeg24f1" name="kdeg24f1" value="0.1" constant="true"/>
<parameter id="alpha24" name="alpha24" value="0.1" constant="true"/>
<parameter id="kc25f1" name="kc25f1" value="0.1" constant="true"/>
<parameter id="alpha25" name="alpha25" value="0.01" constant="true"/>
<parameter id="kc25f2" name="kc25f2" value="0.1" constant="true"/>
<parameter id="kdeg26f1" name="kdeg26f1" value="0.1" constant="true"/>
```

```xml
<parameter id="ka27f1" name="ka27f1" value="0.001" constant="true"/>
<parameter id="alpha27" name="alpha27" value="0.01" constant="true"/>
<parameter id="Ki27" name="Ki27" value="0.01" constant="true"/>
<parameter id="kd27r1" name="kd27r1" value="0.01" constant="true"/>
<parameter id="ka28f1" name="ka28f1" value="0.001" constant="true"/>
<parameter id="Ki28" name="Ki28" value="0.01" constant="true"/>
<parameter id="kd28r1" name="kd28r1" value="0.01" constant="true"/>
<parameter id="ka29f1" name="ka29f1" value="0.001" constant="true"/>
<parameter id="alpha29" name="alpha29" value="0.01" constant="true"/>
<parameter id="kd29r1" name="kd29r1" value="0.01" constant="true"/>
<parameter id="kc30f1" name="kc30f1" value="0.1" constant="true"/>
<parameter id="alpha30" name="alpha30" value="0.01" constant="true"/>
<parameter id="Vmax30r1" name="Vmax30r1" value="10" constant="true"/>
<parameter id="kc31f1" name="kc31f1" value="0.1" constant="true"/>
<parameter id="kc31f2" name="kc31f2" value="0.1" constant="true"/>
<parameter id="kdeg32f1" name="kdeg32f1" value="0.1" constant="true"/>
<parameter id="ka33f1" name="ka33f1" value="0.001" constant="true"/>
<parameter id="kd33r1" name="kd33r1" value="0.01" constant="true"/>
<parameter id="kc34f1" name="kc34f1" value="0.1" constant="true"/>
<parameter id="Vmax34r1" name="Vmax34r1" value="10" constant="true"/>
<parameter id="kc35f1" name="kc35f1" value="0.1" constant="true"/>
<parameter id="Vmax35r1" name="Vmax35r1" value="10" constant="true"/>
<parameter id="PTEN" name="PTEN" value="100" constant="true"/>
<parameter id="AKTscale" name="AKTscale" value="5" constant="true"/>
<parameter id="Ins_0" name="Ins_0" value="100" constant="true"/>
<parameter id="FGF_0" name="FGF_0" value="100" constant="true"/>
<parameter id="Est_0" name="Est_0" value="100" constant="true"/>
<parameter id="Ei" name="Ei" value="10" constant="true"/>
```

```xml
<parameter id="CDK46i" name="CDK46i" value="10" constant="true"/>
<parameter id="Ins_on" name="Ins_on" value="5000" constant="true"/>
<parameter id="FGF_on" name="FGF_on" value="5000" constant="true"/>
<parameter id="Est_on" name="Est_on" value="5000" constant="true"/>
<parameter id="Ei_on" name="Ei_on" value="10000" constant="true"/>
<parameter id="CDK46i_on" name="CDK46i_on" value="10000" constant="true"/>
<parameter id="IR_Total" name="IR_Total" value="0" constant="false"/>
<parameter id="PI3K_Total" name="PI3K_Total" value="0" constant="false"/>
<parameter id="PIP_Total" name="PIP_Total" value="0" constant="false"/>
<parameter id="PDK1_Total" name="PDK1_Total" value="0" constant="false"/>
<parameter id="mTORC2_Total" name="mTORC2_Total" value="0" constant="false"/>
<parameter id="AKT_Total" name="AKT_Total" value="0" constant="false"/>
<parameter id="mTORC1_Total" name="mTORC1_Total" value="0" constant="false"/>
<parameter id="S6K_Total" name="S6K_Total" value="0" constant="false"/>
<parameter id="FGFR_Total" name="FGFR_Total" value="0" constant="false"/>
<parameter id="Ras_Total" name="Ras_Total" value="0" constant="false"/>
<parameter id="Raf_Total" name="Raf_Total" value="0" constant="false"/>
<parameter id="MEK_Total" name="MEK_Total" value="0" constant="false"/>
<parameter id="ERK_Total" name="ERK_Total" value="0" constant="false"/>
<parameter id="ERa_Total" name="ERa_Total" value="0" constant="false"/>
<parameter id="GSK3b_Total" name="GSK3b_Total" value="0" constant="false"/>
<parameter id="FOXO3_Total" name="FOXO3_Total" value="0" constant="false"/>
<parameter id="CDK46_Total" name="CDK46_Total" value="0" constant="false"/>
<parameter id="CDK2_Total" name="CDK2_Total" value="0" constant="false"/>
<parameter id="p2127_Total" name="p2127_Total" value="0" constant="false"/>
<parameter id="Rb_Total" name="Rb_Total" value="0" constant="false"/>
<parameter id="Ins" name="Ins" value="0" constant="false"/>
```

```xml
  <parameter id="FGF" name="FGF" value="0" constant="false"/>
  <parameter id="Est" name="Est" value="0" constant="false"/>
  <parameter id="Ei_0" name="Ei_0" value="0" constant="false"/>
  <parameter id="CDK46i_0" name="CDK46i_0" value="0" constant="false"/>
</listOfParameters>
<listOfRules>
  <assignmentRule variable="IR_Total">
   <math xmlns="http://www.w3.org/1998/Math/MathML">
    <apply>
     <plus/>
     <ci> IR </ci>
     <ci> pIR </ci>
    </apply>
   </math>
  </assignmentRule>
  <assignmentRule variable="PI3K_Total">
   <math xmlns="http://www.w3.org/1998/Math/MathML">
    <apply>
     <plus/>
     <ci> PI3K </ci>
     <ci> pPI3K </ci>
    </apply>
   </math>
  </assignmentRule>
  <assignmentRule variable="PIP_Total">
   <math xmlns="http://www.w3.org/1998/Math/MathML">
    <apply>
     <plus/>
```

```
      <ci> PIP2 </ci>
      <ci> PIP3 </ci>
    </apply>
  </math>
</assignmentRule>
<assignmentRule variable="PDK1_Total">
  <math xmlns="http://www.w3.org/1998/Math/MathML">
    <apply>
      <plus/>
      <ci> PDK1 </ci>
      <ci> aPDK1 </ci>
    </apply>
  </math>
</assignmentRule>
<assignmentRule variable="mTORC2_Total">
  <math xmlns="http://www.w3.org/1998/Math/MathML">
    <apply>
      <plus/>
      <ci> mTORC2 </ci>
      <ci> amTORC2 </ci>
    </apply>
  </math>
</assignmentRule>
<assignmentRule variable="AKT_Total">
  <math xmlns="http://www.w3.org/1998/Math/MathML">
    <apply>
      <plus/>
      <ci> AKT </ci>
```

```
    <ci> pAKT308 </ci>
    <ci> ppAKT308473 </ci>
   </apply>
  </math>
</assignmentRule>
<assignmentRule variable="mTORC1_Total">
  <math xmlns="http://www.w3.org/1998/Math/MathML">
   <apply>
    <plus/>
    <ci> mTORC1 </ci>
    <ci> amTORC1 </ci>
   </apply>
  </math>
</assignmentRule>
<assignmentRule variable="S6K_Total">
  <math xmlns="http://www.w3.org/1998/Math/MathML">
   <apply>
    <plus/>
    <ci> S6K </ci>
    <ci> pS6K </ci>
   </apply>
  </math>
</assignmentRule>
<assignmentRule variable="FGFR_Total">
  <math xmlns="http://www.w3.org/1998/Math/MathML">
   <apply>
    <plus/>
    <ci> FGFR </ci>
```

```
      <ci> pFGFR </ci>
    </apply>
  </math>
</assignmentRule>
<assignmentRule variable="Ras_Total">
  <math xmlns="http://www.w3.org/1998/Math/MathML">
    <apply>
      <plus/>
      <ci> Ras </ci>
      <ci> aRas </ci>
    </apply>
  </math>
</assignmentRule>
<assignmentRule variable="Raf_Total">
  <math xmlns="http://www.w3.org/1998/Math/MathML">
    <apply>
      <plus/>
      <ci> Raf </ci>
      <ci> aRaf </ci>
    </apply>
  </math>
</assignmentRule>
<assignmentRule variable="MEK_Total">
  <math xmlns="http://www.w3.org/1998/Math/MathML">
    <apply>
      <plus/>
      <ci> MEK </ci>
      <ci> pMEK </ci>
```

```
   </apply>
  </math>
</assignmentRule>
<assignmentRule variable="ERK_Total">
  <math xmlns="http://www.w3.org/1998/Math/MathML">
   <apply>
    <plus/>
    <ci> ERK </ci>
    <ci> pERK </ci>
   </apply>
  </math>
</assignmentRule>
<assignmentRule variable="ERa_Total">
  <math xmlns="http://www.w3.org/1998/Math/MathML">
   <apply>
    <plus/>
    <ci> ERa </ci>
    <ci> aERa </ci>
   </apply>
  </math>
</assignmentRule>
<assignmentRule variable="GSK3b_Total">
  <math xmlns="http://www.w3.org/1998/Math/MathML">
   <apply>
    <plus/>
    <ci> GSK3b </ci>
    <ci> pGSK3b </ci>
   </apply>
```

```
  </math>
</assignmentRule>
<assignmentRule variable="FOXO3_Total">
  <math xmlns="http://www.w3.org/1998/Math/MathML">
    <apply>
      <plus/>
      <ci> FOXO3 </ci>
      <ci> pFOXO3 </ci>
    </apply>
  </math>
</assignmentRule>
<assignmentRule variable="CDK46_Total">
  <math xmlns="http://www.w3.org/1998/Math/MathML">
    <apply>
      <plus/>
      <ci> CDK46 </ci>
      <ci> CDK46cycD </ci>
    </apply>
  </math>
</assignmentRule>
<assignmentRule variable="CDK2_Total">
  <math xmlns="http://www.w3.org/1998/Math/MathML">
    <apply>
      <plus/>
      <ci> CDK2 </ci>
      <ci> CDK2cycE </ci>
      <ci> CDK2cycA </ci>
    </apply>
```

```
  </math>
</assignmentRule>
<assignmentRule variable="p2127_Total">
  <math xmlns="http://www.w3.org/1998/Math/MathML">
    <apply>
      <plus/>
      <ci> p2127 </ci>
      <ci> pp2127 </ci>
    </apply>
  </math>
</assignmentRule>
<assignmentRule variable="Rb_Total">
  <math xmlns="http://www.w3.org/1998/Math/MathML">
    <apply>
      <plus/>
      <ci> Rb </ci>
      <ci> pRb </ci>
      <ci> pppRb </ci>
    </apply>
  </math>
</assignmentRule>
<assignmentRule variable="Ins">
  <math xmlns="http://www.w3.org/1998/Math/MathML">
    <apply>
      <times/>
      <ci> Ins_0 </ci>
      <piecewise>
        <piece>
```

```
            <cn type="integer"> 1 </cn>
            <apply>
             <geq/>
             <csymbol encoding="text"
definitionURL="http://www.sbml.org/sbml/symbols/time"> time </csymbol>
             <ci> Ins_on </ci>
            </apply>
           </piece>
           <otherwise>
            <cn type="integer"> 0 </cn>
           </otherwise>
          </piecewise>
         </apply>
        </math>
       </assignmentRule>
       <assignmentRule variable="FGF">
        <math xmlns="http://www.w3.org/1998/Math/MathML">
         <apply>
          <times/>
          <ci> FGF_0 </ci>
          <piecewise>
           <piece>
            <cn type="integer"> 1 </cn>
            <apply>
             <geq/>
             <csymbol encoding="text"
definitionURL="http://www.sbml.org/sbml/symbols/time"> time </csymbol>
             <ci> FGF_on </ci>
            </apply>
```

```
       </piece>
       <otherwise>
        <cn type="integer"> 0 </cn>
       </otherwise>
      </piecewise>
     </apply>
    </math>
   </assignmentRule>
   <assignmentRule variable="Est">
    <math xmlns="http://www.w3.org/1998/Math/MathML">
     <apply>
      <times/>
      <ci> Est_0 </ci>
      <piecewise>
       <piece>
        <cn type="integer"> 1 </cn>
        <apply>
         <geq/>
         <csymbol encoding="text"
definitionURL="http://www.sbml.org/sbml/symbols/time"> time </csymbol>
         <ci> Est_on </ci>
        </apply>
       </piece>
       <otherwise>
        <cn type="integer"> 0 </cn>
       </otherwise>
      </piecewise>
     </apply>
```

```
  </math>
 </assignmentRule>
 <assignmentRule variable="Ei_0">
  <math xmlns="http://www.w3.org/1998/Math/MathML">
   <apply>
    <times/>
    <ci> Ei </ci>
    <piecewise>
     <piece>
      <cn type="integer"> 1 </cn>
      <apply>
       <geq/>
       <csymbol encoding="text"
definitionURL="http://www.sbml.org/sbml/symbols/time"> time </csymbol>
       <ci> Ei_on </ci>
      </apply>
     </piece>
     <otherwise>
      <cn type="integer"> 0 </cn>
     </otherwise>
    </piecewise>
   </apply>
  </math>
 </assignmentRule>
 <assignmentRule variable="CDK46i_0">
  <math xmlns="http://www.w3.org/1998/Math/MathML">
   <apply>
    <times/>
```

```
          <ci> CDK46i </ci>
          <piecewise>
            <piece>
              <cn type="integer"> 1 </cn>
              <apply>
                <geq/>
                <csymbol encoding="text"
definitionURL="http://www.sbml.org/sbml/symbols/time"> time </csymbol>
                <ci> CDK46i_on </ci>
              </apply>
            </piece>
            <otherwise>
              <cn type="integer"> 0 </cn>
            </otherwise>
          </piecewise>
        </apply>
      </math>
    </assignmentRule>
  </listOfRules>
  <listOfReactions>
    <reaction id="R1" name="R1" reversible="true">
      <listOfReactants>
        <speciesReference species="IR" stoichiometry="1"/>
      </listOfReactants>
      <listOfProducts>
        <speciesReference species="pIR" stoichiometry="1"/>
      </listOfProducts>
      <kineticLaw>
```

```
    <math xmlns="http://www.w3.org/1998/Math/MathML">
     <apply>
      <minus/>
      <apply>
       <times/>
       <ci> kc1f1 </ci>
       <ci> Ins </ci>
       <ci> IR </ci>
      </apply>
      <apply>
       <times/>
       <ci> Vmax1r1 </ci>
       <ci> pIR </ci>
      </apply>
     </apply>
    </math>
   </kineticLaw>
  </reaction>
  <reaction id="R2" name="R2" reversible="true">
   <listOfReactants>
    <speciesReference species="PI3K" stoichiometry="1"/>
   </listOfReactants>
   <listOfProducts>
    <speciesReference species="pPI3K" stoichiometry="1"/>
   </listOfProducts>
   <listOfModifiers>
    <modifierSpeciesReference species="pFGFR"/>
    <modifierSpeciesReference species="pIR"/>
```

```
  <modifierSpeciesReference species="pS6K"/>
</listOfModifiers>
<kineticLaw>
  <math xmlns="http://www.w3.org/1998/Math/MathML">
    <apply>
      <minus/>
      <apply>
        <plus/>
        <apply>
          <divide/>
          <apply>
            <times/>
            <ci> kc2f1 </ci>
            <ci> pIR </ci>
            <ci> PI3K </ci>
          </apply>
          <apply>
            <plus/>
            <cn type="integer"> 1 </cn>
            <apply>
              <divide/>
              <ci> pS6K </ci>
              <ci> Ki2 </ci>
            </apply>
          </apply>
        </apply>
        <apply>
          <times/>
```

```
          &lt;ci&gt; kc2f2 &lt;/ci&gt;
          &lt;ci&gt; pFGFR &lt;/ci&gt;
          &lt;ci&gt; PI3K &lt;/ci&gt;
        &lt;/apply&gt;
      &lt;/apply&gt;
      &lt;apply&gt;
        &lt;times/&gt;
        &lt;ci&gt; Vmax2r1 &lt;/ci&gt;
        &lt;ci&gt; pPI3K &lt;/ci&gt;
      &lt;/apply&gt;
    &lt;/apply&gt;
  &lt;/math&gt;
 &lt;/kineticLaw&gt;
&lt;/reaction&gt;
&lt;reaction id="R3" name="R3" reversible="true"&gt;
 &lt;listOfReactants&gt;
  &lt;speciesReference species="PIP2" stoichiometry="1"/&gt;
 &lt;/listOfReactants&gt;
 &lt;listOfProducts&gt;
  &lt;speciesReference species="PIP3" stoichiometry="1"/&gt;
 &lt;/listOfProducts&gt;
 &lt;listOfModifiers&gt;
  &lt;modifierSpeciesReference species="pPI3K"/&gt;
 &lt;/listOfModifiers&gt;
 &lt;kineticLaw&gt;
  &lt;math xmlns="http://www.w3.org/1998/Math/MathML"&gt;
   &lt;apply&gt;
    &lt;minus/&gt;
```

```
        <apply>
         <times/>
         <ci> kc3f1 </ci>
         <ci> pPI3K </ci>
         <ci> PIP2 </ci>
        </apply>
        <apply>
         <times/>
         <apply>
          <plus/>
          <ci> Vmax3r1 </ci>
          <apply>
           <times/>
           <ci> kc3r1 </ci>
           <ci> PTEN </ci>
          </apply>
         </apply>
         <ci> PIP3 </ci>
        </apply>
       </apply>
      </math>
     </kineticLaw>
    </reaction>
    <reaction id="R4" name="R4" reversible="true">
     <listOfReactants>
      <speciesReference species="PDK1" stoichiometry="1"/>
     </listOfReactants>
     <listOfProducts>
```

```
    <speciesReference species="aPDK1" stoichiometry="1"/>
   </listOfProducts>
   <listOfModifiers>
    <modifierSpeciesReference species="PIP3"/>
   </listOfModifiers>
   <kineticLaw>
    <math xmlns="http://www.w3.org/1998/Math/MathML">
     <apply>
      <minus/>
      <apply>
       <times/>
       <ci> kc4f1 </ci>
       <ci> PIP3 </ci>
       <ci> PDK1 </ci>
      </apply>
      <apply>
       <times/>
       <ci> Vmax4r1 </ci>
       <ci> aPDK1 </ci>
      </apply>
     </apply>
    </math>
   </kineticLaw>
  </reaction>
  <reaction id="R5" name="R5" reversible="true">
   <listOfReactants>
    <speciesReference species="mTORC2" stoichiometry="1"/>
   </listOfReactants>
```

```
<listOfProducts>
 <speciesReference species="amTORC2" stoichiometry="1"/>
</listOfProducts>
<listOfModifiers>
 <modifierSpeciesReference species="PIP3"/>
 <modifierSpeciesReference species="pppRb"/>
</listOfModifiers>
<kineticLaw>
 <math xmlns="http://www.w3.org/1998/Math/MathML">
  <apply>
   <minus/>
   <apply>
    <divide/>
    <apply>
     <times/>
     <ci> kc5f1 </ci>
     <ci> PIP3 </ci>
     <ci> mTORC2 </ci>
    </apply>
    <apply>
     <plus/>
     <cn type="integer"> 1 </cn>
     <apply>
      <divide/>
      <ci> pppRb </ci>
      <ci> Ki5 </ci>
     </apply>
    </apply>
```

```
        </apply>
        <apply>
          <times/>
          <ci> Vmax5r1 </ci>
          <ci> amTORC2 </ci>
        </apply>
      </apply>
    </math>
  </kineticLaw>
</reaction>
<reaction id="R6" name="R6" reversible="true">
  <listOfReactants>
    <speciesReference species="AKT" stoichiometry="1"/>
  </listOfReactants>
  <listOfProducts>
    <speciesReference species="pAKT308" stoichiometry="1"/>
  </listOfProducts>
  <listOfModifiers>
    <modifierSpeciesReference species="aPDK1"/>
  </listOfModifiers>
  <kineticLaw>
    <math xmlns="http://www.w3.org/1998/Math/MathML">
      <apply>
        <minus/>
        <apply>
          <times/>
          <ci> kc6f1 </ci>
          <ci> aPDK1 </ci>
```

```
        <ci> AKT </ci>
       </apply>
       <apply>
        <times/>
        <ci> Vmax6r1 </ci>
        <ci> pAKT308 </ci>
       </apply>
      </apply>
     </math>
    </kineticLaw>
   </reaction>
   <reaction id="R7" name="R7" reversible="true">
    <listOfReactants>
     <speciesReference species="pAKT308" stoichiometry="1"/>
    </listOfReactants>
    <listOfProducts>
     <speciesReference species="ppAKT308473" stoichiometry="1"/>
    </listOfProducts>
    <listOfModifiers>
     <modifierSpeciesReference species="CDK2cycA"/>
     <modifierSpeciesReference species="amTORC2"/>
    </listOfModifiers>
    <kineticLaw>
     <math xmlns="http://www.w3.org/1998/Math/MathML">
      <apply>
       <minus/>
       <apply>
        <times/>
```

```
     <ci> kc7f1 </ci>
     <ci> amTORC2 </ci>
     <ci> pAKT308 </ci>
     <apply>
      <plus/>
      <cn type="integer"> 1 </cn>
      <apply>
       <times/>
       <ci> alpha7 </ci>
       <ci> CDK2cycA </ci>
      </apply>
     </apply>
    </apply>
    <apply>
     <times/>
     <ci> Vmax7r1 </ci>
     <ci> ppAKT308473 </ci>
    </apply>
   </apply>
  </math>
 </kineticLaw>
</reaction>
<reaction id="R8" name="R8" reversible="true">
 <listOfReactants>
  <speciesReference species="mTORC1" stoichiometry="1"/>
 </listOfReactants>
 <listOfProducts>
  <speciesReference species="amTORC1" stoichiometry="1"/>
```

```xml
&lt;/listOfProducts&gt;
&lt;listOfModifiers&gt;
 &lt;modifierSpeciesReference species="pAKT308"/&gt;
 &lt;modifierSpeciesReference species="ppAKT308473"/&gt;
&lt;/listOfModifiers&gt;
&lt;kineticLaw&gt;
 &lt;math xmlns="http://www.w3.org/1998/Math/MathML"&gt;
  &lt;apply&gt;
   &lt;minus/&gt;
   &lt;apply&gt;
    &lt;times/&gt;
    &lt;apply&gt;
     &lt;plus/&gt;
     &lt;apply&gt;
      &lt;times/&gt;
      &lt;ci&gt; kc8f1 &lt;/ci&gt;
      &lt;ci&gt; pAKT308 &lt;/ci&gt;
     &lt;/apply&gt;
     &lt;apply&gt;
      &lt;times/&gt;
      &lt;ci&gt; AKTscale &lt;/ci&gt;
      &lt;ci&gt; kc8f1 &lt;/ci&gt;
      &lt;ci&gt; ppAKT308473 &lt;/ci&gt;
     &lt;/apply&gt;
    &lt;/apply&gt;
    &lt;ci&gt; mTORC1 &lt;/ci&gt;
   &lt;/apply&gt;
   &lt;apply&gt;
```

```
          <times/>
          <ci> Vmax8r1 </ci>
          <ci> amTORC1 </ci>
        </apply>
      </apply>
    </math>
  </kineticLaw>
</reaction>
<reaction id="R9" name="R9" reversible="true">
  <listOfReactants>
    <speciesReference species="S6K" stoichiometry="1"/>
  </listOfReactants>
  <listOfProducts>
    <speciesReference species="pS6K" stoichiometry="1"/>
  </listOfProducts>
  <listOfModifiers>
    <modifierSpeciesReference species="amTORC1"/>
  </listOfModifiers>
  <kineticLaw>
    <math xmlns="http://www.w3.org/1998/Math/MathML">
      <apply>
        <minus/>
        <apply>
          <times/>
          <ci> kc9f1 </ci>
          <ci> amTORC1 </ci>
          <ci> S6K </ci>
        </apply>
```

```
      <apply>
       <times/>
       <ci> Vmax9r1 </ci>
       <ci> pS6K </ci>
      </apply>
     </apply>
    </math>
   </kineticLaw>
  </reaction>
  <reaction id="R10" name="R10" reversible="true">
   <listOfReactants>
    <speciesReference species="FGFR" stoichiometry="1"/>
   </listOfReactants>
   <listOfProducts>
    <speciesReference species="pFGFR" stoichiometry="1"/>
   </listOfProducts>
   <listOfModifiers>
    <modifierSpeciesReference species="E2F"/>
   </listOfModifiers>
   <kineticLaw>
    <math xmlns="http://www.w3.org/1998/Math/MathML">
     <apply>
      <minus/>
      <apply>
       <times/>
       <ci> kc10f1 </ci>
       <ci> FGF </ci>
       <ci> FGFR </ci>
```

```
      <apply>
       <plus/>
       <cn type="integer"> 1 </cn>
       <apply>
        <times/>
        <ci> alpha10 </ci>
        <ci> E2F </ci>
       </apply>
      </apply>
     </apply>
     <apply>
      <times/>
      <ci> Vmax10r1 </ci>
      <ci> pFGFR </ci>
     </apply>
    </apply>
   </math>
  </kineticLaw>
</reaction>
<reaction id="R11" name="R11" reversible="true">
  <listOfReactants>
   <speciesReference species="Ras" stoichiometry="1"/>
  </listOfReactants>
  <listOfProducts>
   <speciesReference species="aRas" stoichiometry="1"/>
  </listOfProducts>
  <listOfModifiers>
   <modifierSpeciesReference species="pERK"/>
```

```
  <modifierSpeciesReference species="pFGFR"/>
  <modifierSpeciesReference species="pIR"/>
 </listOfModifiers>
 <kineticLaw>
  <math xmlns="http://www.w3.org/1998/Math/MathML">
   <apply>
    <minus/>
    <apply>
     <divide/>
     <apply>
      <times/>
      <apply>
       <plus/>
       <apply>
        <times/>
        <ci> kc11f1 </ci>
        <ci> pFGFR </ci>
       </apply>
       <apply>
        <times/>
        <ci> kc11f2 </ci>
        <ci> pIR </ci>
       </apply>
      </apply>
      <ci> Ras </ci>
     </apply>
     <apply>
      <plus/>
```

```
        <cn type="integer"> 1 </cn>
        <apply>
         <divide/>
         <ci> pERK </ci>
         <ci> Ki11 </ci>
        </apply>
       </apply>
      </apply>
      <apply>
       <times/>
       <ci> Vmax11r1 </ci>
       <ci> aRas </ci>
      </apply>
     </apply>
    </math>
   </kineticLaw>
  </reaction>
  <reaction id="R12" name="R12" reversible="true">
   <listOfReactants>
    <speciesReference species="Raf" stoichiometry="1"/>
   </listOfReactants>
   <listOfProducts>
    <speciesReference species="aRaf" stoichiometry="1"/>
   </listOfProducts>
   <listOfModifiers>
    <modifierSpeciesReference species="aRas"/>
    <modifierSpeciesReference species="pAKT308"/>
    <modifierSpeciesReference species="ppAKT308473"/>
```

```
</listOfModifiers>
<kineticLaw>
 <math xmlns="http://www.w3.org/1998/Math/MathML">
  <apply>
   <minus/>
   <apply>
    <divide/>
    <apply>
     <times/>
     <ci> kc12f1 </ci>
     <ci> aRas </ci>
     <ci> Raf </ci>
    </apply>
    <apply>
     <plus/>
     <cn type="integer"> 1 </cn>
     <apply>
      <divide/>
      <ci> pAKT308 </ci>
      <ci> Ki12 </ci>
     </apply>
     <apply>
      <divide/>
      <ci> ppAKT308473 </ci>
      <apply>
       <times/>
       <ci> AKTscale </ci>
       <ci> Ki12 </ci>
```

```
          </apply>
         </apply>
        </apply>
       </apply>
       <apply>
        <times/>
        <ci> Vmax12r1 </ci>
        <ci> aRaf </ci>
       </apply>
      </apply>
     </math>
    </kineticLaw>
   </reaction>
   <reaction id="R13" name="R13" reversible="true">
    <listOfReactants>
     <speciesReference species="MEK" stoichiometry="1"/>
    </listOfReactants>
    <listOfProducts>
     <speciesReference species="pMEK" stoichiometry="1"/>
    </listOfProducts>
    <listOfModifiers>
     <modifierSpeciesReference species="aRaf"/>
    </listOfModifiers>
    <kineticLaw>
     <math xmlns="http://www.w3.org/1998/Math/MathML">
      <apply>
       <minus/>
       <apply>
```

```
        <times/>
        <ci> kc13f1 </ci>
        <ci> aRaf </ci>
        <ci> MEK </ci>
       </apply>
       <apply>
        <times/>
        <ci> Vmax13r1 </ci>
        <ci> pMEK </ci>
       </apply>
      </apply>
     </math>
    </kineticLaw>
   </reaction>
   <reaction id="R14" name="R14" reversible="true">
    <listOfReactants>
     <speciesReference species="ERK" stoichiometry="1"/>
    </listOfReactants>
    <listOfProducts>
     <speciesReference species="pERK" stoichiometry="1"/>
    </listOfProducts>
    <listOfModifiers>
     <modifierSpeciesReference species="pMEK"/>
    </listOfModifiers>
    <kineticLaw>
     <math xmlns="http://www.w3.org/1998/Math/MathML">
      <apply>
       <minus/>
```

```
        <apply>
         <times/>
         <ci> kc14f1 </ci>
         <ci> pMEK </ci>
         <ci> ERK </ci>
        </apply>
        <apply>
         <times/>
         <ci> Vmax14r1 </ci>
         <ci> pERK </ci>
        </apply>
       </apply>
      </math>
    </kineticLaw>
   </reaction>
   <reaction id="R15" name="R15" reversible="true">
    <listOfReactants>
     <speciesReference species="ERa" stoichiometry="1"/>
    </listOfReactants>
    <listOfProducts>
     <speciesReference species="aERa" stoichiometry="1"/>
    </listOfProducts>
    <listOfModifiers>
     <modifierSpeciesReference species="FOXO3"/>
    </listOfModifiers>
    <kineticLaw>
     <math xmlns="http://www.w3.org/1998/Math/MathML">
       <apply>
```

```
<minus/>
<apply>
  <divide/>
  <apply>
    <times/>
    <ci> kc15f1 </ci>
    <ci> Est </ci>
    <ci> ERa </ci>
    <apply>
      <plus/>
      <cn type="integer"> 1 </cn>
      <apply>
        <times/>
        <ci> alpha15 </ci>
        <ci> FOXO3 </ci>
      </apply>
    </apply>
  </apply>
  <apply>
    <plus/>
    <cn type="integer"> 1 </cn>
    <apply>
      <divide/>
      <ci> Ei_0 </ci>
      <ci> Ki15 </ci>
    </apply>
  </apply>
</apply>
```

```
      <apply>
       <times/>
       <ci> Vmax15r1 </ci>
       <ci> aERa </ci>
      </apply>
     </apply>
    </math>
   </kineticLaw>
  </reaction>
  <reaction id="R16" name="R16" reversible="false">
   <listOfProducts>
    <speciesReference species="Myc" stoichiometry="1"/>
   </listOfProducts>
   <listOfModifiers>
    <modifierSpeciesReference species="E2F"/>
    <modifierSpeciesReference species="aERa"/>
    <modifierSpeciesReference species="cycD"/>
    <modifierSpeciesReference species="pERK"/>
   </listOfModifiers>
   <kineticLaw>
    <math xmlns="http://www.w3.org/1998/Math/MathML">
     <apply>
      <plus/>
      <apply>
       <times/>
       <ci> kc16f1 </ci>
       <ci> aERa </ci>
       <apply>
```

```
          <plus/>
          <cn type="integer"> 1 </cn>
          <apply>
            <times/>
            <ci> alpha16a </ci>
            <ci> pERK </ci>
          </apply>
          <apply>
            <times/>
            <ci> alpha16b </ci>
            <ci> cycD </ci>
          </apply>
        </apply>
      </apply>
      <apply>
        <times/>
        <ci> kc16f2 </ci>
        <ci> E2F </ci>
      </apply>
    </apply>
  </math>
 </kineticLaw>
</reaction>
<reaction id="R17" name="R17" reversible="false">
 <listOfReactants>
  <speciesReference species="Myc" stoichiometry="1"/>
 </listOfReactants>
 <listOfModifiers>
```

```
    <modifierSpeciesReference species="GSK3b"/>
  </listOfModifiers>
  <kineticLaw>
    <math xmlns="http://www.w3.org/1998/Math/MathML">
      <apply>
        <times/>
        <ci> kdeg17f1 </ci>
        <ci> Myc </ci>
        <apply>
          <plus/>
          <cn type="integer"> 1 </cn>
          <apply>
            <times/>
            <ci> alpha17 </ci>
            <ci> GSK3b </ci>
          </apply>
        </apply>
      </apply>
    </math>
  </kineticLaw>
</reaction>
<reaction id="R18" name="R18" reversible="true">
  <listOfReactants>
    <speciesReference species="Myc" stoichiometry="1"/>
  </listOfReactants>
  <listOfProducts>
    <speciesReference species="pMyc" stoichiometry="1"/>
  </listOfProducts>
```

```
  <listOfModifiers>
    <modifierSpeciesReference species="pERK"/>
  </listOfModifiers>
  <kineticLaw>
    <math xmlns="http://www.w3.org/1998/Math/MathML">
      <apply>
        <minus/>
        <apply>
          <times/>
          <ci> kc18f1 </ci>
          <ci> pERK </ci>
          <ci> Myc </ci>
        </apply>
        <apply>
          <times/>
          <ci> Vmax18r1 </ci>
          <ci> pMyc </ci>
        </apply>
      </apply>
    </math>
  </kineticLaw>
</reaction>
<reaction id="R19" name="R19" reversible="true">
  <listOfReactants>
    <speciesReference species="GSK3b" stoichiometry="1"/>
  </listOfReactants>
  <listOfProducts>
    <speciesReference species="pGSK3b" stoichiometry="1"/>
```

```
</listOfProducts>
<listOfModifiers>
 <modifierSpeciesReference species="pAKT308"/>
 <modifierSpeciesReference species="ppAKT308473"/>
</listOfModifiers>
<kineticLaw>
 <math xmlns="http://www.w3.org/1998/Math/MathML">
  <apply>
   <minus/>
   <apply>
    <times/>
    <apply>
     <plus/>
     <apply>
      <times/>
      <ci> kc19f1 </ci>
      <ci> pAKT308 </ci>
     </apply>
     <apply>
      <times/>
      <ci> AKTscale </ci>
      <ci> kc19f1 </ci>
      <ci> ppAKT308473 </ci>
     </apply>
    </apply>
    <ci> GSK3b </ci>
   </apply>
   <apply>
```

```
          <times/>
          <ci> Vmax19r1 </ci>
          <ci> pGSK3b </ci>
        </apply>
      </apply>
    </math>
  </kineticLaw>
</reaction>
<reaction id="R20" name="R20" reversible="true">
  <listOfReactants>
    <speciesReference species="FOXO3" stoichiometry="1"/>
  </listOfReactants>
  <listOfProducts>
    <speciesReference species="pFOXO3" stoichiometry="1"/>
  </listOfProducts>
  <listOfModifiers>
    <modifierSpeciesReference species="pAKT308"/>
    <modifierSpeciesReference species="ppAKT308473"/>
  </listOfModifiers>
  <kineticLaw>
    <math xmlns="http://www.w3.org/1998/Math/MathML">
      <apply>
        <minus/>
        <apply>
          <times/>
          <apply>
            <plus/>
            <apply>
```

```
          <times/>
          <ci> kc20f1 </ci>
          <ci> pAKT308 </ci>
         </apply>
         <apply>
          <times/>
          <ci> AKTscale </ci>
          <ci> kc20f1 </ci>
          <ci> ppAKT308473 </ci>
         </apply>
        </apply>
        <ci> FOXO3 </ci>
       </apply>
       <apply>
        <times/>
        <ci> Vmax20r1 </ci>
        <ci> pFOXO3 </ci>
       </apply>
      </apply>
     </math>
    </kineticLaw>
   </reaction>
   <reaction id="R21" name="R21" reversible="false">
    <listOfProducts>
     <speciesReference species="cycD" stoichiometry="1"/>
    </listOfProducts>
    <listOfModifiers>
     <modifierSpeciesReference species="Myc"/>
```

```
  <modifierSpeciesReference species="aERa"/>
  <modifierSpeciesReference species="amTORC1"/>
  <modifierSpeciesReference species="pERK"/>
  <modifierSpeciesReference species="pMyc"/>
</listOfModifiers>
<kineticLaw>
  <math xmlns="http://www.w3.org/1998/Math/MathML">
    <apply>
      <plus/>
      <apply>
        <times/>
        <ci> kc21f1 </ci>
        <ci> amTORC1 </ci>
      </apply>
      <apply>
        <times/>
        <ci> kc21f2 </ci>
        <ci> pERK </ci>
      </apply>
      <apply>
        <times/>
        <ci> kc21f3 </ci>
        <apply>
          <plus/>
          <ci> Myc </ci>
          <ci> pMyc </ci>
        </apply>
      </apply>
```

```
      <apply>
       <times/>
       <ci> kc21f4 </ci>
       <ci> aERa </ci>
       <apply>
        <plus/>
        <cn type="integer"> 1 </cn>
        <apply>
         <times/>
         <ci> alpha21a </ci>
         <ci> pERK </ci>
        </apply>
        <apply>
         <times/>
         <ci> alpha21b </ci>
         <ci> cycD </ci>
        </apply>
       </apply>
      </apply>
     </apply>
    </math>
   </kineticLaw>
  </reaction>
  <reaction id="R22" name="R22" reversible="false">
   <listOfReactants>
    <speciesReference species="cycD" stoichiometry="1"/>
   </listOfReactants>
   <listOfModifiers>
```

```xml
    <modifierSpeciesReference species="GSK3b"/>
  </listOfModifiers>
  <kineticLaw>
    <math xmlns="http://www.w3.org/1998/Math/MathML">
      <apply>
        <times/>
        <ci> kdeg22f1 </ci>
        <ci> cycD </ci>
        <apply>
          <plus/>
          <cn type="integer"> 1 </cn>
          <apply>
            <times/>
            <ci> alpha22 </ci>
            <ci> GSK3b </ci>
          </apply>
        </apply>
      </apply>
    </math>
  </kineticLaw>
</reaction>
<reaction id="R23" name="R23" reversible="false">
  <listOfProducts>
    <speciesReference species="cycE" stoichiometry="1"/>
  </listOfProducts>
  <listOfModifiers>
    <modifierSpeciesReference species="E2F"/>
    <modifierSpeciesReference species="Myc"/>
```

```xml
  <modifierSpeciesReference species="pMyc"/>
</listOfModifiers>
<kineticLaw>
  <math xmlns="http://www.w3.org/1998/Math/MathML">
    <apply>
      <plus/>
      <apply>
        <times/>
        <ci> kc23f1 </ci>
        <ci> E2F </ci>
        <apply>
          <plus/>
          <cn type="integer"> 1 </cn>
          <apply>
            <times/>
            <ci> alpha23 </ci>
            <apply>
              <plus/>
              <ci> Myc </ci>
              <ci> pMyc </ci>
            </apply>
          </apply>
        </apply>
      </apply>
      <apply>
        <times/>
        <ci> kc23f2 </ci>
        <apply>
```

```
          <plus/>
          <ci> Myc </ci>
          <ci> pMyc </ci>
        </apply>
      </apply>
    </apply>
  </math>
 </kineticLaw>
</reaction>
<reaction id="R24" name="R24" reversible="false">
 <listOfReactants>
  <speciesReference species="cycE" stoichiometry="1"/>
 </listOfReactants>
 <listOfModifiers>
  <modifierSpeciesReference species="GSK3b"/>
 </listOfModifiers>
 <kineticLaw>
  <math xmlns="http://www.w3.org/1998/Math/MathML">
   <apply>
    <times/>
    <ci> kdeg24f1 </ci>
    <ci> cycE </ci>
    <apply>
     <plus/>
     <cn type="integer"> 1 </cn>
     <apply>
      <times/>
      <ci> alpha24 </ci>
```

```
        <ci> GSK3b </ci>
       </apply>
      </apply>
     </apply>
    </math>
   </kineticLaw>
  </reaction>
  <reaction id="R25" name="R25" reversible="false">
   <listOfProducts>
    <speciesReference species="cycA" stoichiometry="1"/>
   </listOfProducts>
   <listOfModifiers>
    <modifierSpeciesReference species="E2F"/>
    <modifierSpeciesReference species="Myc"/>
    <modifierSpeciesReference species="pMyc"/>
   </listOfModifiers>
   <kineticLaw>
    <math xmlns="http://www.w3.org/1998/Math/MathML">
     <apply>
      <plus/>
      <apply>
       <times/>
       <ci> kc25f1 </ci>
       <ci> E2F </ci>
       <apply>
        <plus/>
        <cn type="integer"> 1 </cn>
        <apply>
```

```
          <times/>
          <ci> alpha25 </ci>
          <apply>
           <plus/>
           <ci> Myc </ci>
           <ci> pMyc </ci>
          </apply>
         </apply>
        </apply>
       </apply>
       <apply>
        <times/>
        <ci> kc25f2 </ci>
        <apply>
         <plus/>
         <ci> Myc </ci>
         <ci> pMyc </ci>
        </apply>
       </apply>
      </apply>
     </math>
    </kineticLaw>
   </reaction>
   <reaction id="R26" name="R26" reversible="false">
    <listOfReactants>
     <speciesReference species="cycA" stoichiometry="1"/>
    </listOfReactants>
    <kineticLaw>
```

```
    <math xmlns="http://www.w3.org/1998/Math/MathML">
      <apply>
        <times/>
        <ci> kdeg26f1 </ci>
        <ci> cycA </ci>
      </apply>
    </math>
  </kineticLaw>
</reaction>
<reaction id="R27" name="R27" reversible="true">
  <listOfReactants>
    <speciesReference species="cycD" stoichiometry="1"/>
    <speciesReference species="CDK46" stoichiometry="1"/>
  </listOfReactants>
  <listOfProducts>
    <speciesReference species="CDK46cycD" stoichiometry="1"/>
  </listOfProducts>
  <listOfModifiers>
    <modifierSpeciesReference species="pp2127"/>
  </listOfModifiers>
  <kineticLaw>
    <math xmlns="http://www.w3.org/1998/Math/MathML">
      <apply>
        <minus/>
        <apply>
          <divide/>
          <apply>
            <times/>
```

```
    <ci> ka27f1 </ci>
    <ci> CDK46 </ci>
    <ci> cycD </ci>
    <apply>
     <plus/>
     <cn type="integer"> 1 </cn>
     <apply>
      <times/>
      <ci> alpha27 </ci>
      <ci> pp2127 </ci>
     </apply>
    </apply>
   </apply>
   <apply>
    <plus/>
    <cn type="integer"> 1 </cn>
    <apply>
     <divide/>
     <ci> CDK46i_0 </ci>
     <ci> Ki27 </ci>
    </apply>
   </apply>
  </apply>
  <apply>
   <times/>
   <ci> kd27r1 </ci>
   <ci> CDK46cycD </ci>
  </apply>
```

```
    </apply>
   </math>
  </kineticLaw>
</reaction>
<reaction id="R28" name="R28" reversible="true">
  <listOfReactants>
   <speciesReference species="cycE" stoichiometry="1"/>
   <speciesReference species="CDK2" stoichiometry="1"/>
  </listOfReactants>
  <listOfProducts>
   <speciesReference species="CDK2cycE" stoichiometry="1"/>
  </listOfProducts>
  <listOfModifiers>
   <modifierSpeciesReference species="p2127"/>
  </listOfModifiers>
  <kineticLaw>
   <math xmlns="http://www.w3.org/1998/Math/MathML">
    <apply>
     <minus/>
     <apply>
      <divide/>
      <apply>
       <times/>
       <ci> ka28f1 </ci>
       <ci> CDK2 </ci>
       <ci> cycE </ci>
      </apply>
      <apply>
```

```
          <plus/>
          <cn type="integer"> 1 </cn>
          <apply>
            <divide/>
            <ci> p2127 </ci>
            <ci> Ki28 </ci>
          </apply>
        </apply>
      </apply>
      <apply>
        <times/>
        <ci> kd28r1 </ci>
        <ci> CDK2cycE </ci>
      </apply>
    </apply>
  </math>
 </kineticLaw>
</reaction>
<reaction id="R29" name="R29" reversible="true">
 <listOfReactants>
  <speciesReference species="cycA" stoichiometry="1"/>
  <speciesReference species="CDK2" stoichiometry="1"/>
 </listOfReactants>
 <listOfProducts>
  <speciesReference species="CDK2cycA" stoichiometry="1"/>
 </listOfProducts>
 <listOfModifiers>
  <modifierSpeciesReference species="pAKT308"/>
```

```
  <modifierSpeciesReference species="ppAKT308473"/>
</listOfModifiers>
<kineticLaw>
  <math xmlns="http://www.w3.org/1998/Math/MathML">
    <apply>
      <minus/>
      <apply>
        <times/>
        <ci> ka29f1 </ci>
        <ci> CDK2 </ci>
        <ci> cycA </ci>
        <apply>
          <plus/>
          <cn type="integer"> 1 </cn>
          <apply>
            <times/>
            <ci> alpha29 </ci>
            <ci> pAKT308 </ci>
          </apply>
          <apply>
            <times/>
            <ci> AKTscale </ci>
            <ci> alpha29 </ci>
            <ci> ppAKT308473 </ci>
          </apply>
        </apply>
      </apply>
      <apply>
```

```
        <times/>
        <ci> kd29r1 </ci>
        <ci> CDK2cycA </ci>
       </apply>
      </apply>
     </math>
    </kineticLaw>
   </reaction>
   <reaction id="R30" name="R30" reversible="true">
    <listOfReactants>
     <speciesReference species="p2127" stoichiometry="1"/>
    </listOfReactants>
    <listOfProducts>
     <speciesReference species="pp2127" stoichiometry="1"/>
    </listOfProducts>
    <listOfModifiers>
     <modifierSpeciesReference species="Myc"/>
     <modifierSpeciesReference species="pAKT308"/>
     <modifierSpeciesReference species="pMyc"/>
     <modifierSpeciesReference species="ppAKT308473"/>
    </listOfModifiers>
    <kineticLaw>
     <math xmlns="http://www.w3.org/1998/Math/MathML">
      <apply>
       <minus/>
       <apply>
        <times/>
        <apply>
```

```
    <plus/>
    <apply>
      <times/>
      <ci> kc30f1 </ci>
      <ci> pAKT308 </ci>
    </apply>
    <apply>
      <times/>
      <ci> AKTscale </ci>
      <ci> kc30f1 </ci>
      <ci> ppAKT308473 </ci>
    </apply>
  </apply>
  <ci> p2127 </ci>
  <apply>
    <plus/>
    <cn type="integer"> 1 </cn>
    <apply>
      <times/>
      <ci> alpha30 </ci>
      <apply>
        <plus/>
        <ci> Myc </ci>
        <ci> pMyc </ci>
      </apply>
    </apply>
  </apply>
</apply>
```

```
        &lt;apply&gt;
          &lt;times/&gt;
          &lt;ci&gt; Vmax30r1 &lt;/ci&gt;
          &lt;ci&gt; pp2127 &lt;/ci&gt;
        &lt;/apply&gt;
      &lt;/apply&gt;
    &lt;/math&gt;
  &lt;/kineticLaw&gt;
&lt;/reaction&gt;
&lt;reaction id="R31" name="R31" reversible="false"&gt;
  &lt;listOfProducts&gt;
    &lt;speciesReference species="E2F" stoichiometry="1"/&gt;
  &lt;/listOfProducts&gt;
  &lt;listOfModifiers&gt;
    &lt;modifierSpeciesReference species="Myc"/&gt;
    &lt;modifierSpeciesReference species="pMyc"/&gt;
  &lt;/listOfModifiers&gt;
  &lt;kineticLaw&gt;
    &lt;math xmlns="http://www.w3.org/1998/Math/MathML"&gt;
      &lt;apply&gt;
        &lt;plus/&gt;
        &lt;apply&gt;
          &lt;times/&gt;
          &lt;ci&gt; kc31f1 &lt;/ci&gt;
          &lt;apply&gt;
            &lt;plus/&gt;
            &lt;ci&gt; Myc &lt;/ci&gt;
            &lt;ci&gt; pMyc &lt;/ci&gt;
```

```
        </apply>
       </apply>
       <apply>
        <times/>
        <ci> kc31f2 </ci>
        <ci> E2F </ci>
       </apply>
      </apply>
     </math>
    </kineticLaw>
   </reaction>
   <reaction id="R32" name="R32" reversible="false">
    <listOfReactants>
     <speciesReference species="E2F" stoichiometry="1"/>
    </listOfReactants>
    <kineticLaw>
     <math xmlns="http://www.w3.org/1998/Math/MathML">
      <apply>
       <times/>
       <ci> kdeg32f1 </ci>
       <ci> E2F </ci>
      </apply>
     </math>
    </kineticLaw>
   </reaction>
   <reaction id="R33" name="R33" reversible="true">
    <listOfReactants>
     <speciesReference species="E2F" stoichiometry="1"/>
```

```
    &lt;speciesReference species="Rb" stoichiometry="1"/&gt;
  &lt;/listOfReactants&gt;
  &lt;listOfProducts&gt;
    &lt;speciesReference species="RbE2F" stoichiometry="1"/&gt;
  &lt;/listOfProducts&gt;
  &lt;kineticLaw&gt;
    &lt;math xmlns="http://www.w3.org/1998/Math/MathML"&gt;
      &lt;apply&gt;
        &lt;minus/&gt;
        &lt;apply&gt;
          &lt;times/&gt;
          &lt;ci&gt; ka33f1 &lt;/ci&gt;
          &lt;ci&gt; E2F &lt;/ci&gt;
          &lt;ci&gt; Rb &lt;/ci&gt;
        &lt;/apply&gt;
        &lt;apply&gt;
          &lt;times/&gt;
          &lt;ci&gt; kd33r1 &lt;/ci&gt;
          &lt;ci&gt; RbE2F &lt;/ci&gt;
        &lt;/apply&gt;
      &lt;/apply&gt;
    &lt;/math&gt;
  &lt;/kineticLaw&gt;
&lt;/reaction&gt;
&lt;reaction id="R34" name="R34" reversible="true"&gt;
  &lt;listOfReactants&gt;
    &lt;speciesReference species="Rb" stoichiometry="1"/&gt;
  &lt;/listOfReactants&gt;
```

```
  <listOfProducts>
   <speciesReference species="pRb" stoichiometry="1"/>
  </listOfProducts>
  <listOfModifiers>
   <modifierSpeciesReference species="CDK46cycD"/>
  </listOfModifiers>
  <kineticLaw>
   <math xmlns="http://www.w3.org/1998/Math/MathML">
    <apply>
     <minus/>
     <apply>
      <times/>
      <ci> kc34f1 </ci>
      <ci> CDK46cycD </ci>
      <ci> Rb </ci>
     </apply>
     <apply>
      <times/>
      <ci> Vmax34r1 </ci>
      <ci> pRb </ci>
     </apply>
    </apply>
   </math>
  </kineticLaw>
 </reaction>
 <reaction id="R35" name="R35" reversible="true">
  <listOfReactants>
   <speciesReference species="pRb" stoichiometry="1"/>
```

```
      </listOfReactants>
      <listOfProducts>
        <speciesReference species="pppRb" stoichiometry="1"/>
      </listOfProducts>
      <listOfModifiers>
        <modifierSpeciesReference species="CDK2cycE"/>
      </listOfModifiers>
      <kineticLaw>
        <math xmlns="http://www.w3.org/1998/Math/MathML">
          <apply>
            <minus/>
            <apply>
              <times/>
              <ci> kc35f1 </ci>
              <ci> CDK2cycE </ci>
              <ci> pRb </ci>
            </apply>
            <apply>
              <times/>
              <ci> Vmax35r1 </ci>
              <ci> pppRb </ci>
            </apply>
          </apply>
        </math>
      </kineticLaw>
    </reaction>
  </listOfReactions>
</model>
```

</sbml>
