# Supplementary material for: Systematic analysis of network-driven adaptive resistance to CDK4/6 and oestrogen receptor inhibition using meta-dynamic network modelling
Source: eLife. 2026 Aug 27;12:RP87710. doi: 10.7554/eLife.87710 (PMC13521565; doi:10.7554/eLife.87710)
Supplement: Supplementary file 3 [file elife-87710-mdarchecklist1.docx]

Materials Design Analysis Reporting (MDAR) Checklist

Materials:

|  | Indicate where provided: | N/A |
| --- | --- | --- |
| Newly created materials |  | N/A |
| Antibodies |  | N/A |
| DNA and RNA sequences |  | N/A |
| Cell materials |  | N/A |
| Experimental animals Indicate where |  | N/A |
| Plants and microbes |  | N/A |
| Human research participants |  | N/A |

Design:

|  | Indicate where provided: | N/A |
| --- | --- | --- |
| Study protocol |  | N/A |
| Laboratory protocol |  | N/A |
| Experimental study design (statistics details) |  | N/A |
| Sample definition and in-laboratory replication |  | N/A |
| Ethics |  | N/A |
| Dual Use Research of Concern (DURC) |  | N/A |

Analysis:

|  | Indicate where provided: | N/A |
| --- | --- | --- |
| Attrition |  | N/A |
| Statistics |  | N/A |
| Data availability |  | N/A |
| Code availability | https://github.com/IntegratedNetworkModellingLab/Meta_Dynamic_Analysis_eLife |  |

Reporting:

|  | Indicate where provided: | N/A |
| --- | --- | --- |
| Adherence to community standards |  | N/A |
