# Supplementary material for: Systematic analysis of network-driven adaptive resistance to CDK4/6 and oestrogen receptor inhibition using meta-dynamic network modelling
Source: eLife. 2026 Aug 27;12:RP87710. doi: 10.7554/eLife.87710 (PMC13521565; doi:10.7554/eLife.87710)
Supplement: Peer review history

# Editor report: eLife Assessment

**DOI:** 10.7554/eLife.87710.3.sa0

**Authors:** Mariana Gómez-Schiavon

**Affiliations:** Universidad Nacional Autónoma de México Mexico

**Keywords:** Incomplete, Useful

This manuscript presents a **useful** computational framework for systematically characterising how heterogeneity in initial conditions or biophysical parameters shapes the dynamic behaviour of protein signalling networks, with potential relevance to understanding adaptive drug resistance. While the approach represents a significant methodological contribution, the extent to which its conclusions are biologically informative remains debated, as the model is only qualitatively compared with experimental data and lacks quantitative validation. As a result, the strength of evidence supporting the mechanistic claims is viewed as **incomplete**.

# Referee report: Joint Public Review

**DOI:** 10.7554/eLife.87710.3.sa1

In this manuscript, the authors proposed an approach to systematically characterise how heterogeneity in a protein signalling network affects its emergent dynamics, with particular emphasis on drug-response signalling dynamics in cancer treatments. They named this approach Meta Dynamic Network (MDN) modelling, as it aims to consider the potential dynamic responses globally, varying both initial conditions (i.e., expression levels) and biophysical parameters (i.e., protein interaction parameters). By characterising the "meta" response of the network, the authors propose that the method can provide insights not only into the possible dynamic behaviours of the system of interest but also into the likelihood and frequency of observing these dynamic behaviours in the natural system.

The authors study the Early Cell Cycle (ECC) network as a proof of concept, focusing on pathways involving PI3K, EGFR, and CDK4/6 with the aim of identifying mechanisms that may underlie resistance to CDK4/6 inhibition in cancer. The biochemical reaction model comprises 50 state variables and 94 kinetic parameters, implemented in SBML and simulated in Matlab. A central component of the study is the generation of large ensembles of model instances, including 100,000 randomly sampled parameter sets intended to represent intra-tumour heterogeneity. On the basis of these simulations, the authors conclude that heterogeneity in kinetic rate parameters plays a stronger role in driving adaptive resistance than variation in baseline protein expression levels, and that resistance emerges as a network-level property rather than from individual components alone. The revised manuscript provides additional clarification regarding aspects of the simulation and filtering procedures and frames the comparison with experimental data as qualitative. Nonetheless, the study is best interpreted as a theoretical and exploratory analysis of the model's behaviour under heterogeneous conditions. Consequently, questions remain regarding the biological grounding of the sampled parameter regimes and the extent to which the reported frequencies of resistance-associated behaviours can be directly interpreted in physiological terms.

While the authors propose a potentially useful computational framework to explore how heterogeneity shapes dynamic responses to drug perturbation, a number of important conceptual and methodological concerns remain to be addressed:

(1) The sampling of kinetic parameters constitutes the backbone of the manuscript, yet important concerns remain regarding its biological grounding and transparency. Although the revised version provides additional clarification on the exploration of "model instances", it is still not sufficiently clear how parameter values and initial conditions are generated, nor how the chosen ranges relate to biological measurements. The kinetic rates are sampled over broad intervals without explicit justification in terms of experimentally measured bounds or inferred distributions. As a consequence, it remains uncertain whether the ensemble of simulated behaviours reflects physiologically plausible cellular regimes or primarily the properties of the assumed parameter space. In this context, the large-scale sampling (100,000 parameter sets) resembles a Monte Carlo exploration of the model rather than a biologically calibrated representation of tumour heterogeneity.

Furthermore, the adequacy of the sampling strategy in such a high-dimensional space (94 free parameters) remains open to question. In the absence of biologically informed constraints, the combinatorial space of possible parameter configurations is vast, and it is unclear to what extent the sampled ensembles can be considered representative. This issue is particularly relevant because the manuscript interprets the frequency of resistance-associated behaviours as indicative of their likelihood.

The validation presented in Figure 7 does not fully resolve these concerns. The comparison with experimental data is qualitative, and the simulations are performed in arbitrary time units, which complicates direct interpretation alongside time-resolved experimental measurements. Moreover, certain qualitative discrepancies between simulated and experimental trends (e.g., persistent versus decreasing CDK4/6 activity) are not thoroughly discussed. As this figure represents the primary empirical reference point in the manuscript, the extent to which the model captures experimentally observed dynamics remains uncertain.

Finally, aspects of presentation continue to limit transparency. Parameter ranges are described at different points in the manuscript but are not consolidated clearly in the Methods, and the definition of initial conditions remains ambiguous - particularly whether these correspond to conserved quantities or to the dynamic variables used to initialise simulations. In addition, the exact number of model instances underlying specific analyses and figures is not always explicit. Greater clarity on these issues is essential for assessing reproducibility and for interpreting the quantitative claims of the study.

(2) A central conclusion of the manuscript is that heterogeneity in protein-protein interaction kinetics is a stronger driver of adaptive resistance than heterogeneity in protein expression levels. To assess the latter, the authors fix a nominal set of kinetic parameters and generate 100,000 random initial concentrations for the 50 model species. However, according to the simulation protocol described in the manuscript, each trajectory includes three phases: (i) simulation under starvation conditions to equilibrium, (ii) mitogenic stimulation to a second ("fed") equilibrium, and (iii) application of drug treatment. The equilibrium concentrations reached in phases (i) and (ii) are determined by the kinetic parameters of the model and are independent of the initial concentrations, provided the system converges to a stable steady state. In dynamical systems terms, stable equilibria are defined by the parameter set and attract all initial conditions within their basin of attraction. Since the kinetic parameters are fixed in this experiment, the pre-treatment equilibrium that serves as the starting point for drug application should likewise be fixed. Under these conditions, it is therefore not unexpected that sampling a large number of initial concentrations has limited influence on the treated dynamics.

This raises conceptual questions about the interpretation of the comparison between kinetic and expression heterogeneity. If the system converges to a unique stable steady state prior to treatment, then variability in initial concentrations does not propagate into variability in drug response, and the observed dominance of kinetic heterogeneity may partly reflect this structural property of the model rather than a biological principle. Clarification is needed regarding whether multiple steady states exist under the nominal parameter set, and if so, how basins of attraction are explored.

More broadly, it remains unclear why initial protein concentrations can be sampled independently of the kinetic parameters. In biological systems, steady-state expression levels are typically determined by the underlying kinetic rates. A more consistent approach might require constraining initial concentrations to correspond to equilibrium states of the chosen parameter set, thereby introducing relationships between at least some of the 50 initial conditions and the 94 kinetic parameters. Finally, the manuscript employs a non-standard terminology regarding "initial conditions," which may further obscure interpretation of these results and would benefit from clarification.

(3) The technical implementation of the modelling and simulation framework remains difficult to evaluate due to insufficient methodological detail. Although the authors state that kinetic parameters are randomly sampled, the manuscript does not specify the distributions from which parameters are drawn, nor whether potential correlations between parameters are considered or explicitly ignored. Without this information, it is not possible to assess how implicit modelling assumptions shape the ensemble of simulated behaviours. Given that the conclusions rely on frequency-based interpretations across sampled parameter sets, greater transparency regarding the sampling procedure is essential.

A further concern relates to the parameter filtering step. The authors report that the "vast majority" of sampled parameter sets produced systems that were "too stiff," and that these were excluded on the grounds that stiff dynamics are not biologically plausible. However, the manuscript does not clearly define how stiffness is assessed, nor why stiffness is interpreted as biologically unrealistic rather than as a numerical property of the formulation. In standard practice, stiff systems are typically handled using appropriate implicit solvers rather than being discarded. Similarly, parameter sets that produce negative state values are excluded, yet such behaviour may arise from numerical artefacts rather than from intrinsic model inconsistency. The rationale for excluding these parameter sets, rather than adapting the numerical scheme, is not sufficiently justified.

The reported rejection rate - approximately 90% of sampled parameter sets - is substantial and raises questions regarding the interplay between model structure, parameter ranges, and numerical methods. As currently described, the filtering step appears to select parameter sets based primarily on computational tractability rather than on experimentally motivated biological criteria. The manuscript would be strengthened by clarifying whether the retained parameter sets are representative of biologically meaningful regimes, and by distinguishing clearly between exclusions based on biological plausibility and those arising from numerical considerations.

Finally, important aspects of the simulation protocol require clarification. The model is simulated under "fasted" and "fed" conditions until equilibrium is reached, yet the criterion used to determine convergence is not specified. It would be important to describe how equilibrium is assessed (e.g., based on the norm of the time derivatives). Additionally, it remains unclear whether the mitogenic stimulus applied in the "fed" phase is assumed to be constant over time and, if so, how this assumption relates to biological experimental conditions. Greater detail on these implementation choices is necessary to ensure interpretability and reproducibility.

(4) The manuscript states that the modelling conclusions are strongly supported by existing literature; however, the validation presented does not fully substantiate this claim. As noted above, the comparison with CDK2 and CDK4/6 experimental data remains qualitative, and the use of arbitrary simulation time units complicates interpretation of temporal agreement. The extent to which the model quantitatively or mechanistically recapitulates experimentally observed dynamics therefore remains uncertain.

The claim that the model reproduces known resistance mechanisms is also difficult to assess in light of Figure S10, where a large fraction of network nodes (~80%) appear implicated in resistance under some conditions. If most components of the network can, in at least some parameter regimes, be associated with resistance phenotypes, the resulting lack of selectivity weakens the strength of model-based validation. It becomes challenging to distinguish specific mechanistic insights from generic consequences of network connectivity.

In addition, the Supplementary Information notes that certain components of the mitogenic and cell-cycle pathways were abstracted or excluded in order to maintain computational tractability. While such abstraction is understandable in a large ODE framework, it raises interpretative questions. Proteins identified as potential resistance drivers within the model may, in some cases, represent aggregated or simplified pathway effects. Clarifying in the main text how such abstractions may influence the attribution of resistance mechanisms would strengthen the biological interpretation of the results.

Drug inhibition is central to the manuscript's conclusions. The revised version clarifies that inhibition is implemented as a fixed fractional modification of specific kinetic rate laws. This abstraction is appropriate for exploring network-level responses, but it represents a stylised perturbation rather than a pharmacologically calibrated model of drug action. For full interpretability and reproducibility, the mathematical form of the modified rate laws, as well as the timing of inhibition relative to network equilibration, should be specified unambiguously. The biological implications of the findings depend critically on understanding this modelling choice.

The one-at-a-time perturbation analysis presented in Figure 5 provides an interpretable ranking of first-order control points across the ensemble and offers mechanistic insight into primary sensitivities of the network. However, many targeted therapies act on multiple components, and resistance frequently arises through combinatorial mechanisms. The reported rankings should therefore be interpreted as identifying primary influences under isolated perturbations, rather than as a comprehensive account of multi-target drug behaviour.

Overall, the manuscript succeeds in presenting a conceptual and exploratory framework for analysing how signalling network topology can shape the qualitative landscape of adaptive responses under heterogeneous kinetic conditions. Its principal contribution lies in establishing a systematic platform for large-scale in silico exploration. At the same time, the current limitations in biological calibration, parameter grounding, and validation constrain the extent to which the conclusions can be interpreted as predictive or quantitatively representative of specific tumour contexts. Addressing these issues would further strengthen the connection between the theoretical landscape described here and experimentally observed resistance dynamics.

# Author comment: Author response

**DOI:** 10.7554/eLife.87710.3.sa2

**Authors:** Anthony Hart, Sungyoung Shin, Lan K Nguyen

**Affiliations:** Monash University Clayton Australia; Monash University Clayton Australia; Monash University Clayton Australia

The following is the authors’ response to the current reviews.

**eLife Assessment**

This manuscript presents a useful computational framework for systematically characterising how heterogeneity in initial conditions or biophysical parameters shapes the dynamic behaviour of protein signalling networks, with potential relevance to understanding adaptive drug resistance. While the approach represents a significant methodological contribution, the extent to which its conclusions are biologically informative remains debated, as the model is not qualitatively or quantitatively validated against experimental data. As a result, the strength of evidence supporting the mechanistic claims is viewed as incomplete.

We thank the editors and reviewers for their further assessment of the manuscript. The revised public review raises several issues that overlap with points addressed in our previous response, particularly around the intended scope of MDA modelling, the interpretation of parameter sampling, and the qualitative nature of the experimental comparison. In this final revision, we have made targeted clarifications in the main text, Methods, figure legends, and Supplementary Information to make these points more explicit for readers. We emphasise that the present work is intended as a theoretical and exploratory framework for mapping the qualitative dynamic behaviours accessible to a fixed network topology, rather than as a quantitatively calibrated model of a specific tumour or cell line.

**Joint Public Review:**

In this manuscript, the authors proposed an approach to systematically characterise how heterogeneity in a protein signalling network affects its emergent dynamics, with particular emphasis on drug-response signalling dynamics in cancer treatments. They named this approach Meta Dynamic Network (MDN) modelling, as it aims to consider the potential dynamic responses globally, varying both initial conditions (i.e., expression levels) and biophysical parameters (i.e., protein interaction parameters). By characterising the "meta" response of the network, the authors propose that the method can provide insights not only into the possible dynamic behaviours of the system of interest but also into the likelihood and frequency of observing these dynamic behaviours in the natural system.

The authors study the Early Cell Cycle (ECC) network as a proof of concept, focusing on pathways involving PI3K, EGFR, and CDK4/6 with the aim of identifying mechanisms that may underlie resistance to CDK4/6 inhibition in cancer. The biochemical reaction model comprises 50 state variables and 94 kinetic parameters, implemented in SBML and simulated in Matlab. A central component of the study is the generation of large ensembles of model instances, including 100,000 randomly sampled parameter sets intended to represent intra-tumour heterogeneity. On the basis of these simulations, the authors conclude that heterogeneity in kinetic rate parameters plays a stronger role in driving adaptive resistance than variation in baseline protein expression levels, and that resistance emerges as a network-level property rather than from individual components alone. The revised manuscript provides additional clarification regarding aspects of the simulation and filtering procedures and frames the comparison with experimental data as qualitative. Nonetheless, the study is best interpreted as a theoretical and exploratory analysis of the model's behaviour under heterogeneous conditions. Consequently, questions remain regarding the biological grounding of the sampled parameter regimes and the extent to which the reported frequencies of resistance-associated behaviours can be directly interpreted in physiological terms.

While the authors propose a potentially useful computational framework to explore how heterogeneity shapes dynamic responses to drug perturbation, a number of important conceptual and methodological concerns remain to be addressed:

(1) The sampling of kinetic parameters constitutes the backbone of the manuscript, yet important concerns remain regarding its biological grounding and transparency. Although the revised version provides additional clarification on the exploration of "model instances", it is still not sufficiently clear how parameter values and initial conditions are generated, nor how the chosen ranges relate to biological measurements. The kinetic rates are sampled over broad intervals without explicit justification in terms of experimentally measured bounds or inferred distributions. As a consequence, it remains uncertain whether the ensemble of simulated behaviours reflects physiologically plausible cellular regimes or primarily the properties of the assumed parameter space. In this context, the large-scale sampling (100,000 parameter sets) resembles a Monte Carlo exploration of the model rather than a biologically calibrated representation of tumour heterogeneity.

Parameters were sampled from a uniform distribution spanning values 10-5 to 104. Conserved totals were sampled from the range 100 to 104. Each of these is roughly in line with measured spans of orders of magnitude for parameter values and protein expression (REF). Again, we would like to point out that we intentionally kept our ranges broad, and sampled from uniform distributions, to assess upper bounds of heterogeneity, not biologically informed heterogeneity. We also comment on the likely effects of expanding these ranges in our response to (26) in our original rebuttal.

Main text has been updated to include this information. LINES: 175-179

Furthermore, the adequacy of the sampling strategy in such a high-dimensional space (94 free parameters) remains open to question. In the absence of biologically informed constraints, the combinatorial space of possible parameter configurations is vast, and it is unclear to what extent the sampled ensembles can be considered representative. This issue is particularly relevant because the manuscript interprets the frequency of resistance-associated behaviours as indicative of their likelihood.

This was addressed extensively in our original rebuttal, response to point (3). A new section was added to the supplementary text, along with new figures demonstrating the validity of the claims.

The validation presented in Figure 7 does not fully resolve these concerns. The comparison with experimental data is qualitative, and the simulations are performed in arbitrary time units, which complicates direct interpretation alongside time-resolved experimental measurements. Moreover, certain qualitative discrepancies between simulated and experimental trends (e.g., persistent versus decreasing CDK4/6 activity) are not thoroughly discussed. As this figure represents the primary empirical reference point in the manuscript, the extent to which the model captures experimentally observed dynamics remains uncertain.

This was addressed in the original rebuttal, response to point (12). The actual time units are arbitrary in the sense that they are determined by the units of the parameters in our model. It is important to understand that the meta-dynamic network analysis is not calibrated to data and so the meaning of time units is far less important than the distribution of behaviours. We have updated the figure to reflect the arbitrary units of time in our simulations.

Finally, aspects of presentation continue to limit transparency. Parameter ranges are described at different points in the manuscript but are not consolidated clearly in the Methods, and the definition of initial conditions remains ambiguous - particularly whether these correspond to conserved quantities or to the dynamic variables used to initialise simulations. In addition, the exact number of model instances underlying specific analyses and figures is not always explicit. Greater clarity on these issues is essential for assessing reproducibility and for interpreting the quantitative claims of the study.

(2) A central conclusion of the manuscript is that heterogeneity in protein-protein interaction kinetics is a stronger driver of adaptive resistance than heterogeneity in protein expression levels. To assess the latter, the authors fix a nominal set of kinetic parameters and generate 100,000 random initial concentrations for the 50 model species. However, according to the simulation protocol described in the manuscript, each trajectory includes three phases: (i) simulation under starvation conditions to equilibrium, (ii) mitogenic stimulation to a second ("fed") equilibrium, and (iii) application of drug treatment. The equilibrium concentrations reached in phases (i) and (ii) are determined by the kinetic parameters of the model and are independent of the initial concentrations, provided the system converges to a stable steady state. In dynamical systems terms, stable equilibria are defined by the parameter set and attract all initial conditions within their basin of attraction. Since the kinetic parameters are fixed in this experiment, the pre-treatment equilibrium that serves as the starting point for drug application should likewise be fixed. Under these conditions, it is therefore not unexpected that sampling a large number of initial concentrations has limited influence on the treated dynamics.

This raises conceptual questions about the interpretation of the comparison between kinetic and expression heterogeneity. If the system converges to a unique stable steady state prior to treatment, then variability in initial concentrations does not propagate into variability in drug response, and the observed dominance of kinetic heterogeneity may partly reflect this structural property of the model rather than a biological principle. Clarification is needed regarding whether multiple steady states exist under the nominal parameter set, and if so, how basins of attraction are explored.

More broadly, it remains unclear why initial protein concentrations can be sampled independently of the kinetic parameters. In biological systems, steady-state expression levels are typically determined by the underlying kinetic rates. A more consistent approach might require constraining initial concentrations to correspond to equilibrium states of the chosen parameter set, thereby introducing relationships between at least some of the 50 initial conditions and the 94 kinetic parameters. Finally, the manuscript employs a non-standard terminology regarding "initial conditions," which may further obscure interpretation of these results and would benefit from clarification.

This was addressed in the original rebuttal, response to point (4). Text was modified to clarify what was meant by initial conditions to clarify that this meant the conserved total for the protein species. A supplementary figure (supp. fig. 4) was added to demonstrate that changes to the conserved totals of protein species does, in fact, shift the dynamics and steady state equilibria of protein species. Text was updated throughout the paper to ensure that our definition of ‘initial conditions’ was consistent throughout the text.

(3) The technical implementation of the modelling and simulation framework remains difficult to evaluate due to insufficient methodological detail. Although the authors state that kinetic parameters are randomly sampled, the manuscript does not specify the distributions from which parameters are drawn, nor whether potential correlations between parameters are considered or explicitly ignored. Without this information, it is not possible to assess how implicit modelling assumptions shape the ensemble of simulated behaviours. Given that the conclusions rely on frequency-based interpretations across sampled parameter sets, greater transparency regarding the sampling procedure is essential.

Updated the main text to clarify random sampling from a log transformed uniform distribution. LINES: 175-179

A further concern relates to the parameter filtering step. The authors report that the "vast majority" of sampled parameter sets produced systems that were "too stiff," and that these were excluded on the grounds that stiff dynamics are not biologically plausible. However, the manuscript does not clearly define how stiffness is assessed, nor why stiffness is interpreted as biologically unrealistic rather than as a numerical property of the formulation. In standard practice, stiff systems are typically handled using appropriate implicit solvers rather than being discarded. Similarly, parameter sets that produce negative state values are excluded, yet such behaviour may arise from numerical artefacts rather than from intrinsic model inconsistency. The rationale for excluding these parameter sets, rather than adapting the numerical scheme, is not sufficiently justified.

The reported rejection rate - approximately 90% of sampled parameter sets - is substantial and raises questions regarding the interplay between model structure, parameter ranges, and numerical methods. As currently described, the filtering step appears to select parameter sets based primarily on computational tractability rather than on experimentally motivated biological criteria. The manuscript would be strengthened by clarifying whether the retained parameter sets are representative of biologically meaningful regimes, and by distinguishing clearly between exclusions based on biological plausibility and those arising from numerical considerations.

This was extensively addressed in the original rebuttal, response to points (6) and (7). Main text was updated to clarify that a solver specific for stiff systems was used. Furthermore, we addressed this issue but consequential analysis revealed that lack of drug response and not achieving steady state in the simulated time period now accounted for the majority of filtering. This had no effect on the distributions of behaviours identified in our analyses. Main text was updated to reflect these changes. Rejection rate was explicitly discussed in main text.

Finally, important aspects of the simulation protocol require clarification. The model is simulated under "fasted" and "fed" conditions until equilibrium is reached, yet the criterion used to determine convergence is not specified. It would be important to describe how equilibrium is assessed (e.g., based on the norm of the time derivatives). Additionally, it remains unclear whether the mitogenic stimulus applied in the "fed" phase is assumed to be constant over time and, if so, how this assumption relates to biological experimental conditions. Greater detail on these implementation choices is necessary to ensure interpretability and reproducibility.

This was addressed in the original rebuttal, response to point (8). Clarification about simulations were added to main text, including explicitly stating that mitogenic and drug inputs were continuous stepwise functions and how steady state equilibrium was defined/calculated.

(4) The manuscript states that the modelling conclusions are strongly supported by existing literature; however, the validation presented does not fully substantiate this claim. As noted above, the comparison with CDK2 and CDK4/6 experimental data remains qualitative, and the use of arbitrary simulation time units complicates interpretation of temporal agreement. The extent to which the model quantitatively or mechanistically recapitulates experimentally observed dynamics therefore remains uncertain.

This was addressed in the original rebuttal, response to points (13) and (14). Wording was changed to remove the suggestion of strong evidence and the tone was shifted to reflect reasonable qualitative support for our analysis, not strong evidence.

The claim that the model reproduces known resistance mechanisms is also difficult to assess in light of Figure S10, where a large fraction of network nodes (~80%) appear implicated in resistance under some conditions. If most components of the network can, in at least some parameter regimes, be associated with resistance phenotypes, the resulting lack of selectivity weakens the strength of model-based validation. It becomes challenging to distinguish specific mechanistic insights from generic consequences of network connectivity.

In addition, the Supplementary Information notes that certain components of the mitogenic and cell-cycle pathways were abstracted or excluded in order to maintain computational tractability. While such abstraction is understandable in a large ODE framework, it raises interpretative questions. Proteins identified as potential resistance drivers within the model may, in some cases, represent aggregated or simplified pathway effects. Clarifying in the main text how such abstractions may influence the attribution of resistance mechanisms would strengthen the biological interpretation of the results.

This was addressed in the original rebuttal, response to points (15). The discussion was significantly revised to reflect our reasoning with respect to our conclusions. We completely understand that more work could be done to verify our claims, however, our intention is to demonstrate the generalised relationship between network heterogeneity and drug resistance, not to predict patient-specific resistance mechanisms.

Drug inhibition is central to the manuscript's conclusions. The revised version clarifies that inhibition is implemented as a fixed fractional modification of specific kinetic rate laws. This abstraction is appropriate for exploring network-level responses, but it represents a stylised perturbation rather than a pharmacologically calibrated model of drug action. For full interpretability and reproducibility, the mathematical form of the modified rate laws, as well as the timing of inhibition relative to network equilibration, should be specified unambiguously. The biological implications of the findings depend critically on understanding this modelling choice.

All equations were included in the supplementary model files, including typeset ODEs, as requested by the reviewers. R15 and R27 contain the relevant equations, which specify the exact implementation of the drug inhibition. Number of time units per simulation phase now included in main text. LINES: 166 – 168

The one-at-a-time perturbation analysis presented in Figure 5 provides an interpretable ranking of first-order control points across the ensemble and offers mechanistic insight into primary sensitivities of the network. However, many targeted therapies act on multiple components, and resistance frequently arises through combinatorial mechanisms. The reported rankings should therefore be interpreted as identifying primary influences under isolated perturbations, rather than as a comprehensive account of multi-target drug behaviour.

Overall, the manuscript succeeds in presenting a conceptual and exploratory framework for analysing how signalling network topology can shape the qualitative landscape of adaptive responses under heterogeneous kinetic conditions. Its principal contribution lies in establishing a systematic platform for large-scale in silico exploration. At the same time, the current limitations in biological calibration, parameter grounding, and validation constrain the extent to which the conclusions can be interpreted as predictive or quantitatively representative of specific tumour contexts. Addressing these issues would further strengthen the connection between the theoretical landscape described here and experimentally observed resistance dynamics.

**Joint Recommendations for the authors:**

(1) Supplementary Figure S4 is not sufficiently explained in its current form. The structure of the figure, the meaning of its colour coding, and the intended interpretation are not clearly described, making it difficult for readers to extract the key message without substantial inference. Given that the manuscript relies heavily on large-scale ensemble analyses, clear visual communication is essential. A more detailed legend, explicit definition of axes and colour scales, and improved visual labelling would substantially enhance clarity, accessibility, and reproducibility.

Supp. Fig. 4 legend updated with additional detail. LINES: Supp. Text. 256 - 263

(2) The approximately 90% rejection rate of sampled parameter sets should be reported explicitly in the main text of the manuscript rather than only in the Supplementary Information. Given the central role of large-scale parameter sampling in the study, this level of exclusion is a critical aspect of the modelling workflow and directly affects the interpretation of robustness and representativeness. Clear disclosure in the main text would allow readers to properly evaluate the effective size of the analysed ensemble and the implications of the filtering procedure for the generality of the conclusions.

This was explicitly addressed in the original rebuttal.

(3) The model would benefit from quantitative validation against experimental data. In Figure 7C, the authors note in the response letter that the simulations are performed in arbitrary time units. However, the figure itself labels the time axis in hours, which may lead readers to infer a direct quantitative correspondence between simulated and experimental time courses. If the simulations are not calibrated to real time, this labelling is potentially misleading and should be corrected. Either the model should be explicitly time-calibrated and quantitatively compared to experimental measurements, or the figure should clearly indicate that the time axis is dimensionless. Clarifying this point is essential to avoid overinterpretation of the agreement between model and data.

Label updated.

The following is the authors’ response to the original reviews.

**Joint Public Reviews:**

In this manuscript, the authors proposed an approach to systematically characterise how heterogeneity in a protein signalling network affects its emergent dynamics, with particular emphasis on drug-response signalling dynamics in cancer treatments. They named this approach Meta Dynamic Network (MDN) modelling, as it aims to consider the potential dynamic responses globally, varying both initial conditions (i.e., expression levels) and biophysical parameters (i.e., protein interaction parameters). By characterising the "meta" response of the network, the authors propose that the method can provide insights not only into the possible dynamic behaviours of the system of interest but also into the likelihood and frequency of observing these dynamic behaviours in the natural system.

The authors studied the Early Cell Cycle (ECC) network as a proof of concept, specifically focusing on PI3K, EGFR, and CDK4/6, with particular interest in identifying the mechanisms that cancer could potentially exploit to display drug resistance. The biochemical reaction model consists of 50 equations (state variables) with 94 kinetic parameters, described using SBML and computed in Matlab. Based on the simulations, the authors concluded the following main points: a large number of network states can facilitate resistance, the individual biophysical parameters alone are insufficient to predict resistance, and adaptive resistance is an emergent property of the network. Finally, the authors attempt to validate the model's prediction that differential core sub-networks can drive drug resistance by comparing their observations with the knock-out information available in the literature. The authors identified subnetworks potentially responsible for drug resistance through the inhibition of individual pathways. Importantly, some concerns regarding the methodology are discussed below, putting in doubt the validity of the main claims of this work.

While the authors proposed a potentially useful computational approach to better understand the effect of heterogeneity in a system's dynamic response to a drug treatment (i.e., a perturbation), there are important weaknesses in the manuscript in its current form:

(1) It is unclear how the random parameter sets (i.e., model instances) and initial conditions are generated, and how this choice biases or limits the general conclusions for the case studied. Particularly, it is not evident how the kinetic rates are related to any biological data, nor if the parameter distributions used in this study have any biological relevance.

(2) Related to this problem, it is not clear whether the considered 100,000 random parameter samples sufficiently explore parameter space due to the combinatorial explosion that arises from having 94 free parameters, nor 100,000 random initial conditions for a system with 50 species (variables).

(3) Moreover, the authors filter out all the cases with stiff behaviour. This filtering step appears to select model parameters based on computational convenience, rather than biological plausibility.

(4) Also, it is not clear how exactly the drug effect is incorporated into the model (e.g., molecular inhibition?), nor how it is evaluated in the dynamic simulations (e.g., at the beginning of the simulation?). Moreover, in a complex network, the results may differ depending on whether the inhibition is applied from the start or after the network has reached a stable state.

(5) On the same line, the conclusions need to be discussed in the context of stability, particularly when evaluating the role of initial conditions. As stable steady states are determined by the model parameters, once again, the details of how the perturbation effect is evaluated on the simulation dynamics are critical to interpret the results.

(6) The presented validation of the model results (Fig. 7) is only qualitative, and the interpretation is not carefully discussed in the manuscript, particularly considering the comparison between fold-change responses without specifying the baseline states.

We thank the reviewers for their thoughtful and constructive comments. In response to their comments, we have undertaken a substantial revision to address all the comments, improve clarity, transparency, and robustness while preserving the paper’s core contribution: a principled, scalable framework (MDN) for mapping how molecular heterogeneity and network architecture shape adaptive drug-response dynamics. At a high level, we clarified the study design and analysis goals, tightened definitions, and added methodological detail where it most advances interpretability. Importantly, these updates leave the analytical pipelines and major conclusions unchanged.

Conceptually, we now make explicit that our objective is coverage of the output space of qualitative dynamics supported by the network topology, not exhaustive enumeration of parameter space. To support this, we added a convergence analysis and clarified that “triplicates” refers to independent ensembles used to demonstrate reproducibility. We also refined how we describe and implement initial conditions (as conserved total abundances that encode expression heterogeneity) and reframed filtering as minimal numerical/feasibility checks, using rejection sampling to obtain the prespecified ensemble size. Solver choices and input modelling (constant step mitogen/drug) are now spelled out succinctly.

We expanded the model specification and rationale (complete reaction list with rate laws and brief biological justifications in the Supplement) and unified terminology throughout. Figures and legends have been overhauled for readability and accuracy, with missing labels added and ordering corrected. For validation, we clarified the nature of the single-cell reporter readout, improved Figure 7’s presentation, and emphasised - consistent with our aims - that comparisons are qualitative.

Finally, we have rewritten the Discussion to centre on interpretation, implications, and connect our findings to the literature. It now: (i) frames MDN as a systems-level framework that links molecular heterogeneity to qualitative signalling “meta-dynamics” and adaptive escape under constant drug pressure; (ii) highlights two key findings: an asymmetry in control (interaction kinetics exert stronger, more consistent influence than protein abundance) and a topology-driven convergence whereby a vast parameter space funnels into a finite set of recurrent behaviours; (iii) shows that resistance is a network-level property, with many possible routes but a small set of recurrent hubs/modules dominating; and (iv) provides a qualitative alignment with single-cell reporter data while clarifying the intent and limits of that comparison. Moreover, we now explicitly discuss limitations (rate-law simplifications, broad priors, determinism, and modular abstractions) and outline next steps for future research, including data-constrained priors and stochastic extensions.

We believe these revisions materially strengthen the manuscript and fully address all the reviewers’ comments. A detailed, point-by-point response follows.

**Joint Recommendations for the Authors:**

(1) It is confusing exactly what are the different sets evaluated in each cases, e.g. "generated 100,000 model instances, each with the same set of ICs but a unique set of randomly generated parameter values" (lines 299-300), "generated 100,000 model instances (in triplicate), each with the same set of 'nominal' parameter values (see supplementary Table S1), and a unique set of ICs, and repeated the analysis as performed previously" (lines 366-368), "combined the 1000 IC sets with each parameter set to create 1000 model instances" (lines 382-383), "repeated for 1000 parameter sets, allowing us to observe how frequently IC variation induced adaptive resistance independent of the chosen parameter set" (lines 386-387). A small table or just a clearer explanation is needed.

In response to these comments, we have revised the main text to clarify the process of model instance generation. Specifically, we have made changes at page 7: line 297 - page 8: line 302, page 8: lines 305 - 310, page 9: lines 372-378, and page 9: line 384 – page 10: line 399 in the revised main text.

We have also added a new Figure (Figure S1) to the supplementary file to allow readers to visualise the model generation process for each relevant set of experiments. Supplementary figures are referenced in the main text where appropriate.

(2) The authors mentioned performing each simulation in triplicate, which is puzzling as the model is based on deterministic ODEs with fixed parameters for each simulation. Under such conditions, one would anticipate identical results from multiple simulations with the same initial conditions and fixed parameters. Perhaps the authors expect the model to exhibit chaos or aim to assess the precision of the parameter estimates through triplicate simulations. Further clarification from the authors would be valuable to comprehend the rationale behind conducting triplicate simulations in a deterministic setting.

We agree that repeating deterministic ODE simulations with identical inputs would be redundant. In our study, “triplicate” referred instead to generating three independent ensembles of 100,000 unique model instances each, where model parameters (or initial conditions) were randomly resampled. These ensembles were analysed separately to assess whether the inferred meta-dynamic distributions converged robustly. Indeed, the distributions from the three replicates were nearly indistinguishable, confirming that the results are reproducible and not artefacts of a particular random draw.

We have revised the main text to clarify this distinction (page 8: lines 305 - 310) and added an extended explanation for meta-dynamic behaviour convergence in the new section Error Convergence in the supplementary text (page 6: lines 184 - 210).

(3) While the lack of a connection between model parameters and biological data (mentioned in the public review) may not be a fatal flaw in the manuscript, the concern about the 100,000 random samples being insufficient to explore the parameter space is valid. In a thought experiment, considering the high and low rate for each parameter and the combinatorial explosion of possibilities (2^94), the number of simulations performed (100,000) represents only an extremely small fraction of the entire parameter space (~1/10^(23)). This limitation might not accurately capture the true heterogeneity present inside a solid tumour. One potential solution is to determine biological bounds on model parameters through data fitting, which can provide more meaningful constraints for the simulations. Alternatively, increasing the number of simulations and adopting more efficient sampling techniques can enhance the coverage of possible parameter sets.

We thank the reviewer for this insightful comment. We agree that the 94-dimensional parameter space is vast, and that 100,000 simulations represent only a fraction of the total combinatorial possibilities. However, the objective of our study is not to exhaustively sample the entire parameter space, but rather to sufficiently sample the ‘output space’ - that is, the complete spectrum of qualitative dynamic behaviours the network topology can generate. The key question is whether 100,000 model instances are sufficient for the distribution of these output dynamics to converge.

To formally address this, we have performed a convergence analysis, which is now detailed in the new supplementary section "Error Convergence" (Supplementary text page 6: lines 184 - 210) and illustrated in Supplementary Figure S12. This analysis demonstrates that the mean squared error (MSE) between dynamic distributions from N and 2N simulations exponentially decreases as N increases, and the distribution of protein dynamics changes negligibly well before reaching 100,000 instances. Furthermore, performing the entire analysis in triplicate with independent random seeds yielded nearly identical meta-dynamic maps (average standard deviation < 0.04%), giving us high confidence that we have robustly captured the network's behavioural repertoire.

We believe this convergence occurs because the system is degenerate: many distinct parameter sets within the high-dimensional space map to the same qualitative outcome (e.g., 'rebound' or 'decreasing'). Our goal was to capture the set of possible outcomes, not every unique parameter combination that leads to them.

Regarding the parameter range, we intentionally chose a broad, unbiased range (10^-5^ to 10)as a proof-of-concept to delineate the theoretical upper limit of heterogeneity the network can support, thereby capturing even rare but potentially critical resistance dynamics. We agree with the reviewer that a future direction is to constrain these ranges using biological data. Such an approach would transition from defining what is possible (the focus of this manuscript) to predicting what is probable in a specific biological context. We have added this important point to the Discussion (page 16: lines 663-679) to highlight this avenue for future work.

(4) One of the manuscript's main results indicates that protein interactions play a more significant role in driving adaptive resistance than protein expression. To explore the impact of protein expression, the authors fixed a nominal parameter set and generated 100,000 initial concentrations of the 50 proteins in the ODE model. However, the simulations' equilibrium concentrations in the "starvation" and "fed" phases, which form the initial condition for the treated phase, are uniquely determined by the nominal model's kinetic parameters and not the initial conditions, which remain identical for each simulation. From a dynamical systems perspective, stable steady states are determined by the model parameters and attract all initial conditions within their basin of attraction. As a result, a random sampling of the initial conditions has a limited impact on the model dynamics. The authors' conclusion that "the ability of expression to induce resistance also seems to be dependent on the master parameter set" can be explained by this dynamical systems perspective, where the resistance state corresponds to a stable steady state determined by the master parameter set. Considering this, the evidence presented in the manuscript may not fully support the authors' conclusion regarding the importance of protein expressions relative to protein dynamics. The discrepancy might be attributed to a possible misunderstanding of this point, and further clarification from the authors could be helpful.

We thank the reviewer for the thoughtful perspective. We agree that, in a monostable system with fixed kinetic parameters and fixed conserved totals, varying only the initial split among moieties (e.g., X vs pX) will not change the final steady state; trajectories converge to the same attractor. In our analysis, however, “initial conditions” predominantly refer to total protein abundances (e.g., X_tot = X + pX + complexes), used as a proxy for expression heterogeneity. These totals are invariants on the simulated timescale (no synthesis/degradation in the pre-equilibration phases), and therefore alter the value of the steady state under a given parameter set. In other words, our IC sampling mostly varies conserved totals rather than merely redistributing a fixed total; hence the equilibrium reached after the starvation/fed pre-equilibrations depends on the sampled totals and the kinetics. This can be seen in the new Supplementary Figure S4, showing that changing the ICs does shift the eventual steady state even when kinetic parameters are fixed.

We have revised the text to: (1) define ICs explicitly as total abundances for multi-state species, (2) distinguish “initial split” from “conserved totals,” and (3) clarify that expression effects are context-dependent rather than universally dominant (page 4: lines 139-141 and page 10: lines 413-416)

(5) Additionally, it is important to note that the random sampling of 100,000 initial concentrations might not sufficiently explore the vast space of possible initial conditions. In the thought experiment mentioned earlier, where each protein can have high or low expression concentrations, there are approximately 2^(50) = ~10^(15) possible combinations of initial concentrations. Thus, the 100,000 random simulations only represent around ~1/10^(10) of the possible initial conditions in this simplistic scenario. Consequently, this limited sampling of initial conditions may not provide enough information to draw meaningful conclusions, even if the initial conditions were more directly linked to kinetic rates.

Please see our response to Comment (3). Briefly, our ICs are continuous total abundances (conserved moieties), not binary high/low states; many IC configurations converge to the same qualitative attractors, so we estimate distributional properties rather than enumerate all combinations. Our convergence diagnostics (independent replicates and sample-size doubling) show that the meta-dynamic distributions stabilise well before N=100,000 (see Supplementary Figure S12). We have clarified this in the Supplementary Information (Error Convergence section) with the new convergence results.

(6) The authors implement a parameter selection step in the manuscript, where they filter out parameter sets that lead to what they term non-biological simulations. However, the rationale for determining if a given parameter set results in a stiff system of ODEs remains unclear. The authors cite references [38,39] to support the claim that stiff equations are not biologically plausible. Still, upon review, it is evident that [38] does not include the term "stiff," and [39] discusses using implicit methods to simulate stiff ODE models without specifically commenting on the biological plausibility of stiff systems. The manuscript lacks direct evidence to justify the conclusion that filtering out parameter sets that result in stiff ODE systems is reasonable. Since the filtering step accounts for the majority of discarded parameter sets, a stronger foundation is required to support the statement that stiff equations are non-biological.

We thank the reviewer for pointing out the issue in our original justification. The reviewer is correct: stiff systems are a common feature of biological models, and our claim that they are likely ‘biologically implausible’ was not well substantiated. The filtering of these model instances was, in fact, due to a computational limitation rather than a biological principle. The issue was that these parameter sets produced systems of ODEs that were so numerically stiff they were unsolvable within a reasonable timeframe by the SUNDIALS ODE solver suite, which is specifically designed for such systems.

Following the reviewer's comment, we investigated the source of this prohibitive stiffness. We discovered it was not an intrinsic property of the parameter sets themselves, but rather an artifact of our simulation setup. The extreme stiffness occurred almost exclusively during the initial integration timesteps, caused by the large initial discrepancy between the concentrations of active and inactive protein forms. This large discrepancy created the conditions for overtly stiff solutions i.e. unsolvable with implemented ODE solve settings. To overcome this problem, we set a large maximum number of steps in the ODE solver for the first couple of time points, enabling the solver to overcome the excessively stiff portion of the solve. We found that the vast majority of the previously 'unsolvable' model instances could now be successfully simulated. Consequently, the number of parameter sets discarded due to solver failure is now negligible (< 1%), and this filtering step no longer accounts for the majority of discarded parameter sets. Most importantly, the distributions of dynamics were not significantly altered by this adaptation.

We have revised the " Sampling and filtering of model instances (page 5: lines 174 – 189)" part in the Methods section to reflect this more accurate understanding. We have corrected our original claim regarding the biological plausibility of stiff systems and corrected our use of the references. Ref [38] was included to demonstrate that models of biological systems are stiff, which was a major conclusion of that paper, and [39] was originally included to demonstrate that solving ODEs is reliant on solvers that can integrate stiff systems. Upon review, ref [39] has been removed.

Overall, this investigation has made our analysis more robust by allowing us to include a wider, more representative range of parameter sets, and has tangibly improved the quality of our study.

(7) Additionally, it is important to consider the standard method for accounting for stiff systems, as presented in [39], which involves using implicit numerical methods for ODE simulation. The authors mention using numerical methods from the SUNDIALS suite, which includes implicit methods, but the specific numerical method used remains unclear. Furthermore, it would be valuable for the authors to disclose the number of parameter sets that were filtered to obtain the final set of 100,000 accepted parameter sets. This information would provide insights into the extent of filtering and the proportion of parameter sets that were excluded during the selection process.

We apologise for the lack of specific detail and have now updated the text. To clarify, all ODE simulations were performed using the CVODE solver from the SUNDIALS suite. This solver employs an implicit, variable-order, variable-step Backward Differentiation Formula (BDF) method, which is robust and specifically designed for handling the stiff systems common in biological network modelling. We have now explicitly stated this in the "ODE model construction, modelling, and simulations (page 4: lines 162 – 164)" section of the Methods.

Regarding the filtered parameters, we have included a revised and detailed discussion of this in the "Sampling and filtering of model instances (page 5: lines 174 – 189)" part in the Methods section (see our response to comment (6) above). Briefly, after applying the filters, ~40–45% of instances did not reach steady state within the simulation timeframe, and ~50–55% did not meet the minimum drug-response criterion. Approximately 10% satisfied all criteria and were retained for analysis. Importantly, we employed ‘rejection sampling’ and continued drawing until we had N = 100,000 accepted instances that satisfied all the criteria.

(8) An important step in the simulation process described by the authors is the simulation of the "fasted" and "fed" states until an equilibrium is reached. However, it is not clear how the authors determine if the system has reached an equilibrium. It would be helpful if the authors could provide more information regarding the criteria used to assess equilibrium in the simulations. Regarding the "fed" state, it is not explicitly stated whether the mitogen stimulus is assumed to be constant throughout the "fed" experiment. Considering the dynamic nature of mitogen stimulation in biological systems, it would be beneficial if the authors could clarify this assumption and discuss its biological relevance.

We apologise for the lack not specifying this in the original text. A simulation was considered to have reached equilibrium when the concentration of every protein species changed by < 1% over the final 100 time steps of the simulation phase. We have now added this criterion to the "Sampling and filtering of model instances (page 5: lines 177 – 179)" part of the Methods section.

Regarding the second part of the comment, in our simulations, both the mitogenic and the drug inputs were modelled as constant, stepwise functions that, once turned on, remained at a fixed concentration for the remainder of the simulation. The biological rationale for this choice was to rigorously test for bona fide adaptive resistance. By maintaining a constant mitogenic and drug pressure, we can ensure that any observed recovery in the activity of downstream proteins is due to the internal rewiring and adaptation of the signalling network itself, rather than an artefact of the removal or decay of the external stimulus/drugs. We have now clarified this rationale in the "ODE model construction, modelling, and simulations (page 4: lines 168 – 171)" part of the Methods section.

(9) The "Description of Model Scope and Construction" section in the Supplementary Information should include explicitly the model reactions and some discussion about their specific form (e.g., why is '(((kc2f1*pIR*PI3K) / (1 + (pS6K/Ki2))) + (kc2f2*pFGFR*PI3K))' representing the phosphorylation rate of PI3K, with pS6K in the denominator?).

The reviewer is right to ask for model justification. We have expanded the Supplementary “Description of Model Scope and Construction” section (page 2: line 63 – page 5: line 185) to include a complete reaction list with rate laws and a brief rationale for each. We also explain the specific PI3K phosphorylation term: activation by pIR and pFGFR is attenuated by pS6K via a denominator, which captures the well-described S6K-mediated negative feedback that reduces activation (e.g., via IRS1 phosphorylation).

(10) In line 349, the statement "Given that CDK46cycD is only strongly suppressed in just under 60% of the model instances (Figure 3C)" lacks clarity regarding where to look to interpret the 60% value. If this means that 4 out of the 7 model instances are resistant, and the other 2 proteins also have the same percentage of resistance, then there is no apparent reason to focus solely on CDK46cycD.

The reviewer is correct; the figure reference was an error, which has been rectified in the main text (page 9: line 355). The actual figure reference was to Supplementary Figure 2A, which shows the heatmap of all the frequencies for each protein dynamics for all the active protein forms. CDK4/6cycD shows a sustained decreasing dynamic for 59.93% of model instances, which is where this number was derived. We have also now explicitly referenced this number in the supplementary Figure 2A legend.

We focus on CDK4/6cycD because it is the direct pharmacological target of CDK4/6 inhibitors. Our point was to suggest that even when the target is suppressed in the majority of instances (~60%), this does not reliably propagate to uniform downstream inhibition across the network, thus highlighting emergent, network-driven adaptive responses.

(11) We observed that in Fig. 5A, the authors show that multiple pathways are blocked. However, it is unclear whether they reduced the value of one parameter in the experiment or simulated multiple combinations of parameter inhibition. Considering the large number of parameters (94) in the model, if the authors simulated all possible combinations of parameter inhibition, the number of combinations would be significantly more than 94. An actual inhibitor typically has an inhibitory effect on multiple molecules. Therefore, it would be necessary to identify the parameters that lead to drug resistance when multiple molecules are inhibited. However, examining the inhibition patterns for all 94 parameters would be practically impossible. As a potential approach, we suggest using ensemble learning techniques, such as random forests, to handle this problem efficiently. With a dataset of binary outputs indicating the presence or absence of resistance for a sufficient number of inhibition patterns, ensemble learning can be applied to find the parameters that contribute to drug resistance. Popular feature selection algorithms like Boruta could be utilised to identify the most relevant parameters. The results obtained by ensemble learning are similar to the ranking in Fig. 5C, potentially providing a more robust validation of the authors' findings. By incorporating these additional analyses, the authors could strengthen the reliability and significance of their results related to parameter inhibition and drug resistance.

We appreciate the suggestion and the opportunity to clarify. Figure 5A depicts multiple pathways were interrogated, but in the analysis, parameters were inhibited one at a time (OAT) - not in combination. We have revised the figure legend and added a section named “Protein knockdown perturbation analyses (page 6: lines 228 – 233)” in the Methods section to make this explicit. Moreover, some additional text in the main text has been slightly modified to make this clearer (page 11: lines 462-463, page 24: lines 856-857).

We chose the OAT design intentionally to obtain causal, first-order attribution of control points across a broad parameter ensemble without confounding from simultaneous co-inhibition. This provides an interpretable ranking of primary drivers (Figure 5C) that is consistent with the paper’s mechanistic focus. We agree that a multi-target inhibition approach could be a useful next step; however, an exhaustive combinatorial screen is beyond the scope of this proof-of-concept. In such future studies, the ensemble learning, as suggested by the reviewer, could be layered onto our MDN framework to assess robustness of the ranking under co-inhibition.

(12) In explaining the parameterization of the model, we find an implication of a quantitative model. However, upon examining the results in Fig. 7D, we observe that they are only qualitatively correct. When comparing Figs. 7A and 7C, we note that many model instances are immediately suppressed, and the time scale remains unknown. We believe it would be essential for the authors to explain how the model of this study maintains its quantitative nature despite the results in Fig. 7. If such an explanation cannot be provided, it raises concerns regarding the biological reliability of several findings within this study.

While our framework is built on quantitative ODEs, the validation we present in Figure 7 is indeed qualitative. This is an intentional and key feature of our study's design. Our goal was not to build a calibrated, quantitative model of a specific cell line (e.g., MCF10A), but rather to establish a proof-of-concept theoretical framework that systematically explores the full spectrum of dynamic behaviours a given network topology can possibly generate. To achieve this, we intentionally sampled parameters from a very broad, unbiased range to delineate the theoretical upper limit of heterogeneity. This *in silico* population is therefore designed to be far more heterogeneous than any single isogenic cell line.

The striking qualitative agreement seen between our meta-dynamic distributions and the single-cell data in Figure 7D is thus not a failure of quantitative prediction, but rather a strong validation of our core premise: that a significant degree of signalling heterogeneity exists in cell populations and that our framework can effectively capture its emergent properties.

Regarding the specific comment on Figure 7C, we apologise for the lack of clarity. Nominally, we chose to simulate for 24 hours however, the x-axis in our simulations represents arbitrary time units, as the timescale is dependent on the meaning/units of the parameter values. The goal is to compare the qualitative shape of the response (e.g., rebound, sustained decrease), not the absolute time in hours. Moreover the rapid initial suppression seen in many of our model instances (Fig 7C) is a direct parallel to the rapid suppression seen in the experimental data (Fig 7A). This initial phase is followed by a wide variety of adaptive behaviours (or lack thereof) in both our simulations and the real cells, which is the key phenomenon we are studying.

We have revised the text (page 14: lines 598-601) and Figure 7’s legend to state more explicitly that our validation is qualitative and to clarify the purpose of our broad, uncalibrated approach. We have also added a note in the Discussion (page 18: lines 744-747) that calibrating this framework with cell-line-specific data is a natural next step for generating quantitative, context-specific predictions.

(13) Related to the previous point, the experimental data is presented as fold-change during CDK4/6 inhibition, and we notice that the initial fold-change at time 0 varies between 1 and 1.8. The difference in initial fold-change is unclear to us, as our understanding of fold-change typically corresponds to the change from baseline, typically represented by the protein concentration at time 0.

Furthermore, while the experimental data exhibits uniformly decreasing CDK4/6 activity, a substantial number of simulations indicate constant CDK4/6cycD, showing a significant qualitative discrepancy between the simulations and experimental findings. This disparity makes it difficult for us to interpret the comparison between the two datasets effectively, given the complexities in comprehending the experimental fold-change figure.

As Figure 7 serves as the primary validation of model simulations in the manuscript, we believe that the current presentation may not provide a compelling reason to believe that the model accurately captures experimental data. To enhance clarity and validation, we suggest overlaying the experimental data over the simulations or considering the median and 10/90% percentile of the experimental data, which may potentially offer improved readability and facilitate a more robust interpretation of the comparison.

The experimental data from Yang et al. (ref 55, main text) measures kinase activity using a nucleus-to-cytoplasm translocation reporter system, wherein a bait protein is phosphorylated by the target kinase causing it to translocate from the nucleus to the cytoplasm. Hence, the y-axis represents the ratio of nuclear vs. cytoplasmic fluorescence, not a fold-change from a t=0 baseline. The variation in the starting value (between 1 and 1.8) reflects the inherent heterogeneity in the reporter's localization across individual cells even before the drug is added. We have updated the y-axis label and revised Fig. 7’s legend to state this explicitly.

The most likely explanation for the discrepancy between experimental dynamics and our simulation dynamics is that the experimental data comes from an isogenic cell line that is largely sensitive to CDK4/6 inhibition. Our simulations are derived from a very wide parameter sweep, where the intent is to represent all possible cell states. It is quite striking that that there is such a high correlation between the experimental data and simulations, indicating that perhaps the heterogeneity of even isogenic cell lines is significantly greater than might be intuited; a point we now mention in the revised Discussion (page 17: lines 716-727).

It is worth noting again, that our analysis is intentionally constructed to be as heterogeneous as possible, and is not trained on any biological data that might otherwise constrain the output-behaviour space. The isogenic cell line almost certainly represents a much more constrained output-behaviour space than our analysis.

The y-axis label has also been updated accordingly. As mentioned in (12) this result is intended as a qualitative validation, showing that cell lines indeed have highly variable signalling dynamics. Given the range of parameters tested, we think it is surprising that the degree of agreement between the experiment and our analysis is as high as it is. Again, we believe this suggests that heterogeneity may be more prevalent than is intuited. We do not believe we have made any strong quantitative claims in the main text, and we certainly aim to work towards biological, quantitative validation in the future. Finally, we altered the wording of the results heading (page 14: line 562) to make it clear that we are only making qualitative claims and removed the claim that the evidence was strong.

With these clarifications and corrections, we believe the validation is now much more compelling. The key point is not a perfect quantitative match, but the strong similarity in the distribution of heterogeneous behaviours.

(14) The authors mention simulating treatment with 10nM of CDK4/6i or Ei, but specific details on how this treatment is included in the model simulations are not provided. This lack of information makes it challenging to fully evaluate the comparison between model simulations and experimental evidence in Figure 7. It would be highly appreciated if the authors could clarify how the treatment with CDK4/6i or Ei is incorporated into the simulations to facilitate a better understanding and interpretation of the results.

To clarify, the effects of the inhibitors were incorporated directly into the kinetic rate laws of their respective target reactions.

CDK4/6 inhibitor (CDK4/6i): This was modelled as an inhibitor of the formation of the active CDK4/6-cyclin D complex. We have now explicitly detailed this in the description for reaction R27 in the "Description of Model Scope and Construction" section of the Supplementary Information.

Estrogen Receptor inhibitor (Ei): This was modelled as an inhibitor of the estrogen-dependent activation of the Estrogen Receptor. This is now explicitly detailed in the description for reaction R15 in the same supplementary section.

It is however important to reiterate that our goal in Figure 7 is qualitative, shape-based comparison; therefore, we used a fixed fractional inhibition (reported in Methods) rather than a calibrated IC50/Hill model.

(15) The authors state strong support for their modelling conclusions based on the literature. However, we still have concerns regarding the validation of the model against CDK2 or CDK4/6 data in Figure 7, as it appears less convincing to us. Furthermore, the authors list known resistance mechanisms that are replicated in their modelling. Nevertheless, we find the conclusion somewhat weakened by Figure S10, where approximately 80% of the nodes are implicated in some form of resistance pathway. This raises questions about the model's selectivity, as many proteins included in the model seem to drive resistance in some manner. In the Supplementary Information, the authors mention excluding or abstracting some protein species from the mitogenic and cell cycle pathways to manage computational resources effectively. This abstraction makes it difficult to determine if the proteins identified as potential drivers of resistance genuinely drive resistance or might represent abstractions of other potential drivers. To enhance the manuscript's clarity and address potential concerns about the model's selectivity and abstraction, we suggest providing more details and discussion in the main text.

The reviewer's observation that a large number of nodes are implicated in resistance pathways in Figure S10 is correct. However, we argue this is not a weakness of the model's selectivity, but rather a key finding that reflects the biological reality of adaptive resistance. The literature is replete with a wide and growing number of distinct mechanisms of resistance even to a single class of drugs (1,2), which supports the idea that cancer can co-opt a wide variety of network nodes to survive.

Figure S10 is not a binary map where every implicated node is equal, instead it is a likelihood map, where the colour and weight of the connections represent how often a particular interaction participates in driving resistance across the theoretical full range of possible network dynamics. The figure shows that while many nodes can contribute to resistance, they do so in a hub-like manner i.e. small subsets of nodes coordinate to drive resistance. This provides a rationalised, data-driven prioritisation of the most dominant and recurrent resistance strategies. We draw two important conclusions from this work (1) Resistance likely occurs due to resistance hubs, not individual proteins, and (2) that the frequency of a resistance hub in an MDN modelling analysis is likely proportional to the frequency of that hub emerging as a resistance mechanism in a population of cells and patients.

Regarding the issue of abstraction, the reviewer is correct that this is an inherent feature of any tractable systems model. In our case, several species in the mitogenic/cell-cycle pathways are module-level proxies to control model size. The highly implicated "hub" nodes in our model likely represent critical cellular processes that are themselves composed of several individual protein interactions.

To address these concerns, we have significantly revised the Discussion (page 16: lines 681 – 694) to: (1) frame resistance as a network-level phenomenon; (2) show that our frequency-based ranking is selective, prioritising the most probable, recurrent mechanisms; and (3) clarify that - given model abstraction -our findings implicate critical processes (modules), not just single proteins, as the drivers.

Overall, these changes do not alter our main conclusions: adaptive resistance is an emergent, network-level property; many routes exist, but a smaller set of nodes/modules consistently carry the largest influence across heterogeneous contexts.

(16) We consider that the figures and legends, including the supplementary information, are inadequately explained. The information provided is insufficient for us to comprehend the figures fully, leading to the need for interpretation on our part as readers. This could potentially introduce biases when trying to understand the claims made by the authors. To improve our understanding, it would be essential for the authors to assign appropriate labels to the figures and provide comprehensive explanations in the legends. For example, in Fig 3, we suggest labelling the tree diagrams in panels A and B, as well as the colour bars. We also recommend applying the same approach to other figures, adding accurate axis labels and descriptions of colour gradients to enhance clarity.

We thank the reviewer for this critical feedback. To address this comment, the figure legends have been revised where appropriate and greatly expanded to improve their comprehension. Moreover, we have added explicit labels to all previously unlabelled components, such as the cluster dendrograms and colour code bars in Figure 3A, B.

(17) To enhance readability, we recommend interchanging the order of Figures 1 and 2 in the sequence they appear in the main text. Alternatively, the text can be adjusted to refer to the figures in the correct order. Additionally, attention should be given to the bottom of Fig 1, which appears to be cropped or cut off. Furthermore, the incorrect word spacing in some figure elements, such as Fig. 3A title, Fig. 5B title, and Fig. 6B y-label, should be corrected for improved visual presentation.

Following the reviewer’s comment, the order of Figures 1 and 2 has been switched to reflect the order in which they are referred to in the main text. These Figures have been re-exported to fix unintentional word spacing errors.

(18) We recommend that the language used to refer to the initial conditions in the manuscript is clarified and homogenised. Currently, the authors use different terms such as "basal expression," "protein expression," "state variable values," or "initial conditions" to refer to them. This variation in terminology can be confusing for readers. In particular, the use of "basal expression" is problematic, as it typically refers to the leaky value of a reaction in the absence of an inducer, making it another biophysical parameter of the system rather than an initial condition. To enhance clarity and consistency, we suggest the authors decide on a single term to refer to the initial conditions throughout the manuscript and provide a clear explanation of its meaning to avoid any confusion. This will help readers better understand the concept being discussed and prevent any potential misinterpretations.

We thank the reviewer for this very helpful suggestion. To resolve this and improve clarity, we have homogenized the language throughout the manuscript. We now clarify the use the following 3 terms in their specific contexts:

We use “protein abundances” exclusively for the conserved total abundances of multi-state species (e.g., Xtot = X + pX + complexes) that are sampled across instances to represent expression heterogeneity.

We use ‘initial conditions’ to refer to initial values of the state variables in a model simulation. This term is related to protein abundance as the setting of initial conditions for conserved species sets the protein abundance. This is explicitly stated in the text (page 3: lines 87 - 91).

We use “state variables” to refer to the time-dependent model species.

We avoid the term “basal expression” in technical descriptions. Where a biology-facing phrase is helpful, we use “protein expression level”. This is used when referring to the biological concept that the initial conditions are intended to represent, i.e. the heterogeneity in protein amounts across a cell population.

We have performed a thorough search-and-replace to ensure this new convention is applied consistently and have removed the potentially confusing term "basal expression" from the revised manuscript.

(19) Why are saturable functions (e.g., Michaelis-Menten functions) ignored in the model? What are the potential consequences?

The main objective of this work was to perform a large-scale, systematic exploration of a high-dimensional parameter space (94 parameters) to map the full repertoire of qualitative dynamic behaviours a network topology can support. Using saturable functions like Michaelis-Menten kinetics would have roughly doubled the number of parameters to be explored (from k to Vmax and Km for each enzymatic reaction), making a parameter sweep of this scale computationally intractable. We therefore prioritised the breadth of the parameter search over the depth of kinetic detail, which we believe is the appropriate choice for a proof-of-concept study focused on heterogeneity.

This simplification has potential consequences. A major one is that our model cannot capture phenomena that arise specifically from enzyme saturation, such as zero-order kinetics or certain forms of ultrasensitivity (switch-like responses). However, we argue that this is an acceptable trade-off for two main reasons: (1) Our analysis is based on classifying broad, qualitative response shapes (increasing, decreasing, rebound, etc.). Mass-action kinetics are fully capable of generating this rich spectrum of behaviours; and (2) by varying the mass-action rate constants over nine orders of magnitude (from 10^-5^ to 10), our parameter sweep effectively samples a vast range of reaction efficiencies. A very low rate-constant can approximate the behaviour of a saturated, low-efficiency enzyme, while a high rate-constant can approximate a highly efficient, non-saturated one. In this way, the broad sweep of the rate parameter partially reflects the effects that would be captured by varying Vmax and Km.

For transparency, we have added a brief rationale to the “ODE model construction, modelling, and simulations” part of the Methods (revised main text, page 4: lines 153-155) and the "Description of Model Scope and Construction" section in the Supplementary file (Supplementary text page 2: lines 63-73).

(20) Given the relevance of the concept of "heterogeneity" in this work, a short discussion about biochemical noise and its implications on the analysis (e.g., why it is not included, and if it will be a next step) would be appreciated.

Our MDN modelling framework represents heterogeneity by creating an ensemble of deterministic models, where each model instance has a unique set of kinetic parameters and/or initial protein abundances. We propose that this is a powerful way to mechanistically represent the functional consequences of all sources of cellular variation. Over time, the effects of genetic mutations, epigenetic states, and even the time-averaged impact of intrinsic biochemical noise will manifest as changes in the effective interaction strengths and protein concentrations within a cell. Our large-scale parameter/IC sweep is designed to systematically explore the full range of dynamic behaviours that can emerge from this underlying biological variation. Therefore, our approach does not compete with stochastic modelling but is complementary to it. While stochastic simulations can capture the dynamic trajectories of single cells, our framework provides a panoramic view of the entire spectrum of possible stable phenotypes that can emerge at the population level. We agree that modelling intrinsic biochemical noise (stochasticity arising from finite copy numbers), e.g. using chemical Langevin or SSA, is a possible extension in future work but expected to be very computationally expensive. We have added a brief discussion on this as future direction in the revised Discussion.

(21) We have noticed that the first four paragraphs of the Discussion section overlap with the Introduction, as they mainly reiterate the significance of the study itself rather than focusing on the specific results obtained. To avoid redundancy and provide a more cohesive and informative discussion, we recommend that the authors shift the focus of the Discussion section towards presenting potential interpretations, even if they are not definitive, of the results obtained. By doing so, the Discussion will serve as a valuable platform for deeper analysis and insightful observations, allowing readers to better comprehend the implications and significance of the research findings.

We thank the reviewer for this structural feedback. Following the reviewer's feedback, we have significantly rewritten and restructured the Discussion section. The redundant introductory material has been removed.

The rewritten Discussion centres on interpretation, implications, and connect our findings to the literature. It now: (i) frames MDN as a systems-level framework that links molecular heterogeneity to qualitative signalling “meta-dynamics” and adaptive escape under constant drug pressure; (ii) highlights two key findings: an asymmetry in control (interaction kinetics exert stronger, more consistent influence than protein abundance) and a topology-driven convergence whereby a vast parameter space funnels into a finite set of recurrent behaviours; (iii) shows that resistance is a network-level property, with many possible routes but a small set of recurrent hubs/modules dominating; and (iv) provides a qualitative alignment with single-cell reporter data while clarifying the intent and limits of that comparison. Moreover, we now explicitly discuss limitations (rate-law simplifications, broad priors, determinism, and modular abstractions) and outline next steps for future research, including data-constrained priors and stochastic extensions.

We believe this substantial revision has transformed the Discussion into a much more insightful and valuable part of the manuscript that directly addresses the reviewer's concerns.

(22) The supplemental text file containing the model equations can be a bit challenging to read and understand. It would be greatly beneficial if the authors could consider generating a file using a typesetting program.

We have now included a typeset list of state variable equations and ODEs, along with the original model files.

(23) The authors mentioned that some model parameterizations result in negative solutions, which is surprising. Access to the model equations would help understand why this happens and is crucial for researchers who may want to use this approach. Clarifying the model equations' presentation would enhance transparency and aid other researchers in applying this method for similar research questions.ach. Clarifying the model equations' presentation would enhance transparency and aid other researchers in applying this method for similar research questions.

The reviewer is correct to be surprised by the mention of negative solutions, as negative concentrations are physically impossible. We clarify that these are not a result of any structural flaw in our model's equations but are a well-known, although rare, numerical artifact of floating-point arithmetic in computational solvers.

Our model is constructed using standard mass-action and first-order kinetics, which structurally guarantee non-negativity. However, when a species' concentration approaches the limits of machine precision (i.e., becomes a very small number extremely close to zero), the ODE solver can, in rare instances, numerically undershoot zero, resulting in a small negative value. If this occurs, it can lead to instability in subsequent integration steps.

This is not a biological phenomenon but a computational one. Therefore, the standard and appropriate procedure, which we follow, is to implement a filter that discards any simulation trajectory where such a numerical instability occurs.

(24) The reference listed for the CDK4/6 and CDK2 measurements is Yang et al. [55] in the figure caption, but as Xe et al. in lines 559-561 of the manuscript.

The text has been updated to match citation.

(25) We suggest that the authors revise and cite a previous study conducted by Yamada et al. (Scientific Reports, 2018), which presents an approach to expressing cell heterogeneity as a probability distribution of model parameters.

Following this suggestion, we have revised the Discussion (see response to comment (21)) to include and discuss Yamada et al. (Scientific Reports, 2018), which models cell heterogeneity as a probability distribution over parameter values.

(26) In the manuscript, on line 677, the authors state, "This indicates that there is an upper limit to the degree to which parameter sets can influence the qualitative shape of a protein's dynamic within a given network topology." We wish to highlight that this finding may not be particularly surprising. Given that the parameters were randomly determined within a specific range, it is understandable that altering the number of parameter samples would not substantially impact the distribution of model instances.

We thank the reviewer for this insightful comment, which allows us to clarify the significance of this finding. While it is true that any sampling from a fixed distribution will eventually converge statistically, our conclusion is not about statistics but about the intrinsic, constraining properties of the network's topology. The novelty is not that the distribution converges, but that it converges to a surprisingly limited and finite repertoire of qualitative dynamic behaviours. A complex, non-linear network with nearly 100 free parameters could theoretically generate an almost endless variety of complex dynamics. Our finding is that this specific biological topology acts as a powerful filter, robustly channelling the vast majority of the near-infinite parameter combinations into a small, recurring set of functional outputs (increasing, decreasing, rebound, etc.).

The reason for this finite limit is mechanistic, as the reviewer's comment prompted us to investigate further. Our parameter sweep already covers an extremely wide, 9-order-of-magnitude range. As we pushed parameter values to even greater extremes in exploratory simulations, we found they do not generate novel, complex dynamic shapes. Instead, they tend to drive network nodes into saturated states- either permanently "on" (maximally activated) or permanently "off" (minimally activated). In both cases, the node becomes unresponsive to upstream perturbations.

Therefore, further expanding the parameter range would be unlikely to uncover new behavioural categories; it would simply increase the proportion of model instances classified as "no-response." This demonstrates a fundamental principle: the network topology itself enforces an upper limit on its dynamic complexity. We think this inherent robustness is what allows for reliable cellular signalling in the face of constant biological variation. We believe this is a non-trivial finding, and we have revised the Discussion (page 16: lines 664 - 680) to state this conclusion and its implications more clearly.
